# Strategies towards Targeting Gαi/s Proteins: Scanning of Protein‐Protein Interaction Sites To Overcome Inaccessibility

**DOI:** 10.1002/cmdc.202100039

**Published:** 2021-03-22

**Authors:** Britta Nubbemeyer, Anna Pepanian, Ajay Abisheck Paul George, Diana Imhof

**Affiliations:** ^1^ Pharmaceutical Biochemistry and Bioanalytics Pharmaceutical Institute University of Bonn An der Immenburg 4 53121 Bonn Germany; ^2^ BioSolveIT GmbH An der Ziegelei 79 53757 Sankt Augustin Germany

**Keywords:** G alpha proteins, peptides, protein-protein interactions, signal transduction, small molecules

## Abstract

Heterotrimeric G proteins are classified into four subfamilies and play a key role in signal transduction. They transmit extracellular signals to intracellular effectors subsequent to the activation of G protein‐coupled receptors (GPCRs), which are targeted by over 30 % of FDA‐approved drugs. However, addressing G proteins as drug targets represents a compelling alternative, for example, when G proteins act independently of the corresponding GPCRs, or in cases of complex multifunctional diseases, when a large number of different GPCRs are involved. In contrast to Gαq, efforts to target Gαi/s by suitable chemical compounds has not been successful so far. Here, a comprehensive analysis was conducted examining the most important interface regions of Gαi/s with its upstream and downstream interaction partners. By assigning the existing compounds and the performed approaches to the respective interfaces, the druggability of the individual interfaces was ranked to provide perspectives for selective targeting of Gαi/s in the future.

## Graphical abstract

As Gαi/s proteins are involved in various diseases, five different Gαi/s interface regions were analyzed, and their druggable sites were assessed to provide a basis for tool and drug development. The Switch II/α3 region turned out to be well exposed and druggable, so can be used as target structure for developing selective and cell‐permeable Gαi/s modulators.

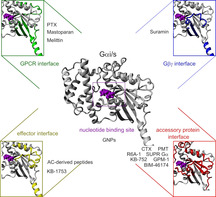

## 1 Introduction

**Figure 1:**
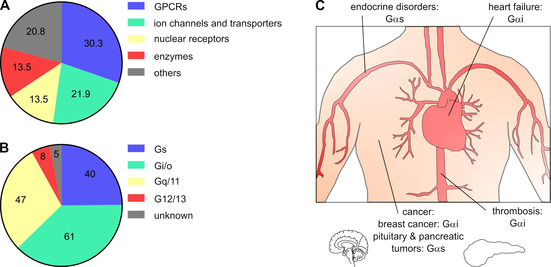
Involvement of GPCRs and G proteins in human diseases and drug development. A) Distribution of approved drugs (small molecules and biologics) per human protein family class derived from Santos et al.^[15]^ B) Putative primary Gα protein coupling, based on the classification of GPCR signaling according to Sriram et al.^[5]^ C) Involvement of Gαi/s subfamilies in multiple disorders such as cancer, heart failure, endocrine disorders or thrombosis, adapted from Li et al.^[1]^

G protein‐coupled receptors (GPCRs) represent the largest family of transmembrane receptors with more than 800 members controlling the signal transduction of physiologically important processes. Through extracellular stimuli of the GPCRs, the signal is transmitted via membrane‐bound, intracellularly localized heterotrimeric G proteins to intracellular effectors.[^1^, ^2^, ^3^] The indisputable importance of GPCR‐mediated signal transduction is demonstrated by the fact that over 30 % of the FDA‐approved drugs target GPCRs (Figure 1A).[^4^, ^5^] The attractiveness of addressing GPCRs lies in easily accessible druggable sites at the cell surface.[^4^, ^6^] GPCRs are targeted for numerous diseases, including Alzheimer's disease and cancer. In particular, oncogenic mutations of GPCRs and G proteins have been identified in a significant number of tumors.[^4^, ^7^, ^8^, ^9^, ^10^] As randomly mutated GPCRs can occur, it is difficult to develop drugs that respond to each of these mutations. Furthermore, multiple GPCR signaling pathways may be involved in multifactorial diseases, such as asthma or cancer, making it unsuitable to address the GPCRs individually.[^1^, ^2^, ^11^] Therefore, targeting the downstream G proteins may be an appropriate alternative, further strengthened by the fact that overexpression, abnormal activation, mutations, and dysregulation of G proteins are attributed with diseases such as cancer (Figure 1B, C).[^7^, ^8^, ^10^] Besides cancer, G proteins are also associated with cardiovascular diseases, for example, heart failure, diabetes, and chronic inflammatory diseases like asthma.[^1^, ^12^, ^13^]

G proteins are often referred to as “undruggable” because they cannot be adequately targeted pharmacologically.^[14]^ The intracellular location and the consequent lack of accessible sites on the cell surface is one of the reasons. Thus, molecules addressing G proteins need to pass the cell membrane to influence their activity. Of particular interest is the Gα subunit, which acts as a molecular switch by binding guanine nucleotide diphosphate (GDP, inactive) or guanine nucleotide triphosphate (GTP, active).

With respect to Gα, the four existing G protein subfamilies, Gαs, Gαi, Gαq/11, and Gα12/13 and their subtypes (Gαs: Gαs, Gαolf; Gαi: Gαi1‐3, GαoA/B, Gαt1‐2, Gαgust, Gαz; Gαq/11: Gαq, Gα11, Gα14‐16; Gα12/13: Gα12, Gα13), have a high sequence and structural similarity, making it difficult to selectively address only one subfamily.[^16^, ^17^, ^18^] The development of selective and efficient G protein activators or inhibitors (“modulators”) is of crucial importance, as they can be used as tools to gain deeper insights into G protein‐mediated signaling and as lead structures to design therapeutic drugs. In this regard, various strategies have been applied to identify and develop modulators of G protein activity. For example, the investigation of natural compounds led to the discovery of G proteins in 1980, for which A. G. Gilman and M. Rodbell were awarded with the Nobel Prize for Physiology and Medicine in 1994.[^19^, ^20^, ^21^] Another possibility for the identification of G protein modulators are high‐throughput screening techniques, which are commonly used to identify small molecules and peptides. Due to the structural similarity of the G protein subfamilies, small molecules might have only moderate target specificity, as can be exemplified with the imidazopyrazine derivatives BIM‐46174 and BIM‐46187.^[22]^ Nevertheless, small molecules are able to interact with proteins specifically on protein “hot‐spots”.^[23]^

G proteins generally communicate through protein‐protein interactions (PPIs) to regulate cellular processes.^[24]^ In this context, the disruption of PPIs can lead to a specific modulation of the protein activity.[^25^, ^26^] Thus, (macrocyclic) peptides are meanwhile regarded as suitable medium‐sized molecules to interrupt PPIs, while the requirement for cell penetration can be met by incorporation of cell‐penetrating peptide (CPP) sequences, as demonstrated for Cyclorasin 9 A5, targeting the small G protein KRas.[^25^, ^27^, ^28^, ^29^, ^30^, ^31^] Today, peptidic modulators can be identified by several methods, including (computational) structure‐based design or combinatorial approaches.[^32^, ^33^, ^34^, ^35^]

Concerning Gα proteins, only the Gαq subfamily can be addressed sufficiently by the two naturally occurring cyclic depsipeptides YM‐254890 and FR900359, which selectively inhibit the Gαq‐mediated signaling pathway and are widely used in pharmacological studies, such as in uveal melanoma or asthma research.[^1^, ^36^, ^37^, ^38^, ^39^, ^40^, ^41^] As modulators like FR900359 and YM‐254890 are still missing for Gαi and Gαs, we examined the existing strategies and developments to provide a comprehensive analysis of Gαi/s as targets for chemical tools as well as their interface regions (to GPCRs, Gβγ, effectors, accessory proteins), which are crucial for respective signal transduction pathways. Thus, this review aims at establishing the essential prerequisite for the future development of highly specific and potent modulators and tools for the investigation of G proteins and their involvement in diseases.

## 2 Gαi/s Interfaces: Determinants of G Protein Signaling

For the development of Gαi/s modulators, it is essential to understand their different signaling determinants (Figure S1 in the Supporting Information). A ligand binding to a GPCR results in conformational changes of the GPCR and the associated G protein and thus the GDP dissociation from the Gα subunit. The resulting empty‐pocket conformation has a very short lifetime due to the high GTP concentration within the cell, which facilitates rapid GTP binding to Gα.^[42]^ The latter induces the dissociation of the heterotrimer into GTP‐bound Gα and Gβγ, which can address different intracellular effectors (Figure S1).[^16^, ^17^, ^42^] The signaling is terminated by the intrinsic GTPase activity of Gα, which causes GTP hydrolysis to GDP and phosphate. Following reformation of the heterotrimer, the GDP‐bound G protein is restored to its original inactive state.[^16^, ^17^] Further accessory proteins such as AGS proteins (activators of G protein signaling) or RGS proteins (regulators of G protein signaling) can stimulate G protein signaling or accelerate its deactivation.[^43^, ^44^] AGS or RGS proteins can act as 1) GDIs (guanine nucleotide dissociation inhibitors), which stabilize the inactive, GDP‐bound state and thus inhibit the activation of G proteins,^[45]^ 2) GEFs (guanine nucleotide exchange factors), which can accelerate the exchange of GDP by GTP,^[45]^ 3) GEMs (guanine‐nucleotide exchange modulators), which have a bifunctional activity (GDI or GEF) depending on the G protein substrate,^[46]^ and 4) GAPs (GTPase accelerating proteins), which enhance GTP hydrolysis and thus terminate the Gα signaling (Figure S1).[^45^, ^47^]

Concerning the intracellular effectors (Figure S1), the Gαs subfamily stimulates the membrane‐bound adenylyl cyclase (AC), which catalyzes the formation of cyclic adenosine monophosphate (cAMP) from adenosine triphosphate (ATP). On the contrary, the Gαi subfamily members Gαi1‐3 and Gαz inhibit AC and consequently the formation of cAMP.^[48]^ Subsequently, cAMP can stimulate various downstream signaling pathways. Furthermore, Gαt1‐2 stimulates photoreceptor phosphodiesterase (PDE), Gαgust is thought to stimulate PDE activity and absence of Gαo was found to be associated with ion channels’ regulation.[^16^, ^48^, ^49^]

In order to map out possible directions for future strategies of Gα protein‐targeted compound design based on the proteins’ interface regions, it is required to analyze the structures of Gαi/s in the different activation states and ligand‐complexed forms. Several X‐ray and NMR structural analyses were reported in the past decades,[^16^, ^50^] starting from the crystal structure analysis of Gαt in the active, GTPγS (guanosine‐5′‐*O*‐(γ‐thio)triphosphate)‐bound state (1993), and the inactive, GDP‐bound state (1994).[^51^, ^52^] The Gα subunit has a conserved protein fold consisting of two domains, the GTPase domain (or Ras‐domain, six‐stranded β‐sheet motif (β1‐6) surrounded by five helices (α1‐5)), which is structurally homologous to small G proteins and elongation factors of the G protein superfamily, and the helical domain (six α‐helix bundle, with a large central helix (αA) surrounded by five smaller helices (αB−F)), which is unique for heterotrimeric G proteins (Figure S2).[^51^, ^52^] Both domains are connected by two polypeptide segments, linker 1 and linker 2, resulting in the following sequence of structural elements starting from the N‐terminal α‐helix (αN): αN, β1, α1, linker 1, αA‐F, linker 2, β2, β3, α2, β4, α3, β5, αG, α4, β6, α5.[^51^, ^52^] Only the α3‐β5 loop and the α4‐β6 loop of Gαi1 and Gαs differ in their sequence and structural conformation within the conserved GTPase domain, which possibly influences the Gα binding to GPCRs and effectors.^[53]^ The Gαi subfamily exhibits a high degree of conservation in sequence and structure, mostly distinguishable by minor differences in the helical domain.^[53]^ In between the two domains is a deep cleft, where the respective guanine nucleotide is bound (Section 2.2).[^51^, ^52^] Upon G protein activation, conformational changes occur in three adjacent regions, namely Switch I (linker 1, beginning of β2), Switch II (C‐terminus of β3, α2, α2‐β4 loop) and Switch III (β4‐α3 loop, Figure S2), which are mainly located in the GTPase domain.[^16^, ^51^, ^52^] All Gα subunits, except Gαt, are reversibly post‐translationally modified (PTM) with palmitate on a N‐terminal cysteine.^[16]^ Gαi subfamily members are additionally irreversibly myristoylated on an N‐terminal glycine, which has a significant influence on αN. The latter is disordered in the unmodified state and gets ordered upon Gβγ binding, while the ordered αN in case of a myristoylated Gαi results in no further structural change during Gβγ binding. Furthermore, myristoylation might affect the effector interaction (Sections 2.4 and 3.4). Overall, PTMs are important for the regulation of membrane association and PPIs.[^16^, ^17^, ^50^]

**Figure 2:**
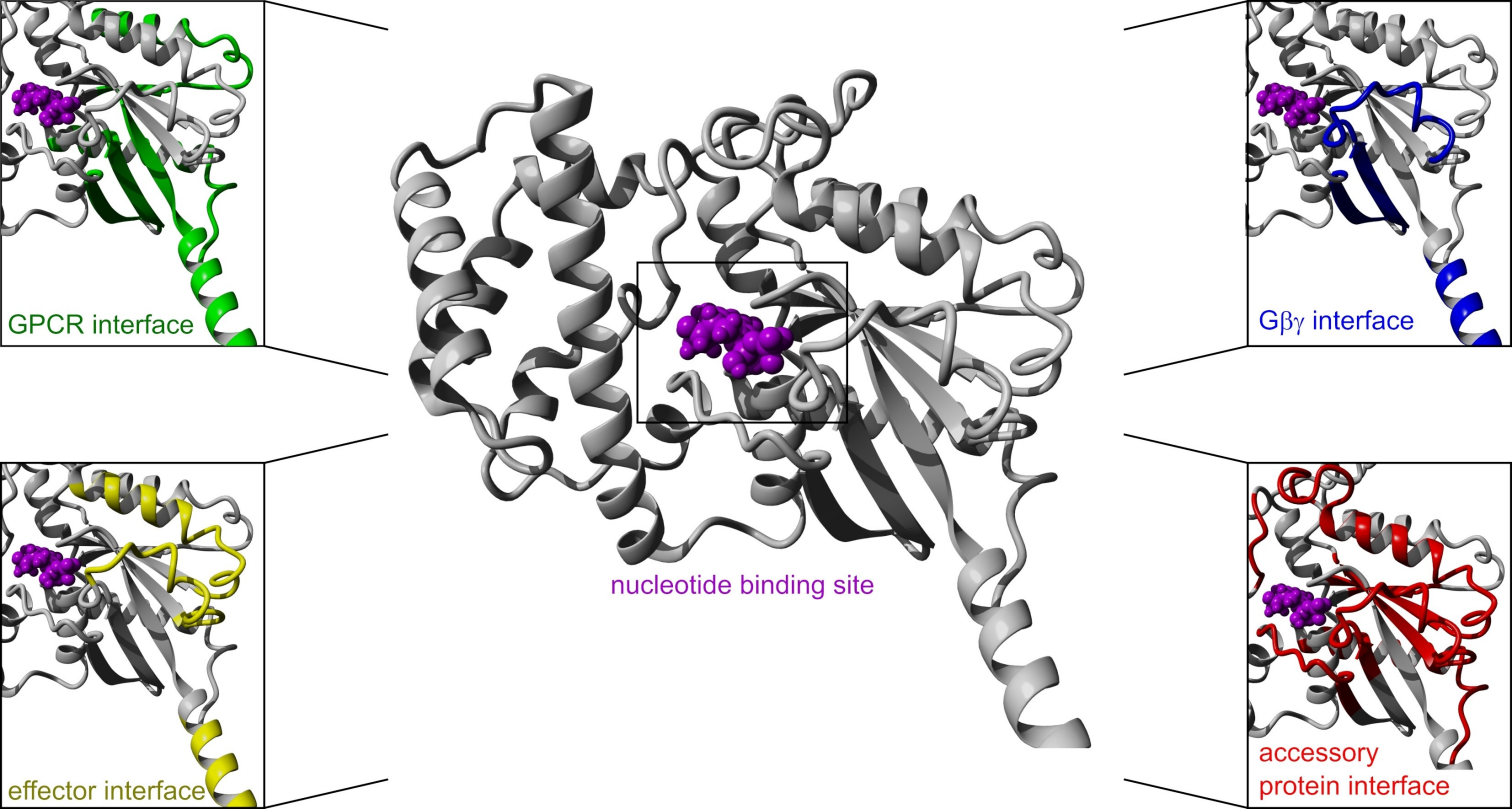
Structural features of Gα proteins: Contact areas to the GPCRs (green), Gβγ (blue), effectors (yellow) and accessory proteins (red, most common areas depicted) within the GDP‐bound (violet) Gαi1 homology model (from PDB IDs: 3UMS^[54]^ and 5JS8^[55]^).

The knowledge about the Gα structure supports the development of artificial modulators and the identification of natural products that influence the Gα protein activity. Therefore, it is helpful to know, that mostly the surface of the GTPase domain mediates interactions to GPCRs (Section 2.1), Gβγ (Section 2.3), downstream effectors (Section 2.4), and accessory proteins (Section 2.5, Figure 2).[^50^, ^53^] The composition of the nucleotide binding pocket and the GTPase mechanism (Section 2.2) essentially contribute to the development of new Gα protein modulators.^[44]^

In the following, we describe the individual interface regions and their impact on the G protein‐mediated signaling as well as the nature of the guanine nucleotide binding pocket in more detail. Our aim is to provide a more specific classification of the already known modulators (Section 3) by understanding the interface areas (Section 2), to assess the druggability of individual protein regions and thus to develop strategies for the identification of novel modulators.

### 2.1 Gαi/s‐GPCR

For their pioneering work on GPCRs, Robert J. Lefkowitz and Brian K. Kobilka were awarded with the Nobel Prize in Chemistry in 2012,[^56^, ^57^] which stresses the importance of G protein‐mediated signaling. GPCRs are characterized by seven transmembrane‐spanning α‐helices (TM1‐7), which are connected by three intracellular (ICL1‐3) and three extracellular loops (ECL1‐3). The N‐terminus is extracellular and the C‐terminus, which contains an α‐helix (HX8) in class A GPCRs, is located intracellularly (Figure S3).^[50]^ The TMs connect the extracellular ligand binding site with the intracellular binding site for the heterotrimeric G protein. Interestingly, the GPCR‐G protein interface is about 30 Å apart from the GDP binding pocket, thus allosteric conformational changes within the interface and Gα result in the receptor‐mediated GDP release. During reorganization of the cytoplasmic GPCR region upon receptor activation, the rotation and large outward movement of TM6 together with the rearrangements of TM1, TM4, TM5 and TM7 is characteristic.[^58^, ^59^, ^60^] This results in a cytoplasmic cavity, which can be occupied by the C‐terminus of the Gα subunit, especially the “wavy hook” (distal C‐terminus) and α5, after rotation and translation (Figure S3).[^50^, ^60^, ^61^, ^62^] The resulting GPCR‐Gα interface is formed predominantly by hydrophobic interactions between TM3, TM5‐7, ICL3, HX8, and the Gα C‐terminal part (α4, α4‐β6 loop, β6, α5). A second, less extensive interface is established between αN, αN‐β1 hinge, β1, β2‐β3 loop, α5, and ICL2 (Figure S3). In addition, further Gα interactions (α3‐β5 loop, α2, α2‐β4 loop) with the GPCRs are described.[^24^, ^50^, ^58^, ^60^, ^63^]

Regarding the GPCR‐G protein coupling selectivity, a significant difference between Gi‐ and Gs‐GPCR complexes is the relative position of α5 (different rotation and orientation within Gαi/s) and TM6 (outward movement less intense for Gi‐ than for Gs‐coupled GPCRs). This results in a wider open G protein binding pocket for Gs‐coupled receptors and enables the binding of the sterically larger C‐terminus of Gαs (α5 tilted up), whereas α5 of Gi binds relatively further down in the TM pocket allowing capping interactions with TM7/HX8.[^58^, ^59^, ^60^, ^61^, ^62^, ^63^, ^64^] Consequently, the Gα C‐terminus is mainly responsible for the affinity and specificity of the G protein‐GPCR interaction.[^50^, ^65^, ^66^] Beside α5, an impact of αN, the αN‐β1 loop, the α4‐β6 region, and α4 on the specificity of G protein coupling has been suggested, due to specificity determining residues within these regions.[^24^, ^50^] Furthermore, TM6, ICL2 and ICL3 were related to mediate the coupling selectivity.[^50^, ^59^, ^61^, ^63^]

### 2.2 Gαi/s‐nucleotide

**Figure 3:**
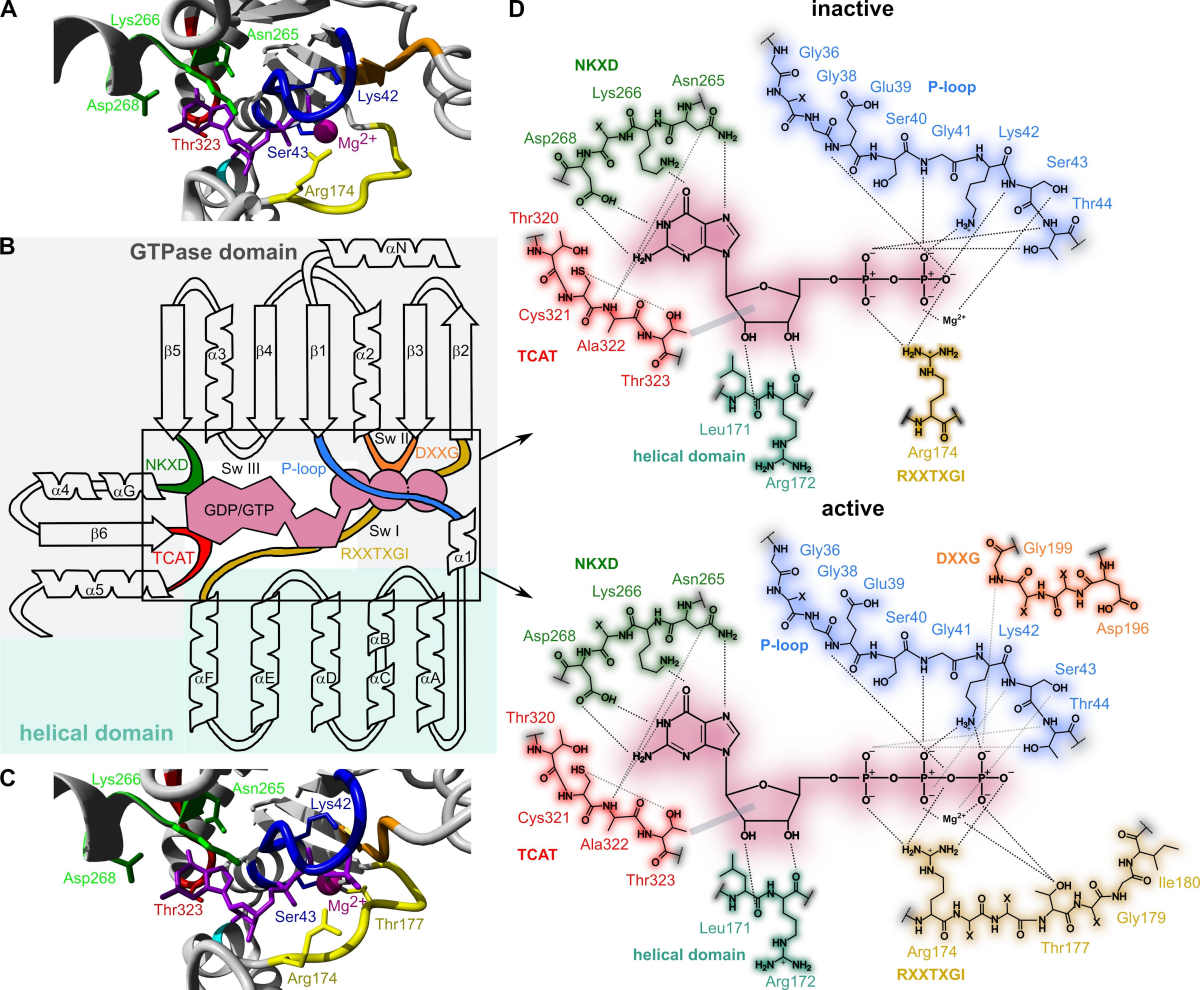
Contacts of Gα to bound nucleotides. Gαt crystal structures (GDP‐bound: PDB ID: 1TAG^[52]^ (A), GTPγS‐bound: PDB ID: 1TND^[51]^ (C), nucleotides in violet), domain arrangement^[84]^ of Gα proteins (B) and contacts of nucleotides (D) to the P‐loop (blue), RXXTXGI (yellow), DXXG (orange), NKXD (green), TCAT (red), helical domain (cyan) are shown. Dotted lines indicate hydrogen bonds and grey bars van der Waals interactions. Residues are named according to the crystal structures.

G proteins are called molecular switches, switching between the GDP‐bound (“off”) and the GTP‐bound (“on”) state to regulate the downstream signaling.[^1^, ^16^] The determinants of nucleotide binding are based on the architecture of the binding pocket (Figure 3), which structurally alters within 1) GDP release and formation of the empty‐pocket conformation, 2) GTP insertion and heterotrimer dissociation, 3) the GTPase reaction, and 4) the phosphate release together with the heterotrimer reassociation. In the following, the Common Gα Numbering system in the D.S.P. format (D: domain, with G: GTPase domain, H: helical domain; S: consensus secondary structure, with S: strand, H: helix; P: position within the secondary structure element, all in superscriptions) according to Flock et al.^[67]^ is used to describe the involved Gα residues and to facilitate a comparison between the different Gα subtypes and subfamilies. Loops are written as lower case letters of the flanking secondary structure elements.^[67]^

The guanine nucleotide binding pocket is located deep in the core of Gα between both domains (Figure 3).[^51^, ^52^] The nucleoside contacts are formed by interactions with both domains, whereas the phosphate contacts are mainly established with linker 2 and the GTPase domain.[^52^, ^68^] Two conserved motifs, the NKXD^G.S5.7‐G.HG.2^‐motif and the TCA(T/V)DT^G.s6h5.1‐G.H5.1^ motif (“TCAT‐motif”) are crucial for the binding of the guanine base and the stabilization of GDP in the binding pocket.[^16^, ^69^] The phosphate binding is mediated by the highly conserved P‐loop, GXGESGKST^G.s1h1.1‐G.H1.3^, which connects β1 with α1. Furthermore, the RXXTXGI^G.hfs2.2‐G.S2.1^ motif and the DXXG^G.S3.7‐G.s3h2.2^ motif are important for Mg^2+^ binding, whereby the latter motif connects the Mg^2+^ binding site with Switch II.[^16^, ^67^, ^69^, ^70^, ^71^, ^72^] Mg^2+^ is octahedrally surrounded by ligands and coordinated by four water molecules, Ser43^G.H1.2^ (P‐loop) as well as the β‐phosphate in the inactive state.[^51^, ^52^, ^73^]

*GDP release and formation of the empty‐pocket conformation*. For GDP dissociation, domain separation is required along with the destabilization of the GDP‐binding contacts mediated by GPCR‐induced conformational changes inside the G protein.[^58^, ^72^, ^74^, ^75^, ^76^, ^77^] The conformational changes in α5 cause structural rearrangements of the adjacent β6‐α5 loop (contains TCAT motif, Figure 3) and the reduction of hydrophobic interactions between α5 and α1, β2, and β3, and thus a destabilization and structural change of α1 (contains P‐loop, Figure 3). As a consequence, the interface between the helical domain and the GTPase domain is disrupted and the GDP affinity is reduced.[^58^, ^76^, ^78^, ^79^, ^80^] However, the reduced contacts of α5 with β1‐3 are compensated by new interactions to β4‐β6, which stabilize the receptor‐bound complex.^[80]^ Beyond that, the αN‐β1‐loop contributes significantly to GDP dissociation by disturbing P‐loop contacts to GDP.[^17^, ^58^, ^72^, ^76^] The GDP release is favored as a result of the reduced GDP contacts along with a higher structural dynamic in the nucleotide‐binding region.[^58^, ^72^] In the resulting ternary complex, the helical domain exhibits increased dynamics and moves away from the GTPase domain.^[76]^ In addition, the structure of the nucleotide binding pocket, especially the β6‐α5 loop, is more dynamic and exhibits a larger solvent‐accessible surface area, which promotes fast GTP binding induced by the high intracellular GTP concentration.^[81]^

*GTP binding and dissociation of the heterotrimer*. GTP binding leads to stabilization of α1 and the interdomain interface and induces the reclosure of both domains to a more rigid Gα structure.[^55^, ^63^, ^76^, ^80^] Herein, Mg^2+^ and GTP are deeply buried in the binding pocket due to rearrangements of Switch I (Arg174^G.hfs2.2^, Thr177^G.hfs2.5^, RXXTXGI motif), Switch II (Gly199^G.s3h2.2^ and α2), and Switch III (Figure 3A, C, F).^[69]^ The structural changes within Switch I are induced by hydrogen bond formation between the γ‐phosphate of GTP with Thr177^G.hfs2.5^ and Arg174^G.hfs2.2^, and the replacement of two water ligands on Mg^2+^ by Thr177^G.hfs2.5^ and the γ‐phosphate.[^52^, ^68^] The conformational change of Switch I towards the Mg^2+^ binding site causes the interruption of Gα‐Gβγ interactions and thus contributes to the dissociation of the heterotrimer. The structural changes in Switch I and Switch II are connected through a newly formed hydrogen bond network.[^52^, ^68^] Rearrangements in Switch II are initiated by a hydrogen bond formation between Gly199^G.s3h2.2^ and the γ‐phosphate of GTP, which is coupled to conformational changes of α2 conveyed by a hydrogen bond of Gly198^G.s3h2.1^ with Trp207^G.H2.7^. During this process, contacts of the conserved Arg201^G.H2.1^, Arg204^G.H2.4^ (ion pairs with Glu241^G.H3.4^, Switch III) and Trp207^G.H2.7^ to conserved residues in α3 are formed.[^52^, ^68^] Switch III (e. g., Glu232^G.s4h3.10^, Glu241^G.H3.4^) responds to the conformational changes of Switch II by forming a network of polar interactions with Arg201^G.H2.1^, Arg204^G.H2.4^, and the Gly199^G.s3h2.2^.[^52^, ^73^] Additional residues within the β4‐α3‐loop and α3 stabilize the active conformation of Switch III through interaction with the helical domain.^[73]^ The GTP binding leads to a destabilization of the heterotrimer, mainly by changes within Switch II, and initiates dissociation into Gα and Gβγ (Section 2.3).^[73]^

*GTPase reaction*. During GTP hydrolysis, the highly conserved Arg174^G.hfs2.2^ (“arginine finger”, Switch I, RXXTXGI motif) decisively stabilizes the pentavalent transition state by interacting with the β‐ and γ‐phosphates of GTP (Figure 3D).[^68^, ^82^] Additionally, the highly conserved Gln200^G.s3h2.3^ (Switch II) is essential for the hydrolysis by interacting with the γ‐phosphate and the nucleophilic water, which initiates the in‐line attack on the γ‐phosphate.[^68^, ^83^] Hence, mutations of Arg174^G.hfs2.2^ or Gln200^G.s3h2.3^ have been observed in a number of human tumors, demonstrating the importance of these residues and the GTPase reaction for the G protein signaling.^[82]^ Within the hydrolysis mechanism, the water molecule is further stabilized by the Thr177^G.hfs2.5^.[^68^, ^69^, ^70^, ^83^] RGS proteins are able to accelerate the GTPase activity (Section 2.5).

*Dissociation of phosphate and heterotrimer reassociation*. In the resulting Gα⋅GDP⋅Pi complex, Switch I moves marginally away from the catalytic site leading to a weaker Mg^2+^ binding and a hydrogen bond formation of Arg174^G.hfs2.2^ with the β‐phosphate and Pi, as well as Thr177^G.hfs2.5^ and Lys176^G.hfs2.4^. Switch II undergoes a significant structural change, which breaks the ionic interactions with Switch III, resulting in a disordered Switch III. Thereby, Gln200^G.s3h2.3^ is shifted away from the active center, a transient phosphate binding site is formed and the Pi release is enabled.^[83]^ The latter results in disordered parts of the Switch II and thus, Switch I shifts away from the nucleotide binding site, whereby Lys176^G.hfs2.4^ rotates out of the active center, along with Mg^2+^ and Thr177^G.hfs2.5^. Then, Arg174^G.hfs2.2^ is only weakly associated with the α‐ and β‐phosphate.^[83]^ As Switch II is crucial for effector recruitment and Gβγ binding (Section 2.3, 2.4), the structural changes in Switch II reduce the affinity towards the effectors and promote Gβγ binding.^[73]^ The binding of Gβγ rearranged Switch II and, furthermore, the conformational changes within Switch I and Switch II seal the GDP in the nucleotide binding pocket.^[83]^

### 2.3 Gαi/s‐Gβγ

Gβγ is composed of two polypeptide chains, Gβ and Gγ, which can only be separated under denaturing conditions.[^18^, ^85^] Crystal structure analyses revealed that Gβ exhibits an N‐terminal α‐helix and a seven bladed propeller structure composed of seven WD40 sequence repeats with four twisted β‐strands per propeller blade (Figure S4). Gγ comprises two α‐helices, with the N‐terminal helix binding to the N‐terminal helix of Gβ via coiled‐coil interactions and the C‐terminal helix engages with the propeller. The membrane association is controlled by prenylation of the Gγ C‐terminus.[^85^, ^86^, ^87^, ^88^] The contacts between Gα and Gβγ are primarily made via two interface regions between Gα and Gβ (Figure S4). The first interface is established between the top of the Gβ propeller by hydrophobic interactions with the hydrophobic pocket of Gα formed by Switch I and Switch II (especially β2, β3, β3‐α2 loop, α2, Figure S4). This interface is additionally stabilized by hydrophilic/ionic interactions. The second interface is located between blade 1 of the Gβ propeller and αN of Gα. There is no structural evidence for direct interactions of Gα and Gγ.[^53^, ^85^, ^86^, ^87^, ^88^] The structure of Gα in the heterotrimer differs from free Gα.[^86^, ^87^] In the heterotrimer, the αN helix is continuous, whereas in the free state the N‐terminus can exhibit various structures.[^86^, ^87^] The myristoylation of the N‐terminus increases the affinity of Gα to Gβγ (Section 2).^[89]^ The GTP‐induced conformational changes especially in Switch II (Section 2.2) lead to the heterotrimer dissociation by interruption of the stabilizing contacts within the first interface.[^85^, ^86^, ^87^, ^88^]

### 2.4 Gαi/s‐effector proteins

Crystal structure experiments of Gα‐effector complexes showed that the effectors insert hydrophobic side chains into a pocket formed by the N‐terminus of α2 (Switch II) and α3. The effector specificity is defined by contacts with the C‐termini of α2 and α3 as well as interactions with the α2‐β4 loop and the α3‐β5 loop.[^16^, ^49^, ^53^, ^90^, ^91^, ^92^] Since the α3‐β5 loop differs in sequence and structure between the subfamilies, it was assumed that it plays the key role in effector selectivity.[^49^, ^53^] A further contribution of the α4‐β6 loop was also reported.[^16^, ^53^, ^90^, ^93^]

The Gαi and Gαs subfamily can interact with different effectors, however, both subfamilies have an opposite effect on the AC, whereby Gαs can bind to and activate all membrane‐bound isoforms of AC (ACI‐IX) and Gαi1 and the near paralogs can only address certain AC isoforms (ACI, V, VI).[^90^, ^94^, ^95^, ^96^] The AC consists of a cytosolic N‐terminus, two transmembrane domains separated by the cytosolic domain C1 (C1a–b), and followed by a further cytosolic domain C2 (C2a–b, Figure S5). The active site is located in the interface between C1 and C2.^[97]^ The Gαs‐AC interface is established between Switch II (α2 and α2‐β4 loop) by insertion of α2 into the groove of AC (formed by C2), and the α3‐β5 loop with C1 and C2. At the same time, Phe991(C2) binds into the Switch II/α3 cleft.[^91^, ^92^, ^93^, ^95^] Mutagenesis experiments and molecular docking studies indicate that the Gαi‐AC interface is located between C1 and Switch I–III as well as αB, which is opposite to the Gαs binding site on AC (Figure S5). Thus the binding of Gαs and Gαi to the AC is not competitive.[^53^, ^90^, ^93^, ^98^] Further studies with Gαs and Gαt showed that the N‐terminus is crucial for effector binding. In the Gαs subfamily, no PTM is necessary for the stimulatory function, whereas myristoylation of the Gαi subfamily is required for AC inhibition.[^16^, ^53^, ^97^, ^99^, ^100^]

After GTP hydrolysis, Gα dissociates from AC due to a lower affinity of the Gα⋅GDP compared to Gα⋅GTP. Although Gα⋅GDP still has the ability to interact with effectors, its potency is lower than that of Gα⋅GTP. Reassociation with Gβγ terminates effector signaling since the Gα binding site for Gβγ (inactive state, Section 2.3) largely overlaps with the effector binding site (active state).^[16]^

### 2.5 Gαi/s‐accessory proteins

Accessory proteins are capable of interfering with the G protein signaling in different ways, in particular by binding to Gα (Figure S6) and thus modulating the Gα activity. AGS proteins are divided into classes I–IV with I) GEFs (all Gα subclasses), II) GDIs (Gαi‐selective), III) Gβγ binders or IV) Gα16‐specific.[^43^, ^101^, ^102^, ^103^] RGS proteins are categorized into different structural and functional classes, which are named after the prototypical member (i. e. A/RZ (Gαz/i‐specific), B/R4 (Gαi/o/q‐specific), C/R7 (Gαi/o‐specific), D/R12 (Gαi/o‐specific)). Typically, such proteins act as GAPs preferably with the Gαi subfamily.[^44^, ^103^, ^104^] In the following, the structural aspects of 1) GDIs, 2) GEFs, 3) GEMs and 4) GAPs are described in more detail.

*GDIs*. GDIs comprise one to four GPR motifs (G protein regulating motif, TMGEEDFFDLLAKSQSKRMDDQRVDLAG,[^105^, ^106^] also known as GoLoco motif, consensus XXΦΦXΩΩX[+]XQπXRΩXXQR,[^107^, ^108^] Φ: hydrophobic, Ω: aromatic, π: small, X: any amino acid)). The GPR motifs bind to and stabilize Gαi⋅GDP, thereby inhibiting the nucleotide exchange and the accompanied G protein activation (Figure S6). GDIs can prevent the association of Gα with Gβγ through overlapping interface regions, which may lead to prolonged Gβγ signaling.[^45^, ^103^, ^108^, ^109^] The binding of the GPR motif is directed to Switch II/α3, where Arg of the Asp/Glu−Gln−Arg triad of the GPR motif is oriented towards the GDP binding pocket and directly interacts with the α‐ and β‐phosphate of GDP.^[45]^ The insertion of Arg is enabled by the conformation of Gln (triad), which interacts with Gln147^H.hdhe.2^ and Asn149^H.hdhe.4^ of Gαi. The GPR motif also establishes contacts to Switch I and changes its conformation, for example, Arg178^G.hfs2.2^ (RXXTXGI motif, Section 2.2) is displaced by a salt bridge with Glu43^G.s1h1.1^ (P‐loop) and forms contacts to the GDP ribose entity. Further conformational changes occur in Switch II and Switch III. The C‐terminal part of the GPR motif binds along the interdomain region, thus possibly restricting interdomain movements and preventing GDP dissociation.[^102^, ^108^, ^109^, ^110^, ^111^] Gαi specificity is assumed to be mediated by contacts with the helical domain (αA‐αB loop, αB‐αC loop),[^102^, ^108^, ^109^, ^110^, ^111^] and/or an acidic residue in the GTPase domain that influences the orientation of Glu43^G.s1h1.1^.^[112]^

*GEFs*. The chaperones for nucleotide‐free Gα subunits Ric8 A (resistance to inhibitors of cholinesterase, Gαi/q/12/13‐specific) and Ric8b (Gαs/olf‐specific) also function as GEFs through partial Gα unfolding (in absence of Gβγ).[^43^, ^113^, ^114^] They bind preferentially to Gα⋅GDP, cause GDP dissociation by domain separation and stabilize the empty pocket conformation, although GTP binding leads to Ric8 dissociation due to a lower binding affinity (Figure S6).[^114^, ^115^] Three Gα contact sites for Ric8 proteins were referred: α5, β4–6 and Switch II/α3 together with the P‐loop.[^113^, ^114^, ^116^] Similar to GPCRs, Ric8 interaction leads to a major structural changes of α5 and detachment from the hydrophobic β‐sheet core (β4–6), which also rotates and is then stabilized by Ric8. The α5 movement disrupts the nucleotide contacts of the TCAT motif and the NKXD motif and destabilizes the purine binding site (Section 2.2). The antiparallel β2–β3 hairpin moves away from the GTPase core, which destabilizes and disordered α1 and thus leading to domain separation of Gα, destabilization of the P‐loop contacts to GDP and enhanced GDP dissociation.[^113^, ^116^, ^117^, ^118^] The interaction of Ric8 A probably shifts Switch II to the binding position of the γ‐phosphate, which is associated with conformational changes in Switch I and promotes GTP binding.[^116^, ^117^, ^118^] The interruption of the contacts between Switch II and Ric8 A during GTP binding leads to the reorganization of β2 and β3, and Ric8 A dissociation. The selectivity determinants of Ric8 are probably family‐specific residues of Gα (α5), whereby the majority of Ric8 A and Ric8B residues are conserved in the Gα contact region.[^113^, ^116^, ^117^, ^118^]

*GEMs*. GEMs are the most recently discovered class of G protein‐affecting proteins, with GIV (Gα‐interacting, vesicle‐associated protein) being first described as GEM (GEF for Gαi, GDI for Gαs).[^46^, ^119^] GEMs possess a common motif (∼30 amino acids, core consensus ΦTΦX[D/E]FΦ‐motif,^[120]^ Φ: hydrophobic, X: any amino acid) that selectively binds to the GDP‐bound or empty‐pocket conformation and affect monomeric Gα (Figure S6).[^84^, ^121^] So far, only the GEF binding to Gαi3 has been structurally analyzed. The binding of the GEM motif to the cleft formed by Switch II (mainly contacts with Gln204^G.s3h2.3^, Trp211^G.H2.7^, Phe215^G.h2s4.1^), α3 and the α3–β5 loop, induce conformational changes in Switch I (RXXTXGI motif), β1, and the P‐loop and thus in the phosphate binding, which is sufficient for Gα activation.[^84^, ^121^] Allosterically induced conformational change of the β2‐β3 loop with associated α5 movement and disturbances in the interdomain interface (Switch III, αD–αE loop) is also observed, with the latter potentially resulting in domain separation.[^84^, ^121^] The binding site of the GEM motif partially overlaps with the GDI and the Gβγ binding site.[^84^, ^121^]

*GAPs*. GAPs interact with Gα⋅GTP and are able to catalyze GTP hydrolysis by stabilizing the transition state. The respective RGS proteins contain a functionally conserved RGS domain (∼120 [B1] amino acids, “RGS box”), which is responsible for the Gα interaction and the catalytic activity.[^45^, ^103^, ^122^] The RGS domain forms an interface to Gα, recognizing and stabilizing mainly residues in Switch I‐III (Figure S6). Three critical contacts are reported: 1) A hydrogen bond between Asn128 (RGS4) and Gln204^G.s3h2.3^ (Switch II), which orients Gln204^G.s3h2.3^ (Section 2.2) to stabilize the γ‐phosphate and the nucleophilic water molecule. Asn128 also interacts with Switch II, thus stabilizing the conformation of Switch I and II. 2) A hydrogen bond between Asn88 (RGS4) and Thr182^G.hfs2.6^ (Switch I), which brings Switch I–II into the conformation of the transition state, thereby Thr182^G.hfs2.6^ (Switch I) gets in contact with Lys210^G.H2.6^ and Glu207^G.H2.3^ (Switch II). 3) Asp163 (RGS4) stabilizes Thr182^G.hfs2.6^ (Switch I), allowing the adjacent Thr181^G.hfs2.5^ (Switch I) to stabilize the Mg^2+^ and to bring the nucleophilic water into an ideal position for GTP hydrolysis.[^44^, ^91^, ^104^, ^122^, ^123^] RGS contacts with Switch III and the helical domain (αA, αB‐αC loop) are differently pronounced in the subtypes of the Gαi subfamily and possibly contribute to Gα selectivity and the potency of GAP activity.[^91^, ^104^, ^122^, ^124^, ^125^, ^126^, ^127^] The binding side of RGS proteins is consistent with the fact that RGS proteins are antagonists for effectors.[^122^, ^127^] The specificity of the Gαi subfamily compared to the RGS‐GAP incompetent Gαs subfamily can be explained by differences in the primary structure of the switch regions.[^91^, ^104^, ^122^, ^124^, ^125^]

## 3 Modulators Targeting Gαi/s Interfaces

The analysis of the Gα interface regions demonstrates that the contact regions are predominantly located in the GTPase domain (especially Switch I–III, β‐sheet core, α3, N‐ and C‐terminus). The helical domain is crucial for the nucleotide exchange and may serve as a specificity feature within the Gα subfamilies, as Gαi subfamily members are mostly distinguishable by minor differences in the helical domain.^[53]^ The analysis also reveal which regions are exposed at the Gα surface and can be targeted by potential modulators. For example, Switch II/α3 may be regarded as “druggable” because it is addressed by Gβγ (Section 2.3), effectors (Section 2.4), and accessory proteins (Section 2.5). The latter show that binding to this region may have a functional impact on Gα and therefore represents an interesting model for modulator development (Section 3.5). Additionally, α5 (important for G protein activation, allosteric connection to nucleotide binding pocket), and αN (important in GPCR coupling, Gβγ binding and PTMs), are also interesting target structures (Section 3.1, 3.3). In the following, the individual interfaces are investigated for already known Gα binders and/or modulators as well as their identification methods. The classification of the individual interfaces according to their druggability provides important perspectives for future modulator development.

### 3.1 Gαi/s‐GPCR

Within the Gα‐GPCR interface, the C‐terminus (wavy hock, α5) and the N‐terminus (αN, αN‐β1, β1) play significant roles in the allosterically induced GDP release (Figure S3). The essential function of the C‐terminus for the GPCR coupling as well as its selectivity was recognized very early. For this reason, antibodies targeting the C‐terminus of the Gα subunit were developed (Supporting Text in the Supporting Information, Figure S10).

#### 3.1.1 Natural compounds

**Figure 4:**
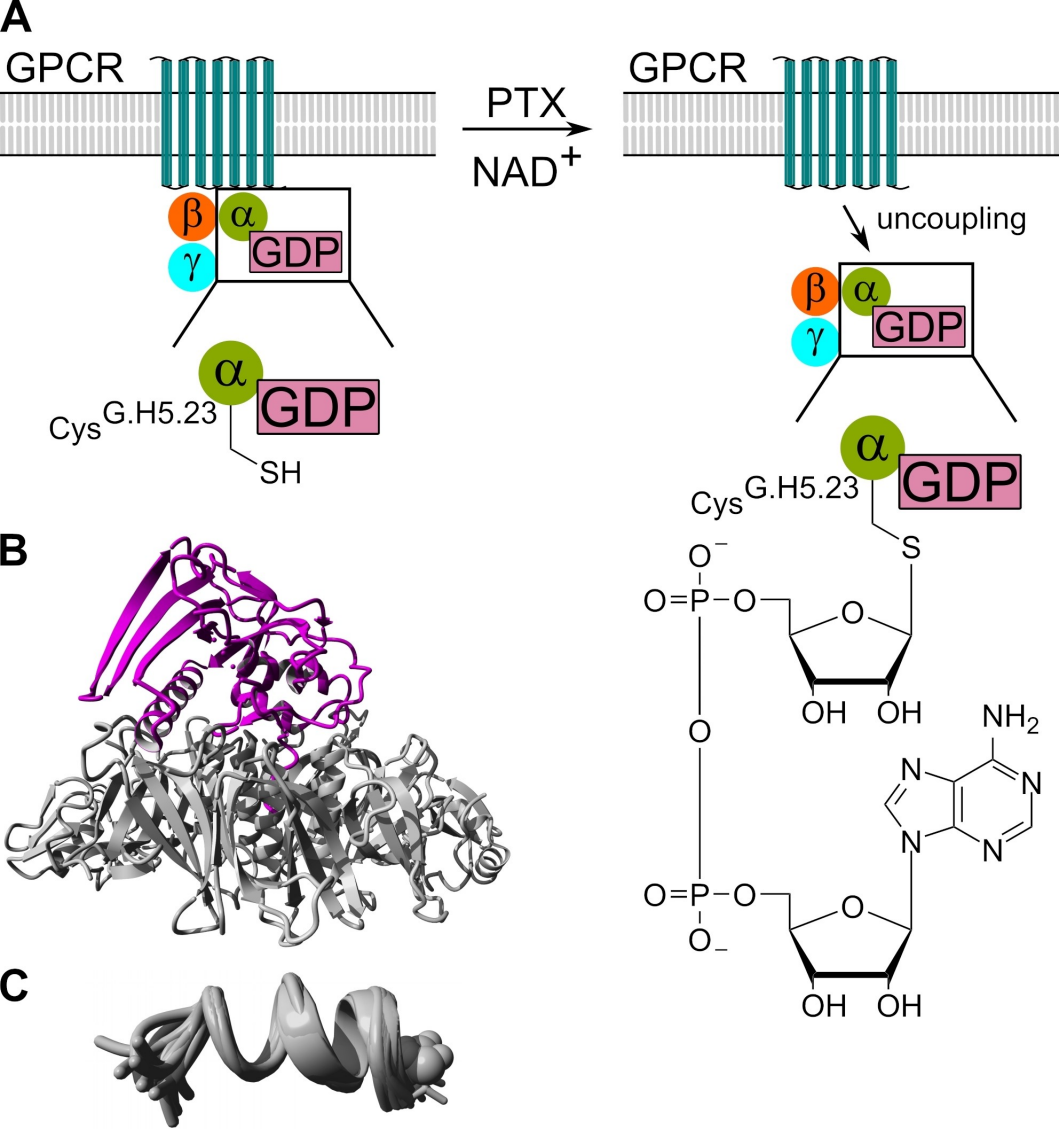
Natural compounds targeting the Gα‐GPCR interface. A) Modification of Gαi by pertussis toxin (PTX) derived from Mangmool et al.^[129]^ PTX transfers the ADP‐ribose element from nicotinamide adenine dinucleotide (NAD^+^) to Gαi Cys^G.H5.23^. B) Crystal structure of PTX (gray, PDB ID: 1PRT^[128]^). The S1 subunit (magenta) is important for Gαi inhibition. C) G protein‐bound NMR structure ensemble (14 structures) of mastoparan‐X (H‐INWKGIAAMAKKLL‐NH_2_, PDB ID: 1 A13^[133]^).

A number of natural compounds have been described for the Gα‐GPCR interface. These include a bacterial exotoxin and numerous cationic amphiphilic substances, such as venom peptides from bees or wasps, whereby the latters can reversibly influence the Gα protein activity (Figure 4).

Pertussis toxin (PTX, 105 kDa^[128]^), is an exotoxin from *Bordetella pertussis* and inhibits the Gαi subfamily (except Gαz, Figure 4A, B). It can exert a mono‐ADP‐ribosyl transferase activity, covalently and irreversibly transferring an ADP‐ribose element from nicotinamide adenine dinucleotide (NAD^+^) to the C‐terminal Cys^G.H5.23^ conserved in the Gαi subfamily. Consequently, Gi uncouples from the receptor, cannot be activated, and remains GDP‐bound leading to cAMP accumulation and various pathological effects in the host cell.[^1^, ^21^, ^128^, ^129^, ^130^] In addition, G protein‐independent actions have also been described, which renders PTX together with its irreversible modification incapable for clinical use. Nevertheless, PTX has been applied in numerous studies to analyze Gαi‐specific effects.[^1^, ^129^, ^131^, ^132^]

A variety of cationic, amphiphilic substances, including neuropeptides, hormones, venom peptides, and polyamines, exhibited activating properties on purified G proteins. They have a high proportion of hydrophobic and basic groups orienting in an amphipathic α‐helical structure in the presence of phospholipids (Figure 4C), and allowing them to penetrate the cell membrane.[^134^, ^135^] Prominent members of this group are the wasp venom 14mer peptide mastoparan (H‐INLKALAALAKKIL‐NH_2_) and the bee venom 26mer peptide melittin (H‐GIGAVLKVLTTGLPALISWIKRKRQQ‐NH_2_). Both venom toxins are able to disrupt cell membrane phospholipids and to cause lysis.[^131^, ^136^, ^137^, ^138^, ^139^]

Mastoparan and related analogs (mastoparans) increase the rate of GTP binding in a GEF‐like manner and the GTPase activity for Gi/o, but have only a weak effect on Gt and Gs (except mastoparan‐S, H‐INWKGIASM‐α‐aminoisobutyryl‐RQVL‐NH_2_).[^131^, ^133^, ^134^, ^136^, ^140^, ^141^] Mastoparan has been shown to engage the Gα N‐ and the C‐terminus and competes with GPCRs for G protein binding and thus has been used as low‐molecular‐weight GPCR mimetic.[^133^, ^142^, ^143^, ^144^, ^145^, ^146^, ^147^] Melittin comprises a predominantly hydrophobic N‐terminus and a hydrophilic C‐terminus. It stimulates Gi activity and inhibits Gs activity, which consequently leads to inhibition of AC activity.[^139^, ^148^, ^149^] Furthermore, activating effects on G proteins and their GTPase activity were reported for the neurokinin substance P (H‐RPKPQQFFGLM‐NH_2_), synthetic polyamine component 48/80 (C48/80, mixed polymer of *p*‐methoxy‐*N*‐methyl phenylethylamine crosslinked by formaldehyde), the mast cell degranulating peptide (H‐IKCNCKRHVIKPHICRKICGKN‐NH_2_, MCD), and other cationic amphiphilic substances.[^132^, ^134^, ^136^, ^142^, ^150^, ^151^, ^152^, ^153^, ^154^, ^155^, ^156^, ^157^] Altogether, these compounds are considered as pharmacological tools and candidates with potential therapeutic applications.[^137^, ^158^] In the context of Gα modulators, the broad use of compounds such as melittin and mastoparan, is restrictive because of their dose‐ and cell‐type dependency, nonspecific targeting and thereby induction of various biochemical effects.[^159^, ^160^]

In summary, the natural compounds interact mainly via the Gα C‐terminus, which appears well exposed and druggable, and thus cause GPCR‐G protein uncoupling. For PTX, this results in a permanent inhibition of Gi, whereas the cationic amphiphilic peptides lead to GPCR‐independent activation and signaling. The latter is a valuable starting point for tool development at the G protein level, which circumvents the need to address many GPCRs in multifactorial diseases.

#### 3.1.2 Synthetic compounds

The described modulators from natural sources revealed that cationic hydrophobic substances are able to act as G protein modulators. Thus, these compounds have been further investigated. One synthetic compound is the polyamine C48/80 (Section 3.1.1), which activates Gi/o and stimulates GTPase activity.[^141^, ^142^] In addition, other cationic hydrophilic substances such as hydrophobic amines[^136^, ^157^] or derivatives of the lead mastoparan[^136^, ^138^, ^161^] have also been described as Gα modulators.

Quaternary hydrophobic amines have been referred in the context of mastoparan and can affect the activity of purified recombinant G proteins. For example, benzalkonium chloride (BAC) antagonizes the Gi stimulation of mastoparan by inhibiting the GDP exchange, whereas BAC alone slightly increases the basal GDP exchange at high concentrations. In contrast, BAC and other quaternary amines has been suggested to stimulate the nucleotide exchange and the GTPase activity of Go in response to the phospholipid concentration.^[136]^ Other quaternary long‐chain alkylamines displayed equally stimulatory properties on Go, whereas short‐chain amines were ineffective. However, high concentrations of hydrophobic amines destabilize the G protein and might lead to denaturation.[^136^, ^157^] Overall, these amines are considered unsuitable for the modulation of Gα protein activity, since they may also bind unselectively to other proteins and influence their activity.

In numerous studies, various derivatives of mastoparan (synthetic and natural) were investigated to explore the structural determinants, including net charge, spacing, charge localization, and proportion of α‐helical conformation (Figure 4C), which define activity and cytotoxicity of the lead.[^136^, ^138^, ^147^, ^161^, ^162^] To reduce the cytotoxicity of mastoparan towards mammalian cells, [I^5^, R^8^]‐MP was developed by replacing Ala5Ile and Ala8Arg, resulting in antimicrobial activity against bacteria and fungi but no cytotoxicity in HEK293 cells or hemolytic effects towards human erythrocytes.^[138]^ Consequently, mastoparan is a prototype substance for the derivation of valuable antiinfective agents from naturally occurring antimicrobial peptides. However, due to G protein‐independent side effects, these compounds are less attractive as G protein modulators.^[138]^ In addition to mastoparans, GPCR‐derived peptides have been extensively studied in order to gain insight into G protein‐GPCR coupling and coupling selectivity.[^163^, ^164^, ^165^, ^166^] These GPCR‐derived peptides, however, have a comparably low potential, since each peptide can only interfere with the G protein signaling of a few receptors possessing, for example, similar ICL regions.

In summary, although the Gα‐GPCR interface appears to be druggable, the existing modulators for this interface have many drawbacks for application as tool compounds. The interface might not be well suited for selective Gα targeting, due to the fact that there are multiple GPCRs adressing the same Gα subfamily. Thus, the selective modulation of one distinct Gα protein within the Gα‐GPCR interface requires different modulators to affect one G protein signaling cascade entirely. Apart from this, this interface shows potential for exploiting the different coupling selectivities of a GPCR to a Gα protein to selectively affect a special GPCR‐Gα interaction. In this context, however, it appears easier to address the extracellular druggable sites of a GPCR.

### 3.2 Gαi/s‐nucleotide

The nucleotide binding pocket is not a typical PPI interface like the other regions described, wherein, different guanine nucleotides (GNPs, Figure S7) are able to bind. As GNPs are not classical modulators and can bind unspecific to other guanine nucleotide‐binding proteins, we will only briefly discuss them here. More detailed information can be found in the supporting information. One application of GNPs is the ability to induce different activity states, as demonstrated by various crystal structure experiments and studies for quantifying the percentage of active G protein.[^51^, ^52^, ^68^, ^167^, ^168^] Altogether, GNPs represent crucial tools for the analysis of G protein‐affecting compounds, as they can be used, for example, in radioactive or fluorescently labeled form, to determine the impact of the tested compound on the nucleotide exchange as well as on the GTPase activity.[^167^, ^169^, ^170^] Consequently, GNPs proofed to be efficient for various applications.[^51^, ^52^, ^68^, ^167^, ^168^, ^169^, ^170^]

### 3.3 Gαi/s‐Gβγ

There are not many modulators that address the Gα–Gβγ interface by approaching Gα, thus we decide not to subdivide this section. As shown in Section 2.3, Gα contacts Gβγ on the switch regions and αN (Figure S4).^[86]^ The G protein activation enables the heterotrimer dissociation, whereby upon reassociation, the signaling is terminated since the effectors and Gβγ share Gα binding sites (Section 2.3, 2.4).[^87^, ^171^, ^172^] Furthermore, AGS class II proteins, such as AGS3 (contains four GPR motifs, Section 2.5), are able to dissociate the heterotrimer, since the GPR motif attaches and changes the conformation of Switch II close to the Gα‐Gβγ interface. Consequently, modulators identified or developed for the Gα‐accessory protein interface may also affect the Gα‐Gβγ interaction (Sections 2.5, 3.5, Figure S6).[^109^, ^173^, ^174^, ^175^] Moreover, Gβγ seems to compete with the fluorescently labeled Alexa532‐RGS4 protein for binding with high affinity to Gαi⋅GDP⋅AlF4^−^, which implies that Gβγ can inhibit the action of GAPs by binding to Gα.^[176]^ Apart from that, the prenylation of Gγ (Section 2.3) anchors Gβγ in the plasma membrane and is highly required for the interaction with Gα and effectors.[^177^, ^178^, ^179^]

Based on the G protein signaling partners, peptides that bind to Gα on the Gα‐Gβγ interface were developed. Kimple et al.^[109]^ exploited the RGS14 GoLoco region to design R14GL (DIEGLVELLNRVQSSGAHDQRGLLRKEDLVLPEFLQ) derived from rat RGS14 (also accessory protein interface), that binds to Gαi between Switch II and α3 but not to Gαo, whereas the interaction with Switch II imbricates the contact of Gαi1⋅GDP and Gβγ.^[109]^ Subsequently, Wang et al.^[182]^ developed a Gβ‐derived peptide exhibiting the respective Gαi1‐binding sequence of a second Gβγ binding site on Gα, which was able to interrupt the respective Gαi1⋅GDP‐Gβγ association.^[182]^

**Figure 5:**
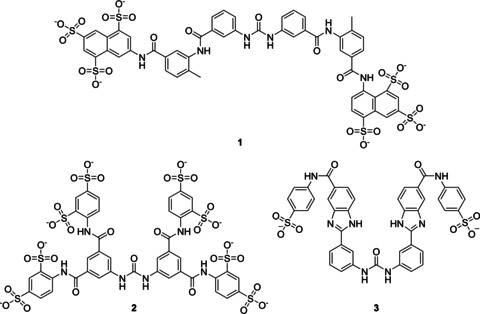
Chemical structures of suramin (**1**) and its analogues: NF449 (**2**) and NF503 (**3**).[^1^, ^180^, ^181^]

In addition to the natural partners within G protein signaling, researchers intended to study PPIs by targeting the Gα‐Gβγ interface via different screening approaches. In this regard, Gβγ modulators have also been developed, however, are not described herein.[^85^, ^180^] Suramin (**1**, Figure 5) is a drug discovered by Bayer in 1916 and used to treat the African sleeping disease. Initial studies implied that suramin binds directly to Gαs, hinders the heterotrimer reassociation and thus the G protein–receptor coupling.[^1^, ^183^, ^184^] Afterwards, experiments revealed that suramin inhibits the GDP release from Gα. However, suramin exhibits reduced selectivity, since it can inhibit Gαi and Gαs.^[1]^ Consequently, different suramin analogs have been developed such as NF449 (**2**) and NF503 (**3**, Figure 5), which were superior to the other, comprising a higher selectivity for Gαi and Gαs.[^1^, ^2^, ^181^, ^183^, ^185^, ^186^, ^187^] The structural basis and the pharmacological importance of these agents needs to be more specified in the future. A further suramin derivative (NF023, Figure 7, Section 3.5.2.1.) was identified to target the Gαi3‐GIV binding site.^[188]^ A major drawback of these compounds is their limited cell penetration due to the high negative charge of the sulfonic acid groups, thus decreasing their pharmacological potential.^[2]^

Based on the aforementioned reports, it can be concluded that this interface overlaps with the Gα‐effector and ‐accessory protein interface which hamper a clear distinction. Thus, these common sites might be valuable targets for future therapeutic applications.[^180^, ^189^]

### 3.4 Gαi/s‐effector proteins

Effectors of Gα are enzymes, proteins or ion channels with AC belonging to the most important effectors, which can be affected by Gαi and Gαs (Section 2.4, Figure S5).[^48^, ^53^, ^190^, ^191^] As already mentioned, Gαi myristoylation is required for its inhibitory effects to distinct AC isoforms.^[99]^ These findings provide a precious opportunity to modulate the Gα protein activity with PTM‐like modifications. Apart from that, natural molecules that impair the association of Gαi/s and their downstream effectors are rare. Only accessory proteins, such as RGS16 (Section 2.5), can be given here since they may act antagonistically with respect to G protein‐effector binding. In this regard, RGS16 was shown to bind to Gαt/o⋅GDP⋅AlF_4_^−^ affecting the Gαt/o signaling pathway by inactivating the G protein‐effector binding.[^104^, ^192^, ^193^] Based on these observations, the discovery of natural compounds or PTMs is anticipated to broaden the knowledge about this interface.

Likewise, there are only few examples of synthetic compounds that address this interface, which is why we have not divided this section further. It was already known in the 1970s that forskolin (Fsk) activates AC in a receptor‐independent way.[^93^, ^190^] What is striking though, is the contribution of the Fsk‐Gαs⋅GTPγS complex in raising the binding affinity to two AC analogs, VC1 (ACV) and IIC2 (ACII) and their catalytic activity (Figure S8).^[93]^ Furthermore, Yoo et al.^[194]^ constructed AC‐derived peptides and found that a peptide encoding C2‐α’2 (899–926), and two more peptides, namely C1‐β4‐β5‐α4 and C2‐α3’‐β4’, possessed inhibitory features regarding Gαs stimulation on full length ACII and ACVI (69 % inhibition for the C1‐peptide and 89 % for the C2‐peptides). Despite the aforementioned peptides, additionally tested peptides exhibited higher IC_50_ values, whereas others showed no inhibition.^[194]^

In summary, although crystal structures have provided insights into the Gαi/s effector binding,[^90^, ^93^] the availability of compounds acting on this interface is rather low.^[194]^ A possible explanation could be that the Gα‐effector interface is not easy, if not impossible, to be manipulated. On the other hand, this interface overlaps partially with the interface for accessory proteins (Section 2.5, 3.5), making it non‐trivial to clearly separate these regions. In our opinion, this interface may not be the most critical in studying G protein modulators, however, should not be neglected.

### 3.5 Gαi/s‐accessory proteins

Accessory proteins themselves are modulators of Gα protein activity, acting as GDI, GEF, GEM, or GAP (Section 2.5, Figure S6).[^45^, ^46^] Therefore, they serve as important templates for modulator development based on the motifs that are critical for their function and the interface that they bind to. Addressing the Gα‐accessory protein interface and the GTPase activity, respectively, was of enormous importance in the past, as inhibition of the Gαs GTPase function by cholera toxin (CTX, Section 3.5.1) led to the discovery of G proteins.^[21]^ Nowadays, accessory proteins have also been considered as drug targets, which is described in numerous excellent reviews.[^44^, ^103^, ^195^, ^196^, ^197^]

#### 3.5.1 Natural Compounds

**Figure 6:**
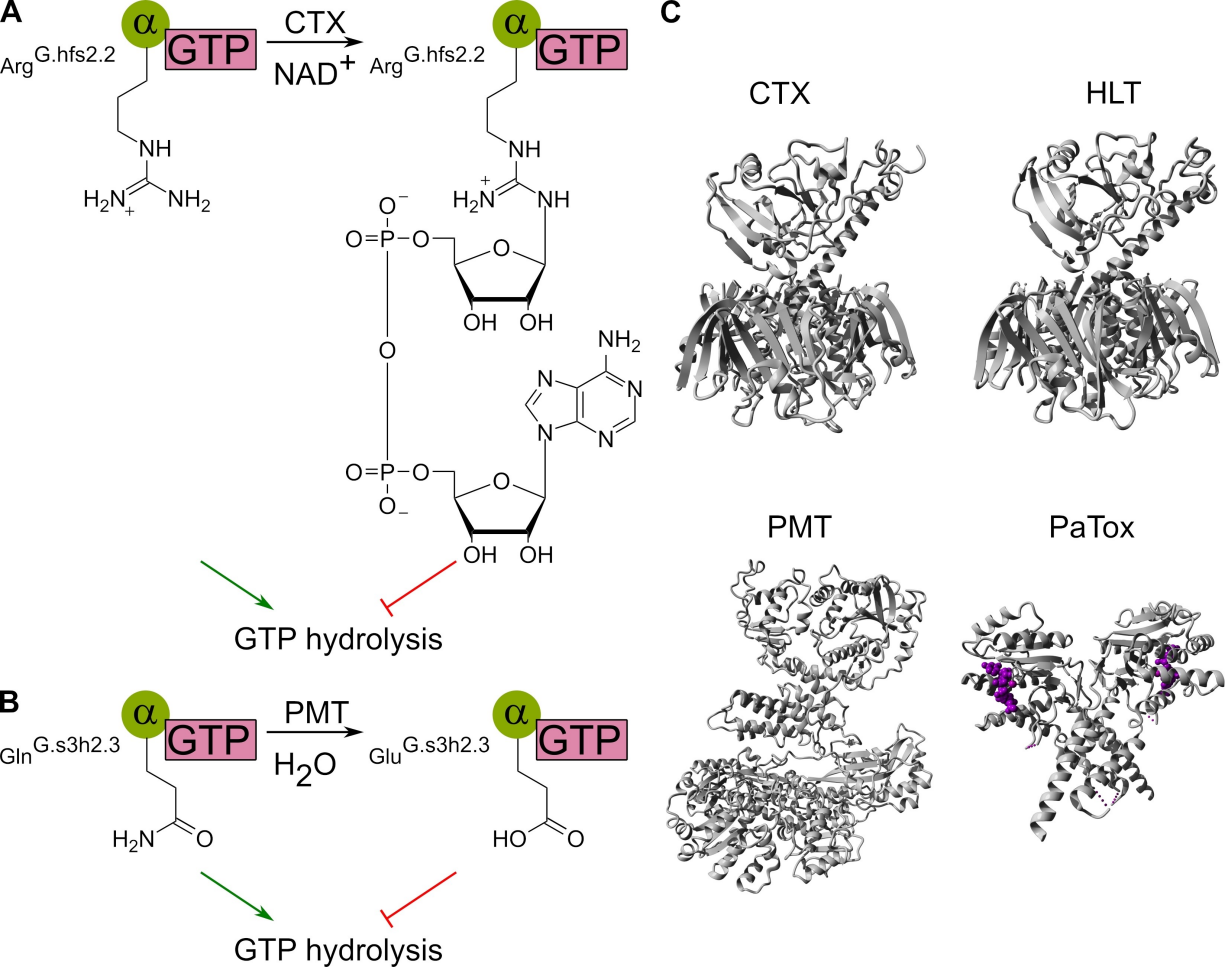
Natural compounds targeting the Gα‐accessory protein interface. A) Modification of Gαs by cholera toxin (CTX). CTX transfers the ADP‐ribose element from nicotinamide adenine dinucleotide (NAD^+^) to Arg^G.hfs2.2^ of Gαs, thereby inhibiting GTP hydrolysis. B) Modification of Gαi by *P. multocida* toxin (PMT). PMT catalyzes the deamidation of Gln^G.s3h2.3^ to Glu^G.s3h2.3^ and thus inhibits GTP hydrolysis. C) Crystal structures (gray) of cholera toxin (CTX, PDB ID: 1XTC^[213]^), heat‐labile enterotoxin (HLT, PDB ID: 1LTS^[207]^), *P. multocida* toxin (PMT, PDB ID: 2EC5^[214]^) and the *P. asymbiotica* protein toxin (PaTox) glycosyltransferase domain (PDB ID: 4MIX^[212]^) in complex with UDP‐GlcNAc (violet).

Regarding natural compounds targeting the Gα‐accessory protein interface, it is important to consider that Gβγ (inactive state) and effectors (active state) represent natural competitors for the binding of accessory proteins, since the interface within Gα overlaps significantly (Section 2.3, 2.4, 3.2, 3.4).[^16^, ^45^, ^49^, ^109^, ^121^, ^122^, ^127^] Furthermore, bacterial exotoxins directly affect the GTP hydrolysis.^[198]^ Cholera toxin (CTX, 84 kDa,^[199]^ Figure 6A, C) is an exotoxin from *Vibrio cholerae*, the bacterium responsible for the symptoms of the cholera disease.^[21]^ In early studies, it was observed that CTX increased the intracellular cAMP level by a permanent Gαs activation, leading to the discovery of G proteins.^[21]^ The activation was caused by a mono‐ADP‐ribosyl‐transferase activity of CTX (similar to PTX, Section 3.1.1), irreversibly transferring an ADP‐ribose element from NAD^+^ to Arg201^G.hfs2.2^ (arginine finger, Section 2.2) of Gαs (Figure 6A).[^1^, ^21^, ^193^, ^198^, ^200^, ^201^, ^202^] As a consequence, the GTPase activity is inhibited and Gαs⋅GTP is prevented from being inactivated.[^202^, ^203^, ^204^, ^205^] Using a similar mechanism, a heat‐labile enterotoxin (HLT, 86 kDa,^[206]^ Figure 6C) from *Escherichia coli* also selectively modifies and permanently activates Gαs.[^1^, ^201^, ^206^, ^207^] Furthermore, a toxin from *Pasteurella multocida* (PMT, 146 kDa,^[208]^ Figure 6B–C) modulates the Gα protein activity of Gαi/q/13. PMT catalyzes the deamidation of Gln205^G.s3h2.3^ (Gαi) and conversion to Glu205^G.s3h2.3^, thereby blocking the GTP hydrolysis (Section 2.2, Figure 6B). Consequently, Gαi remains in the active state resulting in a decrease in cAMP level.[^1^, ^82^, ^209^, ^210^, ^211^] PMT preferentially interacts, unlike PTX (Section 3.1.1), with monomeric Gα and can prevent conversion with PTX by Gαi deamidation.^[211]^ In addition, *Photorhabdus asymbiotica* protein toxin (PaTox, 335 kDa, UniProt: C7BKP9, Figure 6C) causes the Gln205^G.s3h2.3^ (Gαi) deamidation of Gαi/q/11 analogous to PMT and is also capable of catalyzing tyrosine glycosylation of Rho.^[212]^ However, all of these bacterial exotoxins have the disadvantage to unrecoverably modify Gα, thereby irreversibly affecting the G protein activity. Therefore, these modulators have less clinical utility and should rather be regarded as important pharmacological tools that can provide insights into immunological processes or different aspects of G protein signaling.^[201]^ However, it cannot be denied that targeting the GTPase function is a reasonable approach for modulating the Gα activity, since an inhibition maintains the Gα subunit in the active state whereas stimulation accelerates the termination of the signaling pathway.

#### 3.5.2 Synthetic compounds

The enormous potential of the Gα‐accessory protein interface has been recognized with the result that the development of novel tool compounds (small molecules and peptides) was primarily directed towards this interface region. High‐throughput techniques, but also virtual design, have been increasingly applied to identify or design novel modulators. Structure‐activity relationships derived from crystal structures of complexes or molecular modeling and docking were frequently performed, too.[^188^, ^215^, ^216^, ^217^]

##### 3.5.2.1 Small molecules

**Figure 7:**
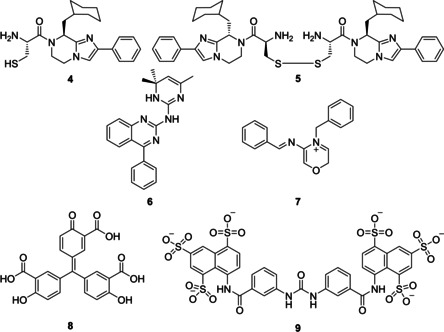
Chemical structures of small molecules targeting the Gα‐accessory protein interface. Imidazopyrazine derivatives BIM‐46174 (**4**) and BIM‐46187 (**5**),^[11]^ compounds 0990 (**6**) and 4630 (**7**),^[223]^ aurintricarboxylic acid (ATA, **8**) and suramin derivative NF023 (**9**).^[188]^

The development of small molecule modulators is a classical approach in medicinal chemistry. In 2006 and 2009, the imidazopyrazine derivatives BIM‐46174 (BIM‐monomer, **4**) and the disulfide‐bonded BIM‐dimer BIM‐46187 (**5**, both in short: BIM, Figure 7) were introduced, which showed antiproliferative and pain relief effects, respectively, and thus have been proposed as potential anticancer drugs.[^11^, ^218^, ^219^, ^220^] For the selection of G protein‐directed modulators, a differential screening approach with human cancer MCF‐7 cells was applied, comparing the influence of potential modulators on CTX‐stimulated cAMP production (Gαs‐mediated signaling) with the influence on Fsk‐stimulated AC activity (Section 3.4).^[218]^ Both compounds act as pan‐inhibitors of Gα protein activity, preferentially silencing Gαq signaling in a cellular context‐dependent manner.[^22^, ^220^] At the molecular level, BIM reversibly binds to Gα⋅GDP and prevents GTP binding after GDP dissociation.[^11^, ^22^, ^220^] Consequently, Gα is pharmacologically frozen in the empty‐pocket conformation.^[22]^ Using docking experiments and all‐atom molecular dynamics simulations, Switch II, Switch III, and the αB‐αC loop were postulated as BIM binding regions, which could explain the BIM‐mediated inhibition through conformational changes in the switch regions that are crucial for GTP binding as well as a restricted domain separation of helical domain and GTPase domain.[^11^, ^22^] In further studies, BIM was further analyzed with respect to Gαq targeting due to the Gαq preference.[^221^, ^222^]

In a computer‐based approach performed in 2014, molecular docking was applied to identify potential small molecules with GDI activity that bind to and stabilize Gαi⋅GDP in the presence of Gαi⋅GTP, Gαq⋅GDP, and Gαq⋅GTP.^[223]^ Two compounds (0990 (**6**) and 4630 (**7**); Figure 7) with GDI selectivity for Gαi1 over Gαq, three compounds (8005, 8770, 4799) with GDI selectivity for Gαq over Gαi1, and three compounds (2967, 6715, and 1026) with GDI activity towards Gαi1 and Gαq were identified.[^11^, ^223^] Some of these compounds were able to partially block the α2‐adrenergic receptor‐mediated cAMP regulation promoted by Gαi/o activation, however, neither compound showed the desired inhibitory activity even at high concentrations.[^1^, ^223^] The quinazoline derivative 0990 was studied in more detail and was suggested to bind to Gαi⋅GDP (Arg178^G.hfs2.2^/Val199^G.S3.6^ or Glu43^G.s1h1.1^/Gln79^H.HA.14^ or Gln79^H.HA.14^/Lys180^G.hfs2.4^), all mimicking important Gαi1‐GDI interactions. In structure‐activity relationship studies, the basic hydrophobic phenyl‐quinazoline‐aniline core was shown to be crucial for the GDI activity.[^11^, ^223^]

In 2017, by an *in silico* ligand screening and a separate high‐throughput screening, the Gαi3‐GIV interface (Section 2.5) was addressed, and NF023 (**9**, suramin derivative, Section 3.3) and ATA (**8**, aurintricarboxylic acid, both Figure 7) were identified. Both compounds were confirmed as Gαi3 binder and inhibitor of Gαi3‐GIV binding.^[188]^ NF023 binds to Switch II, α3 and α3‐β5 loop, a binding site that overlaps with the binding site of the GEM motif (Section 2.5).[^84^, ^120^, ^121^] However, no interference with Gαi3−Gβγ binding was observed, although the interface regions partially overlap (suggested for suramin, Section 3.3).^[188]^

The disadvantage of these small molecules is that NF023 (and suramin) are not cell permeable and can inhibit P2X receptors in addition to Gα subunits, and ATA can also address other targets such as topoisomerase II.[^1^, ^188^] Apart from that, the authors concluded that the Gαi–GIV interface is defined and druggable and thus of interest for modulator design.^[188]^

The screening approaches employing small molecules demonstrate the possibility to develop Gα modulators. However, a clear drawback is the selectivity of the compounds for the individual subfamilies or G proteins themselves. This is exemplified with BIM, a pan‐inhibitor for Gα protein activity, obtained from a screening experiment towards Gαs, while the approach from 2014 identified compounds with Gαi/q selectivity that did not exhibit the anticipated inhibitory activity. NF023 and ATA also address other targets besides Gα and are therefore not specific. Nevertheless, small molecules are important tools to study G protein signaling pathways and to explore the determinants for selectivity between the subfamilies.

##### 3.5.2.2 Peptides

The approach of peptide engineering is of particular interest regarding the Gα‐accessory protein interface. For example, peptide sequences derived from protein motifs, such as the GPR motif,[^106^, ^107^] GEM motif,[^84^, ^120^] and RGS domain,[^104^, ^122^] which are important for the corresponding functions as GDI,[^106^, ^107^, ^108^] GEM[^119^, ^120^] or GAP,^[122]^ can serve as templates for the peptide design.[^45^, ^46^]

GPR proteins and GPR‐derived peptides were shown to act as GDIs for Gαi *in vitro*.[^1^, ^102^, ^224^, ^225^] Subsequently, CPPs such as a hydrophobic K‐FGF‐derived peptide sequence (AAVALLPAVLLALLA) or basic TAT‐derived sequence (GRKKRRQRRRPP) were attached N‐terminally to a GPR motif (H‐TMGEEDFFDLLAKSQSKRMDQRVDLAK‐NH_2_) to increase the cell penetration of the GPR peptide.^[223]^ The TAT‐GPR construct maintained GDI activity and selectively blocked Gαi regulation of α2‐adrenergic‐mediated AC activity in HEK293 cells.^[223]^ The TAT‐GPR construct has therefore been proposed as a valuable pharmacological tool and potential therapeutics. The authors, however, have tended to consider the development of small molecule inhibitors (Section 3.5.2.1) due to the relatively large size of the construct (40mer peptide).^[223]^ In a similar approach, a GIV‐derived peptide (GIV‐CT, 210 amino acids), containing the GEM motif and an SH2‐like domain, was N‐terminally coupled to a TAT‐PTD (peptide transduction domain) sequence to increase cell permeability.^[226]^ It has been shown that the construct can bind to Gi in a cellular context and activates it in a GEF‐dependent manner.^[226]^ Consequently, peptides derived from accessory protein motifs can affect the Gα protein activity and intracellular modulation can be achieved by CPP attachment. The drawback to the described constructs is that they are relatively large as to be used as chemical tools (e. g., 40mer peptide or protein).

**Figure 8:**
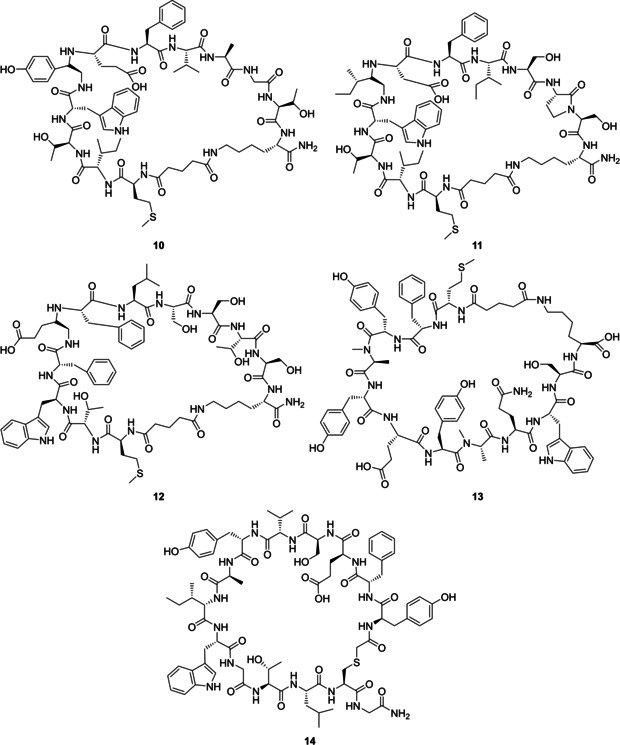
Chemical structures of mRNA display‐derived peptides targeting the Gα accessory protein interface. The peptides cycGiBP (**10**),^[233]^ cycPRP‐1 (**11**), cycPRP‐3 (**12**),^[234]^ and Gα SUPR (**13**)^[235]^ are Gαi1⋅GDP selective. GsNI‐1 (**14**)^[217]^ is Gαs⋅GTP selective.

*mRNA display approach*. Along with using the actual protein motifs to develop modulators, they have also been used as templates for high‐throughput techniques (peptide sequences in Table S1). For example, the Roberts group used a GPR consensus‐derived mRNA display library for the screening against Gαi1⋅GDP and identified the Gαi⋅GDP‐specific R6A and minimized its sequence to the 9mer peptide R6A‐1. Both peptides competed with Gβγ for Gαi1 binding. It was hypothesized that the GDI activity was conserved, however, this was contradicted in later studies for R6A‐1.[^227^, ^228^] R6A‐1 binds to Switch II/α3 of Gαi1 and also showed binding to the other Gα subfamilies in the GDP‐bound state.[^228^, ^229^] Therefore, R6A‐1 was postulated as a core motif for Gα interaction[^227^, ^229^] and was subsequently used for the development of Gαi⋅GDP⋅AlF_4_^−^ binders^[230]^ and Gαs binders within Switch II/α3.^[231]^ The first approach yielded AR6‐05, which competes with Gβγ for Gαi1 binding and favors the GDP‐bound more than the GDP⋅AlF_4_^−^‐bound state.^[230]^ The second approach used a two‐step selection process, identifying two Gαs⋅GDP‐specific peptides (GSP), mGSP‐1 and mGSP‐2, which maintain specific contacts with Switch II/α3 and inhibit the formation of the heterotrimer. It was shown for GSP, mGSP‐1, and mGSP‐2 that they act as GDI for Gαs, with GSP also acting as GEF for Gαi1, thus showing bifunctional GEM‐like properties.^[231]^ Further optimization strategies of R6A‐1 included N‐methylations in order to increase its proteolytic stability.^[232]^ By using an mRNA display with a macrocyclic peptide construct, the proteolytic stability towards chymotrypsin of the identified Gαi⋅GDP‐selective cycGiBP (**10**, Figure 8) was significantly increased compared to its linear variant linGiBP. Both peptides compete with R6A for binding to Gαi1, and therefore an equal binding site was assumed.^[233]^ Subsequently, the library was first digested with chymotrypsin, followed by mRNA display selection against Gαi1⋅GDP, leading to hits with increased chymotrypsin resistance and stability in human plasma.^[234]^ The respective peptides were referred to as cyclic protease resistant peptides (cycPRP‐1 (**11**), cycPRP‐3 (**12**), both Figure 8). Due to the similar core consensus, it was suggested that both peptides also bind to Gαi1 on Switch II/α3.^[234]^ By using an mRNA display containing also unnatural amino acids, the Gαi⋅GDP‐selective SUPR (**13**, scanning unnatural protease resistant, Figure 8) was obtained exhibiting a further improved stability in human serum, a half‐life of ∼900 min in liver microsomes and a 35‐fold better *in vivo* stability in mouse compared to cycGiBP.^[235]^

Recently, in a modified mRNA display approach, the Gαs⋅GTP‐selective GsIN‐1 (**14**, Figure 8) was identified using a Random nonstandard Peptide Integrated Discovery (RaPID) system, which also addresses Switch II/α3 and inhibits Gαs.^[217]^

*Phage display approach*. The first phage display towards Gαi1 was performed with a commercially available peptide library and two peptide families (consensus ΩPXXΩHP (peptide 1) and LPΩXXXH (peptide 3) with Ω: aromatic amino acids) with G protein‐activating properties were identified, however, no structural information was described.^[236]^ In another phage display experiment with Gαi1⋅GDP, the GDP‐selective peptide KB‐752 was discovered showing GEM‐like activity (GEF for Gαi1 and GDI for Gαs) and high similarity to the GEM motif.[^215^, ^237^] In a crystal structure analysis with Gαi⋅GDP, the peptide was shown to bind into the hydrophobic cleft of Switch II/α3 (like the GEM motif of GIV, Section 2.5, Figure S6).^[215]^ Altogether, KB‐752 is able to inhibit cAMP production through its bifunctional function within the G protein‐mediated AC activity, which has been shown in cell membrane preparations.^[237]^ In addition, a consensus to the previously described R6A‐1 ([T/Y/F]‐W‐[WY]‐[ED]‐[FY]‐L) was identified, based on which the Switch II/α3 binding site of R6A‐1 and the subsequently developed mRNA display peptides were concluded.[^228^, ^231^, ^233^] In a second experiment, a phage display was performed with Gαi1⋅GTPγS, resulting in the active‐state selective peptides KB‐1753, KB‐1746, and KB‐1755.[^216^, ^238^] KB‐1753 is capable of inhibiting the interaction of Gαt with its effector cGMP PDEγ and Gαt‐mediated activation of cGMP degradation, as well as interfering with RGS protein binding.[^216^, ^238^] Crystal structure analysis of KB‐1753 in complex with Gαi1⋅GDP⋅AlF_4_^−^ showed that KB‐1753 also binds into a conserved hydrophobic pocket between Switch II and α3.^[216]^ Based on results in competition binding assays, it was shown that the Gαi1 binding sites of KB‐1753 and KB‐1755 as well as of KB‐1755 and KB‐1746 partially overlap, whereas the binding sites of KB‐1753 and KB‐1746 do not. Furthermore, KB‐1755 was shown to interact with Gα the effector and RGS protein binding region. Thus, KB‐1746 was thought to predominantly interact with the RGS binding site of Gα, as KB‐1753 predominantly addresses the effector binding site.[^216^, ^238^]

*OBOC library screening*. In a recent study, using an one‐bead‐one‐compound (OBOC) library screening against Gαi1⋅GDP, we identified a peptide, GPM‐1, with high sequence similarity to KB‐752^[237]^ and the GEM‐motif,[^119^, ^120^] which was further modified to increase cell permeability and proteolytic stability. The optimized peptides exhibited GDI activity towards Gαs and GEF activity towards Gαi1 in a GEM‐like activity. Thus, the peptides may lower the cAMP concentration in the cellular context via the G protein‐mediated AC activity. Using molecular modeling and docking analyses, the peptides were shown to bind to Gαi1⋅GDP similarly to KB‐752 and the GIV‐GEM motif within Switch II/α3. Such compounds may thus be considered valuable tools for the study of G protein‐mediated signal transduction and pathogenesis (unpublished results).

In summary, the peptides described predominantly address the Switch II/α3 region (Figure S9), which appears to be well exposed and well targetable/druggable. This is demonstrated by the fact that this region is not only targeted in directed approaches, but also in non‐directed attempts. The binding cleft between the Switch II α2‐helix and α3 is well accessible within both, Gαi and Gαs, in either state of activity, as shown by the diverse peptides presented in this section. The variation in state selectivity and subfamily specificity is due to the varying conformation of the switch regions, which allows only peptides with certain structural features to bind. Thus, addressing the Switch II/α3 region is an interesting objective for future applications of both, peptides, which allow more selective binding due to larger interaction areas, and small molecules.

## 4 Summary and Outlook

G proteins play a crucial role in signal transduction and in a variety of physiological processes. However, this might also indicate that G proteins are involved in the development and progression of diseases in case of malfunctions in respective signaling cascades. GPCRs are already targeted by over 30 % of the FDA‐approved drugs and are consequently well druggable through their extracellular ligand binding site.[^4^, ^5^] However, targeting G proteins is an attractive alternative compared to GPCR‐directed drugs, for example, in cases of multifactorial diseases, in which multiple GPCRs are involved, or in cases where the disease pathogenesis occurs downstream of the GPCR at the G protein level. To date, no drugs addressing G proteins have been approved or tested in clinical trials, rendering the development of tool compounds crucial for pharmacological research.[^1^, ^2^, ^11^, ^180^]

The Gα subunit of heterotrimeric G proteins has a high potential for manipulation by modulators, because of its various structural determinants and its role as molecular switch. Here, we examined the five different interaction sites of Gαi/s, namely the Gα‐GPCR, the nucleotide binding pocket, the Gα‐Gβγ, the Gα‐effector, and the Gα‐accessory protein interface, in more detail highlighting the structural characteristics of these interactions. Subsequently, all modulators known so far from the literature were assigned to one of these interface regions, and the approach used to identify these modulators was analyzed for its potential to provide an important starting point for targeting these previously “undruggable” proteins in the future.^[14]^

Regarding the Gα‐GPCR interface, many natural compounds are known to address the Gα N‐ and C‐termini, which are thus readily accessible to potential modulators, as evidenced for the N‐terminus by its post‐translational modifications and for the C‐terminus by the ability to develop specific antibodies for this region (Supporting Information). However, the substances targeting this interface also exhibit non‐G protein‐specific activities, which renders them unsuitable for clinical studies and as leads. We consider this interface to be less attractive for modulator development, since the variety of GPCRs with their G protein coupling selectivities only allows to address few specific receptor‐mediated signaling pathway simultaneously.

Targeting the nucleotide binding pocket by modulators is a suitable tool to study G protein signaling and to evaluate novel modulators occupying different interface regions. GNPs are important to induce artificially different activation states and thus distinct Gα conformations, for example within crystal structure analyses. Furthermore, GNPs are valuable in evaluating whether compounds affect the nucleotide exchange, and exhibit GDI, GEF or GEM activity, or alter the GTPase function, which might be achieved by binding of the respective compound to the Gαi/s‐accessory protein interface. Additionally, GNPs are also critical for determining the quality of recombinant G proteins. For modulator development, these compounds are less suitable because they can also target other guanine nucleotide‐binding proteins.

The assignment of modulators to the Gα‐Gβγ and Gα‐effector interface is not trivial, since the interaction regions overlap with the contact areas of accessory proteins, depending on the Gα activation state. Thus, these interface areas have potential for being addressed by tool compounds, although the development starting from the accessory proteins is more promising.

Finally, the Gα‐accessory protein interface might possess the highest potential for modulator design, since accessory proteins themselves influence the Gα activity and can therefore be used as models or lead structures. This is evident from the fact that peptides derived from the GPR or GEM motif can affect the G protein activity *in vitro* or in conjugation with CPPs intracellularly. In addition to directed approaches that aimed to directly address this interface, non‐directed high‐throughput techniques also yielded compounds that were able to address this interface. These compounds were frequently associated with modulator properties. Overall, the analysis of this interface has shown that especially the Switch II/α3 region is well exposed and druggable, which has already been described by DiGiacomo et al.^[188]^ in the context of small molecules, but can further be extended to the peptide level. This region could therefore be approached experimentally on the basis of protein motifs or already identified binders/modulators, or theoretically by directed docking experiments using the above‐described approaches. Comparing the potential of small molecules with that of peptides indicates that peptides show a higher selectivity due to more specific contacts than small molecules. In addition, the identified peptide modulators of the Switch II/α3 region demonstrate that state‐selective or subfamily‐selective modulators can be developed, as the conformation of the Switch II/α3 binding cleft differs accordingly.

As a consequence for future investigations, novel modulators may be identified based on the conformation of the Switch II/α3 region, using especially directed high‐throughput techniques, but also the already identified compounds, which can be further developed as lead structures. At the same time, the approach of identifying natural compounds should be considered as a valuable strategy, although it might be time‐consuming and non‐directed.

In conclusion, Gα proteins have an enormous potential for being targeted by pharmacological tools and drugs. Such compounds would provide a viable alternative to circumvent the necessity of targeting GPCRs in the future, especially in the context of multifactorial diseases or diseases associated with downstream defects of GPCR signaling.

## Conflict of interest

The authors declare no conflict of interest.

## Biographical Information

*Britta Nubbemeyer obtained her B.Sc. (2015) and M.Sc. (2017) in chemistry at the University of Bonn, Germany. Currently, she is a Ph.D. student at the Pharmaceutical Institute in the group of Prof. Dr. Diana Imhof. Her research focuses on the modulation of Gαi/s protein activity by linear and macrocyclic peptides for tool development*.

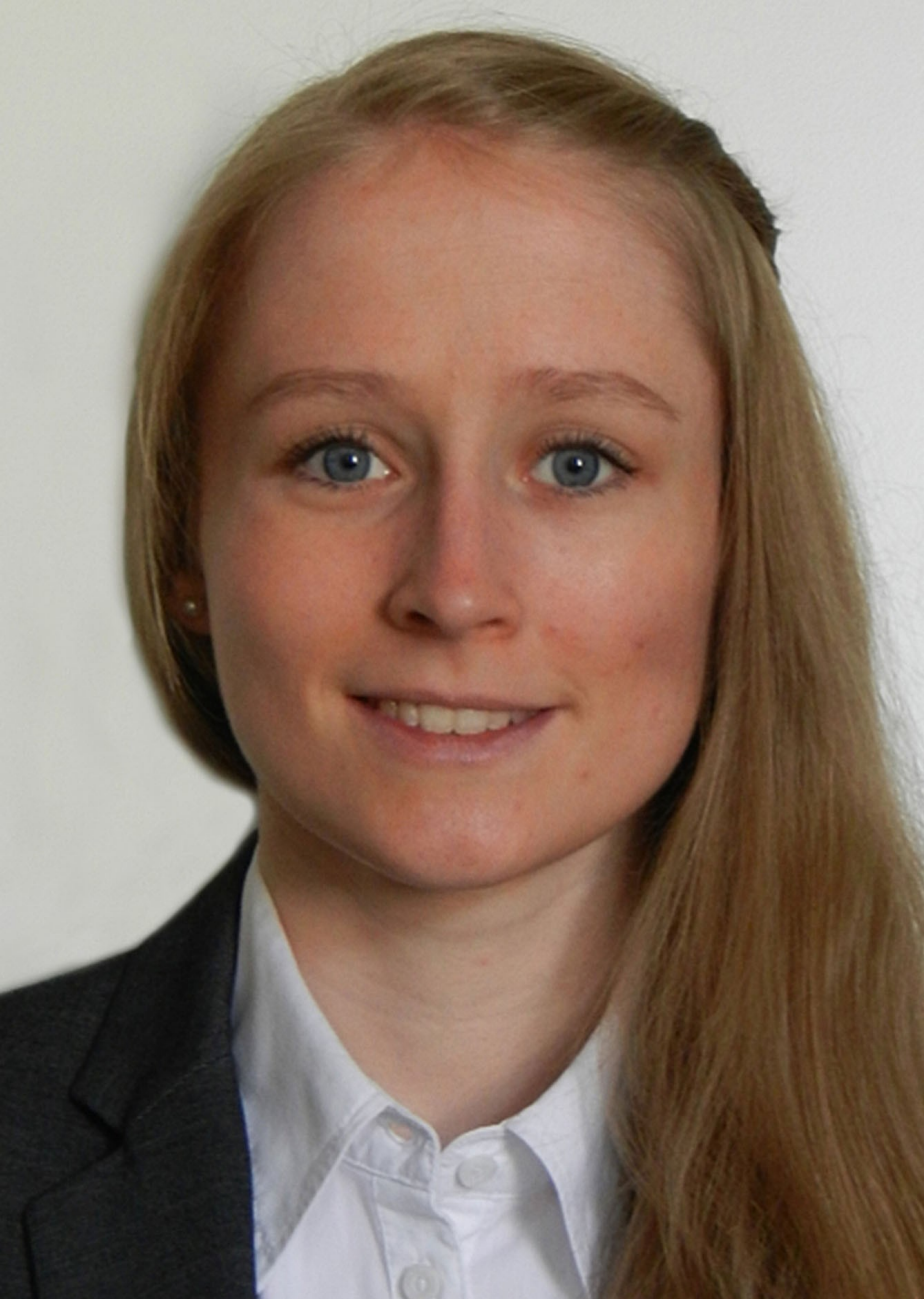

## Biographical Information

*Anna Pepanian received her B.Sc. in chemistry at the Aristotle University of Thessaloniki, Greece in 2017 and her M.Sc. in biochemistry at the University of Bonn in 2019. She continued her studies as a Ph.D. student at the Pharmaceutical Institute of the University of Bonn. Her work focuses on Gα protein expression and investigation of Gα‐peptide/probes interactions for tool development*.

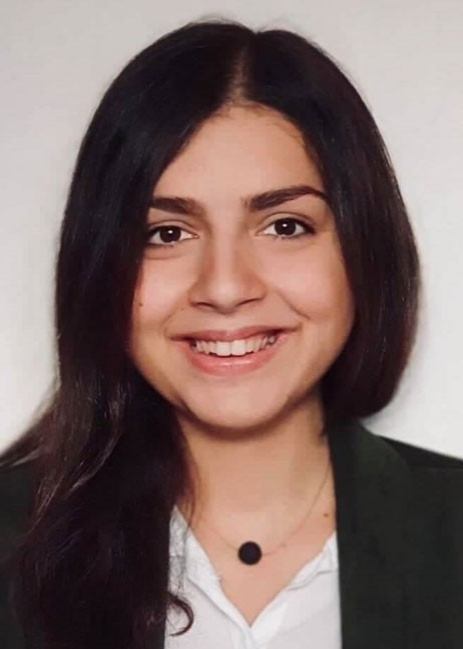

## Biographical Information

*Ajay Abisheck Paul George obtained his B.Tech in biotechnology from VIT University, Vellore India (2009). After a three‐year stint as software developer at Cognizant Technology Solutions, Chennai, India, he moved to Germany for higher education. He obtained both his M.Sc. in life science informatics (2016), and his PhD (2020) from the University of Bonn, Germany. He is currently the Product Owner for Computer‐Aided Drug Design software at BioSolveIT GmbH, St. Augustin, Germany. His work focuses on molecular modeling, computational chemistry and bioinformatics*.

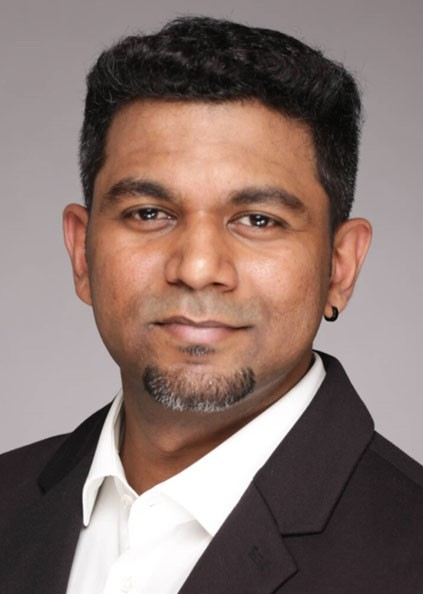

## Biographical Information

*Diana Imhof received her chemistry diploma (1996) and her Ph.D. in biochemistry (1999) at the University of Jena. After her postdoc at the University of Jena and the Ohio State University, she headed a young investigator research group at the Center of Molecular Biomedicine in Jena. She joined the University of Bonn in 2011 as Associate Professor. Since 2016, she has been a Full Professor for Pharmaceutical Biochemistry and Bioanalytics. Her research focuses on bioactive peptides and proteins with therapeutic potential, including peptidic Gα modulators*.

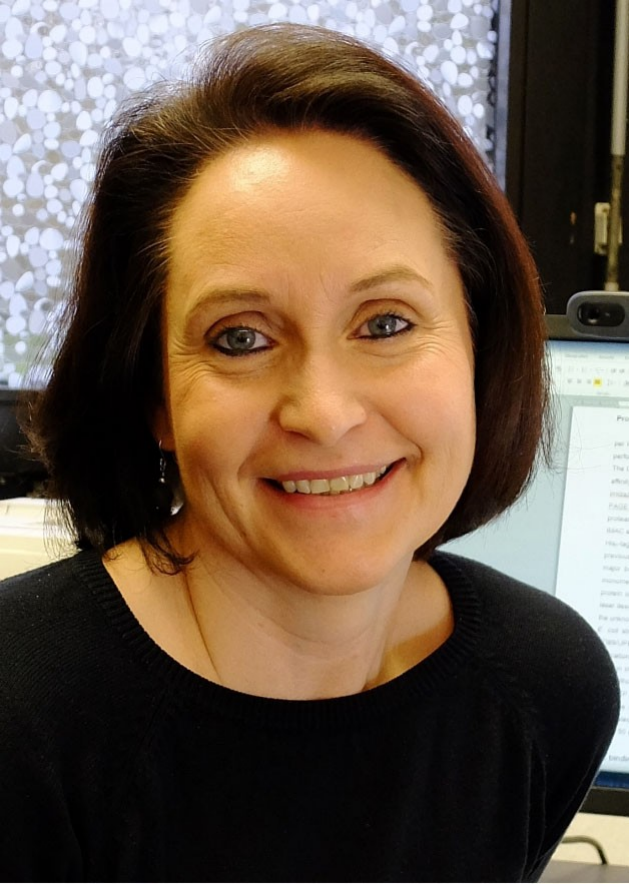

## Supporting information

As a service to our authors and readers, this journal provides supporting information supplied by the authors. Such materials are peer reviewed and may be re‐organized for online delivery, but are not copy‐edited or typeset. Technical support issues arising from supporting information (other than missing files) should be addressed to the authors.

Supplementary

## References

[1] J. Li , Y. Ge , J.-X. Huang , K. Strømgaard , X. Zhang , X.-F. Xiong , J. Med. Chem. 2020, 63, 5013. 31841625 10.1021/acs.jmedchem.9b01452

[2] A. P. Campbell , A. V. Smrcka , Nat. Rev. Drug Discovery 2018, 17, 789. 30262890 10.1038/nrd.2018.135 PMC6409483

[3] V. Syrovatkina , K. O. Alegre , R. Dey , X.-Y. Huang , J. Mol. Biol. 2016, 428, 3850. 27515397 10.1016/j.jmb.2016.08.002 PMC5023507

[4] A. S. Hauser , M. M. Attwood , M. Rask-Andersen , H. B. Schiöth , D. E. Gloriam , Nat. Rev. Drug Discovery 2017, 16, 829. 29075003 10.1038/nrd.2017.178 PMC6882681

[5] K. Sriram , P. A. Insel , Mol. Pharmacol. 2018, 93, 251. 29298813 10.1124/mol.117.111062 PMC5820538

[6] M. Rask-Andersen , S. Masuram , H. B. Schiöth , Annu. Rev. Pharmacol. Toxicol. 2014, 54, 9. 24016212 10.1146/annurev-pharmtox-011613-135943

[7] L. Larribère , J. Utikal , Cancers 2020, 12, 1524. 10.3390/cancers12061524 PMC7352965 32532044

[8] M. O'Hayre , J. Vázquez-Prado , I. Kufareva , E. W. Stawiski , T. M. Handel , S. Seshagiri , J. S. Gutkind , Nat. Rev. Cancer 2013, 13, 412. 23640210 10.1038/nrc3521 PMC4068741

[9] R. Bar-Shavit , M. Maoz , A. Kancharla , J. K. Nag , D. Agranovich , S. Grisaru-Granovsky , B. Uziely , Int. J. Mol. Sci. 2016, 17, 1320. 10.3390/ijms17081320 PMC5000717 27529230

[10] N. Arang , J. S. Gutkind , FEBS Lett. 2020, 24, 4201. 10.1002/1873-3468.14017 PMC8849590 33270228

[11] M. A. Ayoub , Eur. J. Pharmacol. 2018, 826, 169. 29522725 10.1016/j.ejphar.2018.03.003

[12] J. Neumann , H. Scholz , V. Döring , W. Schmitz , L. Meyerinck , P. Kalmár , The Lancet 1988, 332, 936. 10.1016/s0140-6736(88)92601-3 2902384

[13] D. A. Deshpande , R. B. Penn , Cell. Signalling 2006, 18, 2105. 16828259 10.1016/j.cellsig.2006.04.008

[14] C. V. Dang , E. P. Reddy , K. M. Shokat , L. Soucek , Nat. Rev. Cancer 2017, 17, 502. 28643779 10.1038/nrc.2017.36 PMC5945194

[15] R. Santos , O. Ursu , A. Gaulton , A. P. Bento , R. S. Donadi , C. G. Bologa , A. Karlsson , B. Al-Lazikani , A. Hersey , T. I. Oprea , et al., Nat. Rev. Drug Discovery 2017, 16, 19. 27910877 10.1038/nrd.2016.230 PMC6314433

[16] W. M. Oldham , H. E. Hamm , Q. Rev. Biophys. 2006, 39, 117. 16923326 10.1017/S0033583506004306

[17] W. M. Oldham , H. E. Hamm , Nat. Rev. Mol. Cell Biol. 2008, 9, 60. 18043707 10.1038/nrm2299

[18] G. B. Downes , N. Gautam , Genomics 1999, 62, 544. 10644457 10.1006/geno.1999.5992

[19] A. G. Gilman , Biosci. Rep. 1995, 15, 65. 7579036 10.1007/BF01200143

[20] M. Rodbell , Biosci. Rep. 1995, 15, 117. 7579038 10.1007/BF01207453

[21] G. Milligan , E. Kostenis , Br. J. Pharmacol. 2006, 147 Suppl 1, S46–55. 16402120 10.1038/sj.bjp.0706405 PMC1760735

[22] A.-L. Schmitz , R. Schrage , E. Gaffal , T. H. Charpentier , J. Wiest , G. Hiltensperger , J. Morschel , S. Hennen , D. Häußler , V. Horn , et al., Chem. Biol. 2014, 21, 890. 25036778 10.1016/j.chembiol.2014.06.003 PMC4337399

[23] I. S. Moreira , P. A. Fernandes , M. J. Ramos , Proteins 2007, 68, 803. 17546660 10.1002/prot.21396

[24] J. Wang , Y. Miao , Adv. Protein Chem. Struct. Biol. 2019, 116, 397. 31036298 10.1016/bs.apcsb.2018.11.011 PMC6986689

[25] Z. Qian , P. G. Dougherty , D. Pei , Curr. Opin. Chem. Biol. 2017, 38, 80. 28388463 10.1016/j.cbpa.2017.03.011 PMC5474178

[26] P. G. Dougherty , Z. Qian , D. Pei , Biochem. J. 2017, 474, 1109. 28298556 10.1042/BCJ20160619 PMC6511976

[27] H. Derakhshankhah , S. Jafari , Biomed. Pharmacother. 2018, 108, 1090. 30372809 10.1016/j.biopha.2018.09.097

[28] T. B. Trinh , P. Upadhyaya , Z. Qian , D. Pei , ACS Comb. Sci. 2016, 18, 75. 26645887 10.1021/acscombsci.5b00164 PMC4710893

[29] P. Upadhyaya , Z. Qian , N. G. Selner , S. R. Clippinger , Z. Wu , R. Briesewitz , D. Pei , Angew. Chem. Int. Ed. 2015, 54, 7602. 10.1002/anie.201502763 PMC4591930 25950772

[30] P. G. Dougherty , A. Sahni , D. Pei , Chem. Rev. 2019, 119, 10241. 31083977 10.1021/acs.chemrev.9b00008 PMC6739158

[31] T. Passioura , Biochemistry 2020, 59, 139. 31592645 10.1021/acs.biochem.9b00802

[32] Z. Qian , P. Upadhyaya , D. Pei , in Methods in Molecular Biology (Ed.: R. Derda ), Springer, New York, 2015, pp. 39–53. 10.1007/978-1-4939-2020-4_3 25616324

[33] S. S. Sidhu , W. J. Fairbrother , K. Deshayes , ChemBioChem 2003, 4, 14. 12512072 10.1002/cbic.200390008

[34] T. T. Takahashi , R. W. Roberts , Methods Mol. Biol. 2009, 535, 293. 19377989 10.1007/978-1-59745-557-2_17

[35] S. Henrich , O. M. H. Salo-Ahen , B. Huang , F. F. Rippmann , G. Cruciani , R. C. Wade , J. Mol. Recognit. 2009, 209–219. 10.1002/jmr.984 19746440

[36] H. Zhang , A. L. Nielsen , K. Strømgaard , Med. Res. Rev. 2020, 1, 135. 10.1002/med.21598 31218731

[37] S. Annala , X. Feng , N. Shridhar , F. Eryilmaz , J. Patt , J. Yang , E. M. Pfeil , R. D. Cervantes-Villagrana , A. Inoue , F. Häberlein , et al., Sci. Signaling 2019, 12, eaau5948. 10.1126/scisignal.aau5948 30890659

[38] M. Matthey , R. Roberts , A. Seidinger , A. Simon , R. Schröder , M. Kuschak , S. Annala , G. M. König , C. E. Müller , I. P. Hall , et al., Sci. Transl. Med. 2017, 9, 1. 10.1126/scitranslmed.aag2288 28904224

[39] R. Schrage , A.-L. Schmitz , E. Gaffal , S. Annala , S. Kehraus , D. Wenzel , K. M. Büllesbach , T. Bald , A. Inoue , Y. Shinjo , et al., Nat. Commun. 2015, 6, 10156. 26658454 10.1038/ncomms10156 PMC4682109

[40] X.-F. Xiong , H. Zhang , C. R. Underwood , K. Harpsøe , T. J. Gardella , M. F. Wöldike , M. Mannstadt , D. E. Gloriam , H. Bräuner-Osborne , K. Strømgaard , Nat. Chem. 2016, 8, 1. 27768111 10.1038/nchem.2577 PMC5559716

[41] R. Reher , T. Kühl , S. Annala , T. Benkel , D. Kaufmann , B. Nubbemeyer , J. P. Odhiambo , P. Heimer , C. A. Bäuml , S. Kehraus , et al., ChemMedChem 2018, 13, 1634. 29873888 10.1002/cmdc.201800304

[42] C. Draper-Joyce , S. G. B. Furness , ACS Pharmacol. Transl. Sci. 2019, 2, 285. 32259062 10.1021/acsptsci.9b00054 PMC7088962

[43] J. B. Blumer , S. M. Lanier , Mol. Pharmacol. 2014, 85, 388. 24302560 10.1124/mol.113.090068 PMC3935153

[44] A. J. Kimple , D. E. Bosch , P. M. Giguère , D. P. Siderovski , Pharmacol. Rev. 2011, 63, 728. 21737532 10.1124/pr.110.003038 PMC3141876

[45] D. P. Siderovski , F. S. Willard , Int. J. Biol. Sci. 2005, 1, 51. 15951850 10.7150/ijbs.1.51 PMC1142213

[46] P. Ghosh , P. Rangamani , I. Kufareva , Cell Cycle 2017, 16, 607. 28287365 10.1080/15384101.2017.1282584 PMC5397260

[47] E. M. Ross , T. M. Wilkie , Annu. Rev. Biochem. 2000, 69, 795. 10966476 10.1146/annurev.biochem.69.1.795

[48] N. Wettschureck , S. Offermanns , Physiol. Rev. 2005, 85, 1159. 16183910 10.1152/physrev.00003.2005

[49] S. R. Sprang , Z. Chen , X. Du , Adv. Protein Chem. 2007, 74, 1. 17854654 10.1016/S0065-3233(07)74001-9

[50] I. S. Moreira , Biochim. Biophys. Acta 2014, 1840, 16. 24016604 10.1016/j.bbagen.2013.08.027

[51] J. P. Noel , H. E. Hamm , P. B. Sigler , Nature 1993, 366, 654. 8259210 10.1038/366654a0

[52] D. G. Lambright , J. P. Noel , H. E. Hamm , P. B. Sigler , Nature 1994, 369, 621. 8208289 10.1038/369621a0

[53] F. A. Baltoumas , M. C. Theodoropoulou , S. J. Hamodrakas , J. Struct. Biol. 2013, 182, 209. 23523730 10.1016/j.jsb.2013.03.004

[54] N. A. Lambert , C. A. Johnston , S. D. Cappell , S. Kuravi , A. J. Kimple , F. S. Willard , D. P. Siderovski , Proc. Natl. Acad. Sci. USA 2010, 107, 7066. 20351284 10.1073/pnas.0912934107 PMC2872438

[55] D. Goricanec , R. Stehle , P. Egloff , S. Grigoriu , A. Plückthun , G. Wagner , F. Hagn , Proc. Natl. Acad. Sci. USA 2016, 113, E3629–38. 27298341 10.1073/pnas.1604125113 PMC4932968

[56] R. J. Lefkowitz , Angew. Chem. Int. Ed. 2013, 52, 6366. 10.1002/anie.201301924 23650015

[57] B. Kobilka , Angew. Chem. Int. Ed. 2013, 52, 6380. 10.1002/anie.201302116 PMC4031317 23650120

[58] D. Hilger , M. Masureel , B. K. Kobilka , Nat. Struct. Mol. Biol. 2018, 25, 4. 29323277 10.1038/s41594-017-0011-7 PMC6535338

[59] A. Glukhova , C. J. Draper-Joyce , R. K. Sunahara , A. Christopoulos , D. Wootten , P. M. Sexton , ACS Pharmacol. Transl. Sci. 2018, 1, 73. 32219204 10.1021/acsptsci.8b00026 PMC7089011

[60] Y. Kang , O. Kuybeda , P. W. de Waal , S. Mukherjee , N. van Eps , P. Dutka , X. E. Zhou , A. Bartesaghi , S. Erramilli , T. Morizumi , et al., Nature 2018, 558, 553. 29899450 10.1038/s41586-018-0215-y PMC8054211

[61] C.-J. Tsai , J. Marino , R. Adaixo , F. Pamula , J. Muehle , S. Maeda , T. Flock , N. M. Taylor , I. Mohammed , H. Matile , et al., eLife 2019, 8, e46041. 31251171 10.7554/eLife.46041 PMC6629373

[62] J. Wang , T. Hua , Z.-J. Liu , Curr. Opin. Struct. Biol. 2020, 63, 82. 32485565 10.1016/j.sbi.2020.04.008

[63] X. E. Zhou , K. Melcher , H. E. Xu , Protein Sci. 2019, 28, 487. 30311978 10.1002/pro.3526 PMC6371222

[64] M. Sandhu , A. M. Touma , M. Dysthe , F. Sadler , S. Sivaramakrishnan , N. Vaidehi , Proc. Natl. Acad. Sci. USA 2019, 116, 11956. 31138704 10.1073/pnas.1820944116 PMC6575595

[65] X. Liu , X. Xu , D. Hilger , P. Aschauer , J. K. S. Tiemann , Y. Du , H. Liu , K. Hirata , X. Sun , R. Guixà-González , et al., Cell 2019, 177, 1243–1251.e12. 31080070 10.1016/j.cell.2019.04.021 PMC6991123

[66] T. Flock , A. S. Hauser , N. Lund , D. E. Gloriam , S. Balaji , M. M. Babu , Nature 2017, 545, 317. 28489817 10.1038/nature22070 PMC5846738

[67] T. Flock , C. N. J. Ravarani , D. Sun , A. J. Venkatakrishnan , M. Kayikci , C. G. Tate , D. B. Veprintsev , M. M. Babu , Nature 2015, 524, 173. 26147082 10.1038/nature14663 PMC4866443

[68] J. Sondek , D. G. Lambright , J. P. Noel , H. E. Hamm , P. B. Sigler , Nature 1994, 372, 276. 7969474 10.1038/372276a0

[69] S. Rens-Domiano , H. E. Hamm , FASEB J. 1995, 9, 1059. 7649405 10.1096/fasebj.9.11.7649405

[70] D. E. Coleman , S. R. Sprang , Biochemistry 1998, 37, 14376. 9772163 10.1021/bi9810306

[71] H. R. Bourne , D. A. Sanders , F. McCormick , Nature 1990, 348, 125. 2122258 10.1038/348125a0

[72] J. P. Mahoney , R. K. Sunahara , Curr. Opin. Struct. Biol. 2016, 41, 247.27871057

[73] M. B. Mixon , E. Lee , D. E. Coleman , A. M. Berghuis , A. G. Gilman , S. R. Sprang , Science 1995, 270, 954. 7481799 10.1126/science.270.5238.954

[74] R. O. Dror , T. J. Mildorf , D. Hilger , A. Manglik , D. W. Borhani , D. H. Arlow , A. Philippsen , N. Villanueva , Z. Yang , M. T. Lerch , et al., Science 2015, 348, 1361. 26089515 10.1126/science.aaa5264 PMC4968074

[75] X.-Q. Yao , R. U. Malik , N. W. Griggs , L. Skjærven , J. R. Traynor , S. Sivaramakrishnan , B. J. Grant , J. Biol. Chem. 2016, 291, 4742. 26703464 10.1074/jbc.M115.702605 PMC4813496

[76] X. Sun , S. Singh , K. J. Blumer , G. R. Bowman , eLife 2018, 7. 10.7554/eLife.38465 PMC6224197 30289386

[77] N. van Eps , A. M. Preininger , N. Alexander , A. I. Kaya , S. Meier , J. Meiler , H. E. Hamm , W. L. Hubbell , Proc. Natl. Acad. Sci. USA 2011, 108, 9420. 21606326 10.1073/pnas.1105810108 PMC3111277

[78] S. Maeda , A. Koehl , H. Matile , H. Hu , D. Hilger , G. F. X. Schertler , A. Manglik , G. Skiniotis , R. J. P. Dawson , B. K. Kobilka , Nat. Commun. 2018, 9, 3712. 30213947 10.1038/s41467-018-06002-w PMC6137068

[79] A. Koehl , H. Hu , S. Maeda , Y. Zhang , Q. Qu , J. M. Paggi , N. R. Latorraca , D. Hilger , R. Dawson , H. Matile , et al., Nature 2018, 558, 547. 29899455 10.1038/s41586-018-0219-7 PMC6317904

[80] D. Sun , T. Flock , X. Deupi , S. Maeda , M. Matkovic , S. Mendieta , D. Mayer , R. Dawson , G. F. X. Schertler , M. Madan Babu , et al., Nat. Struct. Mol. Biol. 2015, 22, 686. 26258638 10.1038/nsmb.3070 PMC4876908

[81] H. E. Kato , Y. Zhang , H. Hu , C.-M. Suomivuori , F. M. N. Kadji , J. Aoki , K. Krishna Kumar , R. Fonseca , D. Hilger , W. Huang , et al., Nature 2019, 572, 80. 31243364 10.1038/s41586-019-1337-6 PMC7065593

[82] S. Majumdar , S. Ramachandran , R. A. Cerione , J. Biol. Chem. 2006, 281, 9219. 16469737 10.1074/jbc.M513837200

[83] A. M. Berghuis , E. Lee , A. S. Raw , A. G. Gilman , S. R. Sprang , Structure 1996, 4, 1277. 8939752 10.1016/s0969-2126(96)00136-0

[84] A. I. de Opakua , K. Parag-Sharma , V. DiGiacomo , N. Merino , A. Leyme , A. Marivin , M. Villate , L. T. Nguyen , M. A. de La Cruz-Morcillo , J. B. Blanco-Canosa , et al., Nat. Commun. 2017, 8, 15163. 28516903 10.1038/ncomms15163 PMC5454376

[85] A. V. Smrcka , I. Fisher , Cell. Mol. Life Sci. 2019, 76, 4447. 31435698 10.1007/s00018-019-03275-2 PMC6842434

[86] M. A. Wall , D. E. Coleman , E. Lee , J. A. Iñiguez-Lluhi , B. A. Posner , A. G. Gilman , S. R. Sprang , Cell 1995, 83, 1047. 8521505 10.1016/0092-8674(95)90220-1

[87] D. G. Lambright , J. Sondek , A. Bohm , N. P. Skiba , H. E. Hamm , P. B. Sigler , Nature 1996, 379, 311. 8552184 10.1038/379311a0

[88] J. Sondek , A. Bohm , D. G. Lambright , H. E. Hamm , P. B. Sigler , Nature 1996, 379, 369. 8552196 10.1038/379369a0

[89] M. E. Linder , I. H. Pang , R. J. Duronio , J. I. Gordon , P. C. Sternweis , A. G. Gilman , J. Biol. Chem. 1991, 266, 4654. 1900297

[90] R. K. Sunahara , J. J. Tesmer , A. G. Gilman , S. R. Sprang , Science 1997, 278, 1943. 9395396 10.1126/science.278.5345.1943

[91] K. C. Slep , M. A. Kercher , W. He , C. W. Cowan , T. G. Wensel , P. B. Sigler , Nature 2001, 409, 1071. 11234020 10.1038/35059138

[92] C. Qi , S. Sorrentino , O. Medalia , V. M. Korkhov , Science 2019, 364, 389. 31023924 10.1126/science.aav0778

[93] J. J. Tesmer , R. K. Sunahara , A. G. Gilman , S. R. Sprang , Science 1997, 278, 1907. 9417641 10.1126/science.278.5345.1907

[94] G. Grishina , C. H. Berlot , J. Biol. Chem. 1997, 272, 20619. 9252377 10.1074/jbc.272.33.20619

[95] W. J. Tang , J. H. Hurley , Mol. Pharmacol. 1998, 54, 231. 9687563 10.1124/mol.54.2.231

[96] R. K. Sunahara , C. W. Dessauer , R. E. Whisnant , C. Kleuss , A. G. Gilman , J. Biol. Chem. 1997, 272, 22265. 9268375 10.1074/jbc.272.35.22265

[97] S. C. van Keulen , D. Narzi , U. Rothlisberger , Biochemistry 2019, 58, 4317. 31525953 10.1021/acs.biochem.9b00662

[98] C. W. Dessauer , J. J. Tesmer , S. R. Sprang , A. G. Gilman , J. Biol. Chem. 1998, 273, 25831. 9748257 10.1074/jbc.273.40.25831

[99] S. C. van Keulen , U. Rothlisberger , PLoS Comput. Biol. 2017, 13, e1005673. 28892485 10.1371/journal.pcbi.1005673 PMC5608429

[100] J. E. Grant , L.-W. Guo , M. M. Vestling , K. A. Martemyanov , V. Y. Arshavsky , A. E. Ruoho , J. Biol. Chem. 2006, 281, 6194. 16407279 10.1074/jbc.M509511200

[101] J. B. Blumer , S. S. Oner , S. M. Lanier , Acta Physiol. 2012, 204, 202. 10.1111/j.1748-1716.2011.02327.x 21615707

[102] M. Natochin , K. G. Gasimov , N. O. Artemyev , Biochemistry 2001, 40, 5322. 11318657 10.1021/bi015505w

[103] B. Sjögren , Adv. Pharmacol. 2011, 62, 315. 21907914 10.1016/B978-0-12-385952-5.00002-6

[104] K. C. Slep , M. A. Kercher , T. Wieland , C.-K. Chen , M. I. Simon , P. B. Sigler , Proc. Natl. Acad. Sci. USA 2008, 105, 6243. 18434540 10.1073/pnas.0801569105 PMC2359805

[105] A. Takesono , M. J. Cismowski , C. Ribas , M. Bernard , P. Chung , S. Hazard , E. Duzic , S. M. Lanier , J. Biol. Chem. 1999, 274, 33202. 10559191 10.1074/jbc.274.47.33202

[106] Y. K. Peterson , S. Hazard , S. G. Graber , S. M. Lanier , J. Biol. Chem. 2002, 277, 6767. 11756403 10.1074/jbc.C100699200

[107] D. P. Siderovski , M. A. Diversé-Pierluissi , L. de Vries , Trends Biochem. Sci. 1999, 24, 340. 10470031 10.1016/s0968-0004(99)01441-3

[108] F. S. Willard , R. J. Kimple , D. P. Siderovski , Annu. Rev. Biochem. 2004, 73, 925. 15189163 10.1146/annurev.biochem.73.011303.073756

[109] R. J. Kimple , M. E. Kimple , L. Betts , J. Sondek , D. P. Siderovski , Nature 2002, 416, 878. 11976690 10.1038/416878a

[110] F. S. Willard , Z. Zheng , J. Guo , G. J. Digby , A. J. Kimple , J. M. Conley , C. A. Johnston , D. Bosch , M. D. Willard , V. J. Watts , et al., J. Biol. Chem. 2008, 283, 36698. 18984596 10.1074/jbc.M804936200 PMC2605979

[111] K. Khafizov , J. Mol. Model. 2009, 15, 1491. 19437048 10.1007/s00894-009-0516-z PMC2847169

[112] C. Liu , J. Weng , D. Wang , M. Yang , M. Jia , W. Wang , Biochemistry 2018, 57, 6562. 30406994 10.1021/acs.biochem.8b00848

[113] L. J. McClelland , K. Zhang , T.-C. Mou , J. Johnston , C. Yates-Hansen , S. Li , C. J. Thomas , T. I. Doukov , S. Triest , A. Wohlkonig , et al., Nat. Commun. 2020, 11, 1077. 32103024 10.1038/s41467-020-14943-4 PMC7044438

[114] M. M. Papasergi , B. R. Patel , G. G. Tall , Mol. Pharmacol. 2015, 87, 52. 25319541 10.1124/mol.114.094664 PMC4279082

[115] N. van Eps , C. J. Thomas , W. L. Hubbell , S. R. Sprang , Proc. Natl. Acad. Sci. USA 2015, 112, 1404. 25605908 10.1073/pnas.1423878112 PMC4321267

[116] A. B. Seven , D. Hilger , M. M. Papasergi-Scott , L. Zhang , Q. Qu , B. K. Kobilka , G. G. Tall , G. Skiniotis , Cell Rep. 2020, 30, 3699–3709.e6. 32126208 10.1016/j.celrep.2020.02.086 PMC7192526

[117] D. Srivastava , L. Gakhar , N. O. Artemyev , Nat. Commun. 2019, 10, 3084. 31300652 10.1038/s41467-019-11088-x PMC6625990

[118] D. Srivastava , N. O. Artemyev , J. Biol. Chem. 2019, 294, 17875. 31624147 10.1074/jbc.AC119.011135 PMC6879328

[119] V. Gupta , D. Bhandari , A. Leyme , N. Aznar , K. K. Midde , I.-C. Lo , J. Ear , I. Niesman , I. López-Sánchez , J. B. Blanco-Canosa , et al., Proc. Natl. Acad. Sci. USA 2016, 113, E5721–30. 27621449 10.1073/pnas.1609502113 PMC5047194

[120] V. DiGiacomo , A. Marivin , M. Garcia-Marcos , Biochemistry 2018, 57, 255. 29035513 10.1021/acs.biochem.7b00845 PMC6082369

[121] N. A. Kalogriopoulos , S. D. Rees , T. Ngo , N. J. Kopcho , A. V. Ilatovskiy , N. Sun , E. A. Komives , G. Chang , P. Ghosh , I. Kufareva , Proc. Natl. Acad. Sci. USA 2019, 116, 16394. 31363053 10.1073/pnas.1906658116 PMC6697900

[122] J. J. Tesmer , D. M. Berman , A. G. Gilman , S. R. Sprang , Cell 1997, 89, 251. 9108480 10.1016/s0092-8674(00)80204-4

[123] S. P. Srinivasa , N. Watson , M. C. Overton , K. J. Blumer , J. Biol. Chem. 1998, 273, 1529. 9430692 10.1074/jbc.273.3.1529

[124] M. Soundararajan , F. S. Willard , A. J. Kimple , A. P. Turnbull , L. J. Ball , G. A. Schoch , C. Gileadi , O. Y. Fedorov , E. F. Dowler , V. A. Higman , et al., Proc. Natl. Acad. Sci. USA 2008, 105, 6457. 18434541 10.1073/pnas.0801508105 PMC2359823

[125] A. J. Kimple , M. Soundararajan , S. Q. Hutsell , A. K. Roos , D. J. Urban , V. Setola , B. R. S. Temple , B. L. Roth , S. Knapp , F. S. Willard , et al., J. Biol. Chem. 2009, 284, 19402. 19478087 10.1074/jbc.M109.024711 PMC2740565

[126] A. Asli , I. Sadiya , M. Avital-Shacham , M. Kosloff , Sci. Signaling 2018, 11. 10.1126/scisignal.aan3677 29895615

[127] M. E. Sowa , W. He , T. G. Wensel , O. Lichtarge , Proc. Natl. Acad. Sci. USA 2000, 97, 1483. 10677488 10.1073/pnas.030409597 PMC26460

[128] P. E. Stein , A. Boodhoo , G. D. Armstrong , S. A. Cockle , M. H. Klein , R. J. Read , Structure 1994, 2, 45. 8075982 10.1016/s0969-2126(00)00007-1

[129] S. Mangmool , H. Kurose , Toxins 2011, 3, 884. 22069745 10.3390/toxins3070884 PMC3202852

[130] T. Katada , M. Ui , Proc. Natl. Acad. Sci. USA 1982, 79, 3129. 6954463 10.1073/pnas.79.10.3129 PMC346367

[131] T. Higashijima , S. Uzu , T. Nakajima , E. M. Ross , J. Biol. Chem. 1988, 263, 6491. 3129426

[132] M. Mousli , C. Bronner , J.-L. Bueb , Y. Landry , Eur. J. Pharmacol. 1991, 207, 249. 1716580 10.1016/0922-4106(91)90037-i

[133] H. Kusunoki , K. Wakamatsu , K. Sato , T. Miyazawa , T. Kohno , Biochemistry 1998, 37, 4782. 9537994 10.1021/bi972756p

[134] M. Mousli , J.-L. Bueb , C. Bronner , B. Rouot , Y. Landry , Trends Pharmacol. Sci. 1990, 11, 358. 2122563 10.1016/0165-6147(90)90179-c

[135] M. P. Dos Santos Cabrera , M. Rangel , J. Ruggiero Neto , K. Konno , Toxins 2019, 11, 559. 10.3390/toxins11100559 PMC6832458 31554187

[136] T. Higashijima , J. Burnier , E. M. Ross , J. Biol. Chem. 1990, 265, 14176. 2117607

[137] H. S. Kachel , S. D. Buckingham , D. B. Sattelle , Curr. Opin. Insect Sci. 2018, 30, 93. 30553492 10.1016/j.cois.2018.10.001

[138] L. N. Irazazabal , W. F. Porto , S. M. Ribeiro , S. Casale , V. Humblot , A. Ladram , O. L. Franco , Biochim. Biophys. Acta 2016, 1858, 2699. 27423268 10.1016/j.bbamem.2016.07.001

[139] N. Fukushima , M. Kohno , T. Kato , S. Kawamoto , K. Okuda , Y. Misu , H. Ueda , Peptides 1998, 19, 811. 9663445 10.1016/s0196-9781(98)00027-8

[140] M. Sukumar , E. M. Ross , T. Higashijima , Biochemistry 1997, 36, 3632. 9132015 10.1021/bi962356m

[141] U. Tomita , K. Takahashi , K. Ikenaka , T. Kondo , I. Fujimoto , S. Aimoto , K. Mikoshiba , M. Ui , T. Katada , Biochem. Biophys. Res. Commun. 1991, 178, 400. 1906273 10.1016/0006-291x(91)91827-y

[142] M. Mousli , C. Bronner , J. Bockaert , B. Rouot , Y. Landry , Immunol. Lett. 1990, 25, 355. 1701162 10.1016/0165-2478(90)90207-7

[143] T. Higashijima , E. M. Ross , J. Biol. Chem. 1991, 266, 12655. 1905730

[144] M. Sukumar , T. Higashijima , J. Biol. Chem. 1992, 267, 21421. 1400455

[145] R. Weingarten , L. Ransnäs , H. Mueller , L. A. Sklar , G. M. Bokoch , J. Biol. Chem. 1990, 265, 11044. 2113529

[146] A. V. R. da Silva , B. M. De Souza , M. P. dos Santos Cabrera , N. B. Dias , P. C. Gomes , J. R. Neto , R. G. Stabeli , M. S. Palma , Biochim. Biophys. Acta 2014, 1838, 2357. 24955498 10.1016/j.bbamem.2014.06.012

[147] C. Oppi , T. Wagner , A. Crisari , B. Camerini , G. P. Tocchini Valentini , Proc. Natl. Acad. Sci. USA 1992, 89, 8268. 1518856 10.1073/pnas.89.17.8268 PMC49899

[148] J. Silva , V. Monge-Fuentes , F. Gomes , K. Lopes , L. dos Anjos , G. Campos , C. Arenas , A. Biolchi , J. Gonçalves , P. Galante , et al., Toxins 2015, 7, 3179. 26295258 10.3390/toxins7083179 PMC4549745

[149] T. C. Terwilliger , D. Eisenberg , J. Biol. Chem. 1982, 257, 6016. 7076662

[150] M. Mousli , J. L. Bueb , B. Rouot , Y. Landry , C. Bronner , Agents Actions 1991, 33, 81. 1716843 10.1007/BF01993132

[151] M. Mousli , C. Bronner , Y. Landry , J. Bockaert , B. Rouot , FEBS Lett. 1990, 259, 260. 1688415 10.1016/0014-5793(90)80023-c

[152] H. Mukai , E. Munekata , T. Higashijima , J. Biol. Chem. 1992, 267, 16237. 1379592

[153] I. Fujimoto , K. Ikenaka , T. Kondo , S. Aimoto , M. Kuno , K. Mikoshiba , FEBS Lett. 1991, 287, 15. 1908786 10.1016/0014-5793(91)80005-n

[154] A. Chahdi , L. Daeffler , J. P. Gies , Y. Landry , Fundam. Clin. Pharmacol. 1998, 12, 121. 9565765 10.1111/j.1472-8206.1998.tb00932.x

[155] A. Hagelüken , B. Nürnberg , R. Harhammer , L. Grünbaum , W. Schunack , R. Seifert , Mol. Pharmacol. 1995, 47, 234. 7870030

[156] A. Hagelüken , L. Grünbaum , B. Nürnberg , R. Harhammer , W. ScHunack , R. Seifert , Biochem. Pharmacol. 1994, 47, 1789. 7911302 10.1016/0006-2952(94)90307-7

[157] E. Breitweg-Lehmann , C. Czupalla , R. Storm , O. Kudlacek , W. ScHunack , M. Freissmuth , B. Nürnberg , Mol. Pharmacol. 2002, 61, 628. 11854444 10.1124/mol.61.3.628

[158] I. Fatima , S. Kanwal , T. Mahmood , Dose-Response 2019, 17, 1559325818813227. 30670935 10.1177/1559325818813227 PMC6328957

[159] G. Gajski , A.-M. Domijan , B. Žegura , A. Štern , M. Gerić , I. Novak Jovanović , I. Vrhovac , J. Madunić , D. Breljak , M. Filipič , et al., Toxicon 2016, 110, 56. 26704293 10.1016/j.toxicon.2015.12.005

[160] M. Moreno , E. Giralt , Toxins 2015, 7, 1126. 25835385 10.3390/toxins7041126 PMC4417959

[161] B. M. de Souza , M. P. D. S. Cabrera , P. C. Gomes , N. B. Dias , R. G. Stabeli , N. B. Leite , J. R. Neto , M. S. Palma , Peptides 2015, 72, 164. 25944744 10.1016/j.peptides.2015.04.021

[162] N. B. Leite , L. C. da Costa , D. Dos Santos Alvares , M. P. Dos Santos Cabrera , B. M. de Souza , M. S. Palma , J. Ruggiero Neto , Amino Acids 2011, 40, 91. 20195659 10.1007/s00726-010-0511-9

[163] C. Höller , M. Freissmuth , C. Nanoff , Cell. Mol. Life Sci. 1999, 55, 257. 10188585 10.1007/s000180050288 PMC11147085

[164] J. M. Taylor , R. R. Neubig , Cell. Signalling 1994, 6, 841. 7718404 10.1016/0898-6568(94)90017-5

[165] A. O. Shpakov , J. Amino Acids 2011, 2011, 656051. 22312467 10.4061/2011/656051 PMC3268021

[166] W. W. Ja , R. W. Roberts , Trends Biochem. Sci. 2005, 30, 318. 15950876 10.1016/j.tibs.2005.04.001

[167] G. M. Bokoch , T. Katada , J. K. Northup , E. L. Hewlett , A. G. Gilman , J. Biol. Chem. 1983, 258, 2072. 6296122

[168] I. Jarmoskaite , I. AlSadhan , P. P. Vaidyanathan , D. Herschlag , eLife 2020, 9, e57264. 32758356 10.7554/eLife.57264 PMC7452723

[169] A. E. Remmers , R. R. Neubig , J. Biol. Chem. 1996, 271, 4791. 8617747 10.1074/jbc.271.9.4791

[170] D. P. McEwen , K. R. Gee , H. C. Kang , R. R. Neubig , Anal. Biochem. 2001, 291, 109. 11262163 10.1006/abio.2001.5011

[171] Y. Li , P. M. Sternweis , S. Charnecki , T. F. Smith , A. G. Gilman , E. J. Neer , T. Kozasa , J. Biol. Chem. 1998, 273, 16265. 9632686 10.1074/jbc.273.26.16265

[172] T. M. Bonacci , M. Ghosh , S. Malik , A. V. Smrcka , J. Biol. Chem. 2005, 280, 10174. 15611108 10.1074/jbc.M412514200

[173] M. L. Bernard , Y. K. Peterson , P. Chung , J. Jourdan , S. M. Lanier , J. Biol. Chem. 2001, 276, 1585. 11042168 10.1074/jbc.M005291200

[174] A. V. Smrcka , Cell. Mol. Life Sci. 2008, 65, 2191. 18488142 10.1007/s00018-008-8006-5 PMC2688713

[175] O. Vögler , J. M. Barceló , C. Ribas , P. V. Escribá , Biochim. Biophys. Acta 2008, 1778, 1640. 18402765 10.1016/j.bbamem.2008.03.008

[176] W. Tang , Y. Tu , S. K. Nayak , J. Woodson , M. Jehl , E. M. Ross , J. Biol. Chem. 2006, 281, 4746. 16407201 10.1074/jbc.M510573200

[177] J. A. Iñiguez-Lluhi , M. I. Simon , J. D. Robishaw , A. G. Gilman , J. Biol. Chem. 1992, 267, 23409. 1429682

[178] O. Kisselev , M. Ermolaeva , N. Gautam , J. Biol. Chem. 1995, 270, 25356. 7592699 10.1074/jbc.270.43.25356

[179] N. Gautam , G. B. Downes , K. Yan , O. Kisselev , Cell. Signalling 1998, 10, 447. 9754712 10.1016/s0898-6568(98)00006-0

[180] A. V. Smrcka , Trends Pharmacol. Sci. 2013, 34, 290. 23557963 10.1016/j.tips.2013.02.006 PMC3924570

[181] M. Hohenegger , M. Waldhoer , W. Beindl , B. Böing , A. Kreimeyer , P. Nickel , C. Nanoff , M. Freissmuth , Proc. Natl. Acad. Sci. USA 1998, 95, 346. 9419378 10.1073/pnas.95.1.346 PMC18220

[182] J. Wang , P. Sengupta , Y. Guo , U. Golebiewska , S. Scarlata , J. Biol. Chem. 2009, 284, 16906. 19369247 10.1074/jbc.M109.006585 PMC2719327

[183] W.-C. Chung , J. C. Kermode , J. Pharmacol. Exp. Ther. 2005, 313, 191. 15626724 10.1124/jpet.104.078311

[184] M. Freissmuth , M. Waldhoer , E. Bofill-Cardona , C. Nanoff , Trends Pharmacol. Sci. 1999, 20, 237. 10366866 10.1016/s0165-6147(99)01337-1

[185] W. Beindl , T. Mitterauer , M. Hohenegger , A. P. IJzerman , C. Nanoff , M. Freissmuth , Mol. Pharmacol. 1996, 50, 415. 8700151

[186] M. Freissmuth , S. Boehm , W. Beindl , P. Nickel , A. P. IJzerman , M. Hohenegger , C. Nanoff , Mol. Pharmacol. 1996, 49, 602. 8609887

[187] S. J. Butler , E. C. Kelly , F. R. McKenzie , S. B. Guild , M. J. Wakelam , G. Milligan , Biochem. J. 1988, 251, 201. 2839158 10.1042/bj2510201 PMC1148984

[188] V. DiGiacomo , A. I. de Opakua , M. P. Papakonstantinou , L. T. Nguyen , N. Merino , J. B. Blanco-Canosa , F. J. Blanco , M. Garcia-Marcos , Sci. Rep. 2017, 7, 8575. 28819150 10.1038/s41598-017-08829-7 PMC5561080

[189] A. V. Smrcka , D. M. Lehmann , A. L. Dessal , Comb. Chem. High Throughput Screening 2008, 11, 382. 10.2174/138620708784534761 PMC2688719 18537559

[190] J. Hanoune , N. Defer , Annu. Rev. Pharmacol. Toxicol. 2001, 41, 145. 11264454 10.1146/annurev.pharmtox.41.1.145

[191] C. R. McCudden , M. D. Hains , R. J. Kimple , D. P. Siderovski , F. S. Willard , Cell. Mol. Life Sci. 2005, 62, 551. 15747061 10.1007/s00018-004-4462-3 PMC2794341

[192] M. Natochin , A. E. Granovsky , N. O. Artemyev , J. Biol. Chem. 1998, 273, 21808. 9705319 10.1074/jbc.273.34.21808

[193] J. K. Northup , P. C. Sternweis , M. D. Smigel , L. S. Schleifer , E. M. Ross , A. G. Gilman , Proc. Natl. Acad. Sci. USA 1980, 77, 6516. 6935665 10.1073/pnas.77.11.6516 PMC350316

[194] B. Yoo , R. Iyengar , Y. Chen , J. Biol. Chem. 2004, 279, 13925. 14722077 10.1074/jbc.M314334200

[195] D. L. Roman , J. R. Traynor , J. Med. Chem. 2011, 54, 7433. 21916427 10.1021/jm101572n PMC3208131

[196] J. B. O'Brien , J. C. Wilkinson , D. L. Roman , J. Biol. Chem. 2019, 294, 18571. 31636120 10.1074/jbc.REV119.007060 PMC6901330

[197] M. Salaga , M. Storr , K. A. Martemyanov , J. Fichna , BioEssays 2016, 38, 344. 26817719 10.1002/bies.201500118 PMC4916644

[198] K. Aktories , Nat. Rev. Microbiol. 2011, 9, 487. 21677684 10.1038/nrmicro2592

[199] C. Merlen , D. Fayol-Messaoudi , S. Fabrega , T. El Hage , A. Servin , F. Authier , FEBS J. 2005, 272, 4385. 16128808 10.1111/j.1742-4658.2005.04851.x

[200] D. M. Gill , R. Meren , Proc. Natl. Acad. Sci. USA 1978, 75, 3050. 210449 10.1073/pnas.75.7.3050 PMC392711

[201] L. de Haan , T. R. Hirst , Mol. Membr. Biol. 2004, 21, 77. 15204437 10.1080/09687680410001663267

[202] D. Cassel , T. Pfeuffer , Proc. Natl. Acad. Sci. USA 1978, 75, 2669. 208069 10.1073/pnas.75.6.2669 PMC392624

[203] L. Birnbaumer , M. Birnbaumer , J. Recept. Signal Transduction Res. 1995, 15, 213. 10.3109/10799899509045218 8903942

[204] M. Freissmuth , A. G. Gilman , J. Biol. Chem. 1989, 264, 21907. 2557345

[205] C. van Dop , M. Tsubokawa , H. R. Bourne , J. Ramachandran , J. Biol. Chem. 1984, 259, 696. 6582062

[206] T. K. Sixma , S. E. Pronk , K. H. Kalk , E. S. Wartna , B. A. van Zanten , B. Witholt , W. G. Hol , Nature 1991, 351, 371. 2034287 10.1038/351371a0

[207] T. K. Sixma , K. H. Kalk , B. A. van Zanten , Z. Dauter , J. Kingma , B. Witholt , W. G. Hol , J. Mol. Biol. 1993, 230, 890. 8478941 10.1006/jmbi.1993.1209

[208] I. Preuss , D. Hildebrand , J. H. C. Orth , K. Aktories , K. F. Kubatzky , Cell. Microbiol. 2010, 12, 1174. 20331638 10.1111/j.1462-5822.2010.01462.x

[209] J. H. C. Orth , I. Preuss , I. Fester , A. Schlosser , B. A. Wilson , K. Aktories , Proc. Natl. Acad. Sci. USA 2009, 106, 7179. 19369209 10.1073/pnas.0900160106 PMC2678484

[210] J. H. C. Orth , I. Fester , P. Siegert , M. Weise , U. Lanner , S. Kamitani , T. Tachibana , B. A. Wilson , A. Schlosser , Y. Horiguchi , et al., FASEB J. 2013, 27, 832. 23150526 10.1096/fj.12-213900 PMC3545528

[211] B. A. Wilson , M. Ho , Future Microbiol. 2010, 5, 1185. 20722598 10.2217/fmb.10.91 PMC4407829

[212] T. Jank , X. Bogdanović , C. Wirth , E. Haaf , M. Spoerner , K. E. Böhmer , M. Steinemann , J. H. C. Orth , H. R. Kalbitzer , B. Warscheid , et al., Nat. Struct. Mol. Biol. 2013, 20, 1273. 24141704 10.1038/nsmb.2688

[213] R. G. Zhang , D. L. Scott , M. L. Westbrook , S. Nance , B. D. Spangler , G. G. Shipley , E. M. Westbrook , J. Mol. Biol. 1995, 251, 563. 7658473 10.1006/jmbi.1995.0456

[214] K. Kitadokoro , S. Kamitani , M. Miyazawa , M. Hanajima-Ozawa , A. Fukui , M. Miyake , Y. Horiguchi , Proc. Natl. Acad. Sci. USA 2007, 104, 5139. 17360394 10.1073/pnas.0608197104 PMC1829276

[215] C. A. Johnston , F. S. Willard , M. R. Jezyk , Z. Fredericks , E. T. Bodor , M. B. Jones , R. Blaesius , V. J. Watts , T. K. Harden , J. Sondek , et al., Structure 2005, 13, 1069. 16004878 10.1016/j.str.2005.04.007 PMC1405235

[216] C. A. Johnston , E. S. Lobanova , A. S. Shavkunov , J. Low , J. K. Ramer , R. Blaesius , Z. Fredericks , F. S. Willard , B. Kuhlman , V. Y. Arshavsky , et al., Biochemistry 2006, 45, 11390. 16981699 10.1021/bi0613832 PMC2597383

[217] S. A. Dai , Q. Hu , R. Gao , A. Lazar , Z. Zhang , M. von Zastrow , H. Suga , K. M. Shokat , bioRixv preprint, 2020, DOI: 10.1101/2020.04.25.054080.

[218] G. P. Prévost , M. O. Lonchampt , S. Holbeck , S. Attoub , D. Zaharevitz , M. Alley , J. Wright , M. C. Brezak , H. Coulomb , A. Savola , et al., Cancer Res. 2006, 66, 9227. 16982767 10.1158/0008-5472.CAN-05-4205

[219] C. Favre-Guilmard , H. Zeroual-Hider , C. Soulard , C. Touvay , P.-E. Chabrier , G. Prevost , M. Auguet , Eur. J. Pharmacol. 2008, 594, 70. 18664366 10.1016/j.ejphar.2008.07.016

[220] M. A. Ayoub , M. Damian , C. Gespach , E. Ferrandis , O. Lavergne , O. de Wever , J.-L. Banères , J.-P. Pin , G. P. Prévost , J. Biol. Chem. 2009, 284, 29136. 19648112 10.1074/jbc.M109.042333 PMC2781458

[221] J. Küppers , T. Benkel , S. Annala , G. Schnakenburg , E. Kostenis , M. Gütschow , MedChemComm 2019, 10, 1838. 32180917 10.1039/c9md00269c PMC7053702

[222] J. Küppers , T. Benkel , S. Annala , K. Kimura , L. Reinelt , B. K. Fleischmann , E. Kostenis , M. Gütschow , Chemistry. 2020, 26, 12615. 32428383 10.1002/chem.202001446 PMC7590114

[223] K. M. Appleton , K. J. Bigham , C. C. Lindsey , S. Hazard , J. Lirjoni , S. Parnham , M. Hennig , Y. K. Peterson , Bioorg. Med. Chem. 2014, 22, 3423. 24818958 10.1016/j.bmc.2014.04.035 PMC4103618

[224] Y. K. Peterson , M. L. Bernard , H. Ma , S. Hazard , S. G. Graber , S. M. Lanier , J. Biol. Chem. 2000, 275, 33193. 10969064 10.1074/jbc.C000509200

[225] C. R. McCudden , F. S. Willard , R. J. Kimple , C. A. Johnston , M. D. Hains , M. B. Jones , D. P. Siderovski , Biochim. Biophys. Acta 2005, 1745, 254. 15946753 10.1016/j.bbamcr.2005.05.002

[226] G. S. Ma , N. Aznar , N. Kalogriopoulos , K. K. Midde , I. Lopez-Sanchez , E. Sato , Y. Dunkel , R. L. Gallo , P. Ghosh , Proc. Natl. Acad. Sci. USA 2015, 112, E2602–10. 25926659 10.1073/pnas.1505543112 PMC4443320

[227] W. W. Ja , R. W. Roberts , Biochemistry 2004, 43, 9265. 15248784 10.1021/bi0498398

[228] F. S. Willard , D. P. Siderovski , Biochem. Biophys. Res. Commun. 2006, 339, 1107. 16338227 10.1016/j.bbrc.2005.11.132

[229] W. W. Ja , A. Adhikari , R. J. Austin , S. R. Sprang , R. W. Roberts , J. Biol. Chem. 2005, 280, 32057. 16051611 10.1074/jbc.C500319200

[230] W. W. Ja , O. Wiser , R. J. Austin , L. Y. Jan , R. W. Roberts , ACS Chem. Biol. 2006, 1, 570. 17168552 10.1021/cb600345k PMC2802464

[231] R. J. Austin , W. W. Ja , R. W. Roberts , J. Mol. Biol. 2008, 377, 1406. 18329041 10.1016/j.jmb.2008.01.032 PMC2891084

[232] S. V. Fiacco , R. W. Roberts , ChemBioChem 2008, 9, 2200. 18780385 10.1002/cbic.200800208 PMC2962921

[233] S. W. Millward , S. Fiacco , R. J. Austin , R. W. Roberts , ACS Chem. Biol. 2007, 2, 625. 17894440 10.1021/cb7001126 PMC3747972

[234] S. M. Howell , S. V. Fiacco , T. T. Takahashi , F. Jalali-Yazdi , S. W. Millward , B. Hu , P. Wang , R. W. Roberts , Sci. Rep. 2014, 4, 6008. 25234472 10.1038/srep06008 PMC4168267

[235] S. V. Fiacco , L. E. Kelderhouse , A. Hardy , Y. Peleg , B. Hu , A. Ornelas , P. Yang , S. T. Gammon , S. M. Howell , P. Wang , et al., ChemBioChem 2016, 17, 1643. 27465925 10.1002/cbic.201600253 PMC5167532

[236] J. Hessling , M. J. Lohse , K.-N. Klotz , Biochem. Pharmacol. 2003, 65, 961. 12623127 10.1016/s0006-2952(02)01653-2

[237] C. A. Johnston , J. K. Ramer , R. Blaesius , Z. Fredericks , V. J. Watts , D. P. Siderovski , FEBS Lett. 2005, 579, 5746. 16225870 10.1016/j.febslet.2005.09.059 PMC1363735

[238] C. A. Johnston , F. S. Willard , J. K. Ramer , R. Blaesius , C. N. Roques , D. P. Siderovski , Comb. Chem. High Throughput Screening 2008, 11, 370. 10.2174/138620708784534798 PMC2440659 18537558

