# Supplementary material for: Strategies towards Targeting Gαi/s Proteins: Scanning of Protein‐Protein Interaction Sites To Overcome Inaccessibility
Source: ChemMedChem. 2021 Mar 22;16(11):1697–716. doi: 10.1002/cmdc.202100039 (PMC8252600; doi:10.1002/cmdc.202100039)
Supplement: Supplementary file 1 — Supplementary [file CMDC-16-1697-s001.pdf]

ChemMedChem

Supporting Information

# Strategies towards Targeting Gαi/s Proteins: Scanning of Protein-Protein Interaction Sites To Overcome Inaccessibility

Britta Nubbemeyer, Anna Pepanian, Ajay Abisheck Paul George, and Diana Imhof*

**Table of Contents**

Further information regarding the Gαi/s-nucleotide interactions ................................................2
Antibodies as tools for the investigation of G protein-mediated signal transduction...................3
Supporting Figures..........................................................................................................4
Supporting Tables..........................................................................................................13
References....................................................................................................................14
Author Contributions ....................................................................................................14

## Further information regarding the Gαi/s-nucleotide interactions

Since the early ‘80s, the activation of Gαs[1,2] and Gαi[3] had been studied via binding assays with labelled guanine nucleotide analogs (GNPs), concluding that Gαi subunits are more prominent to bind GNPs compared to Gαs. The most important guanine nucleotide moieties are illustrated in Figure S7. In 1983, Bokoch et al.[2] observed in an incubation experiment with the photoaffinity probe 8-azido-GTP that the highly conserved guanine nucleotide binding site of Gi is located on the Gαi subunit. Furthermore, the nonhydrolyzable compounds GTPγS (**15**, Figure S7), [$^{35}$S]GTPγS, Gpp(NH)p (guanyl-5’-(β,γ-imino)triphosphate, **16**, Figure S7), and GMP (guanosine monophosphate) have been used for testing the binding affinity between these analogs and the Gα nucleotide binding pocket.[3] GTPγS exhibits the highest binding affinity, while GTP, GDP, and Gpp(NH)p are following with relatively equal binding potency, whereas GMP turned out as the weakest binding ligand. This rank order can be structurally explained based on the features of the five motifs as stated in chapter 2.2 (Figure 3), e.g., the P-loop and the RXXTXGI motif contacts, which stabilize the nucleotide phosphate(s) and, explain the preference of GTPγS over GMP. GTPγS is able to directly activate various G proteins such as Gi and Gs in depolarized Chinese hamster lung fibroblasts (CCL39) irreversibly, and cannot be replaced by other nucleotides with the exception of GDPβS (5’-*O*-(2-thiodiphosphate)), which resembles GTPγS in its activity. Thus, GTPγS and GTP analogs can permanently activate G proteins.[4] The presence of $Mg^{2+}$ raises the affinity for GTPγS due to the involvement of the DXXG motif within the Switch II region (active state) beside the RXXTXGI motif of Switch I (active and inactive state) in binding. This effect was accompanied by an increased fluorescence intensity due to the intrinsic tryptophan fluorescence enhancement, a phenomenon which was assumed to indicate the active conformation of the Gα protein, since the inactive Gα protein exhibit a less highly fluorescent state.[5,6] As a matter of fact, GNPs bind with higher affinity to Gαi than to Gαs at low $Mg^{2+}$ concentration and the metal lowers the nucleotide dissociation rate from the respective Gα subunit.[3,7] An analogous fluorescence increase was observed after addition of $F^-$, the quantitative amount of which was diminished by the presence of $Al^{3+}$ ions.[5] This increase could be interpreted by the property of GDP·$AlF_4^-$ to mimic the G protein transition state of the GTPase reaction.[8] Furthermore, tetrahedral $Li^+$ attenuates the formation of Gαi1·GDP·$ALF_4^-$ and thereby decreases the intrinsic fluorescence, due to Gαi1 inactivation because of structural differences between the octahedral coordinated $Mg^{2+}$.[9]
The following decade was a stepping stone to the general study of GNPs which remain fundamental tools for many researchers until today. Two more guanine nucleotide analogs, oGTP (2’,3’-dialdehyde analog of GTP, **18**)[10,11] and oGTPγS (**19**, both structures depicted in Figure S7)[11] were exploited to modulate signaling cascades by competing with GTPγS.[10] The G protein inactivation or activation by oGTP and oGTPγS, respectively, occurs within the guanine nucleotide binding pocket. oGTP forms a Schiff’s base and confines, irreversibly, the G protein in an inactive state, whereas oGTPγS stimulates the Gα subunit after reduction of the Schiff base. In a similar pattern, the binding of the photoaffinity probe [α-$^{35}$P]GTP azidoanilide to Gαs and Gαi is inhibited by oGTP. In the same time period, Remmers et al.[12,13] examined the G protein activation states and their respective conformations after applying N-methyl-3’-*O*-antranoyl (MANT and/or m-, **21**) fluorescent probe on different GNPs. The probe-derivatives, mGpp(NH)p (**16**) and mGpp($CH_2$)p (**17**) activate Gαo proteins by 50%, whereas mGTPγS, mGTP, and mGDP displayed the highest fluorescence (in the respective order mentioned) without completely activating the Gαo protein.[12,13] Afterwards, McEwen et al.[14] exploited the aforementioned results of MANT applications and explored the versatile fluorescent probe BODIPY (4,4-difluoro-4-bora-3a,4a-diaza-s-indance, **20**, Figure S7)[15], to monitor the nucleotide binding in real time. Regarding this, BODIPY FL GTPγS, BODIPY 515 GTPγS, BODIPY TR GTPγS, BODIPY FL Gpp(NH)p, and BODIPY FL GTP analogs are excellent tools both for guanine nucleotide exchange and G protein quantification, binding with high affinity and more specifically to Gαo and Gαs subclasses. However, it seems that BODIPY FL GTPγS is the best binder for the Gαi1/2 subunits. Compared to MANT-analogs, they emit higher fluorescence without requiring energy transfer. Their iodide-quenching suggested that upon binding to the Gα subunit, the BODIPY dye is displayed on the external environment and not in the nucleotide binding pocket. Recently, titration assays have been performed successfully to quantify the percentage of active protein binding to the corresponding ligands. This highlights the involvement of GNPs in G protein activity determination.[16]
Although the guanine nucleotide exchange mechanism has been thoroughly examined over many decades, a deceleration in attempts to discover further probes modulating the foresaid interface with the Gα protein subunit has been noticed. The increasing availability of fluorescently labeled GNPs allows the replacement of radioactively labeled derivatives. Compounds such as BODIPY and GTPγS derivatives need to be the trailhead for more rounds of *in vitro* experiments for high-throughput screenings and evaluation of G protein activity and binding affinities to subsequently engage in signaling pathway regulation. In the context of Gα protein modulators, GNPs are not specific and selective because of their ability to address other GTPases, too. However, GNPs proofed to be efficient for various *in vitro* applications and for X-ray structure determination.[17]

## Antibodies as tools for the investigation of G protein-mediated signal transduction

Alternative strategies for targeting Gαi/s proteins include the involvement of antibodies. Since the late 1980's, several monoclonal antibodies (mAbs) affecting the interaction of G proteins with GPCRs have been discovered.[18] It has been proven that the C-terminal of G proteins contains the critical region for the interaction with the receptor (chapter 2.1, Figure S3).[18–20] An outstanding paradigm is the investigation of the interaction of Gα proteins with the photoreceptor rhodopsin.[18] First attempts resulted in efficient cross-reactivity of the CW6 antiserum towards Gαi/o/t/s subunits, however with partial homology to transducin.[21] The generated mAbs 4A and 7A-D attenuate the interaction between the α-subunits of Gs and Gi, respectively, with the receptor. The binding between the antibody and the corresponding Gα protein subunit occurs because of the homology between the antigenic sites of these five antibodies and the site of interaction of the corresponding Gα protein with the G protein photoreceptor (84% homology for Gαi and 54% for Gαs). Since the monoclonal antibodies associate with the Gα subunits, development of synthetic analogues such as oligopeptides homologous to the respective Gαs and Gαi epitope regions could possibly yield similarly active compounds.[18] More specifically, all these monoclonal antibodies were shown to be bound to the same G protein-derived peptides (C-terminal 5-kDa peptides) near the tryptic digestion site (within Arg310$^{G.h4b6.8}$ and Lys329$^{G.H5.5}$).[19,21] The mAb 4A also attains the ability to sterically impede the connection of PTX to Cys347$^{G.H5.23}$ (chapter 3.1.1) and the corresponding ADP-ribosylation, since its antigenic site is in close proximity to the C-terminus of the associated G protein.[22]

Further experiments have been performed to explore the role of other antibodies in the interaction between the Gαi/o subunit and δ-opioid receptors, which are implicated in Gα protein-effector signaling pathways such as in AC inhibition (Gαi) and calcium channel modulation (Gαo).[20,23] In the interaction of δ-opioid receptors and G proteins, PTX plays a significant role (chapter 3.1.1), since it modifies Cys347$^{G.H5.23}$, and thereby reduces the binding between the protein and the receptor. Affinity-purified antibodies and/or anti-peptide antiserum (anti-Gαi1,2) were produced targeting the Gα C-terminal-derived decapeptides (RM: RMHLRQYELL (RM antisera), QN: QNNLKYIGLC (QN antisera), KE: KENLKDCGLF (AS antisera) and EC: KNNLKECGLY (EC antisera)) to dissociate the receptor from the respective G protein and eventually inhibit the adenylate cyclase activity.[20,24,25] Therefore, it can be concluded that the interaction site between PTX and the Gi-C terminus is the site of choice for *in situ*-antibody interaction.[19,24]

The aforementioned immunogenic C-terminal decapeptides gave the antisera RM, AS and EC, which were reported to develop antibodies explicitly for Gαs, Gαi1/2 and Gαi3 respectively.[24,26] It was shown that upon agonist binding prior to antibody attachment, the GPCR-G protein complex is stabilized and therefore the antibody response is less efficient. Additionally, three further antibodies, i.e. 8A5, 8D11, and 6F12, were found to be selective towards Gαi in the active state conformation. In 2008, Lane et al.[27] noticed that addition of GTPγS seemed to sequester receptor-activated G protein, whereas agonist-activated Gαi2 was recognized by conformationally selective antibodies. Moreover, according to clinical trials[28], the application of antibodies, such as the anti Gα IgGs for the Gαi2 subunits, proved useful in alleviating not only the analgesic effect of the opioid, but also the frequency of withdrawal syndrome. The common denominator of the aforementioned antibodies is the implication of the PTX-sensitive Gαi in the effects of opioid agonists.

Over time, the relationship between mastoparan (chapter 3.1.1) and G proteins was studied by utilizing synthetic polyclonal antibodies (e.g., R16,17) able to hinder the stimulation of GTPase activity that is mediated by mastoparan up to 85%.[29] Mastoparan showed to inhibit the R16,17 antibody binding to both the native and ribosylated Gαi protein subunit, with an affinity reduced by 4 for the latter form.[29,30] As stated in chapter 3.1.1, this toxin resembles agonist-complexed receptors. This could possibly be the reason of the broad administration of antibodies targeting C termini among mastoparan and Gαi proteins.

During the last decade, most experimental studies focused on the utilization of nanobodies, i.e. small and soluble antibodies lacking the light chains that are derived from camelids or llamas (Figure S10).[31–35] Although monoclonal and polyclonal antibodies have been widely described as efficient tools in targeting G protein subunits, their production in large scale is a high-cost procedure. Attempts to preserve the association of Gαs-β2AR complex via antibodies were found to be unsuccessful. Later on, it was found that screening of nanobodies, like Nb35 and Nb37, could overcome the limitations occurred from antibodies' treatment. These nanobodies bind at the interface between Gβ and Gαs-Ras and manage to block the complex dissociation that is usually provoked by the presence of GTPγS (Figure S10).[31,32]

Moreover, Nb5 nanobody was the first discovered nanobody[34] that did not affect the Gαs-mediated cAMP production, although it partially inhibits the Gβγ-signaling pathway, such as the GIRK channel activation, by creating the Gα and the Gβ1γ1 complex (Figure S10). In spite of these remarkable points, it is not always simple to produce them. Nb35 was discovered for Gαs whereas for Gαi1, a unique antibody, called mAb16, was found to bind the interface between Gαi1 and Gβγ subunits. Both molecules stabilize the GPCR/Gα complex and resist the GTPγS-mediated dissociation.[33]

All in all, this section described how several synthesized antibodies, either monoclonal antibodies or nanobodies, were efficient towards targeting both Gαi and Gαs protein subunits by altering the PTX and/or mastoparan activities. Nanobodies are very strong candidates for therapeutic purposes, such as cancer or several neurological pathologies because of their significant pharmaceutical features, like their ability to modulate cellular signaling, induce conformational exchange and mimic effector like proteins.[35] The fact that the C-terminus is the site of GPCR-Gα protein contact, in combination with the exceptional advantages on recent antibody-based and especially, nanobody-implemented studies, could suggest that expansion of the existing knowledge, for instance by providing a broader library of immunogenic molecules, would significantly answer several unresolved questions. Eventually, these molecules could be handled as potent pharmacological tools.

## Supporting Figures

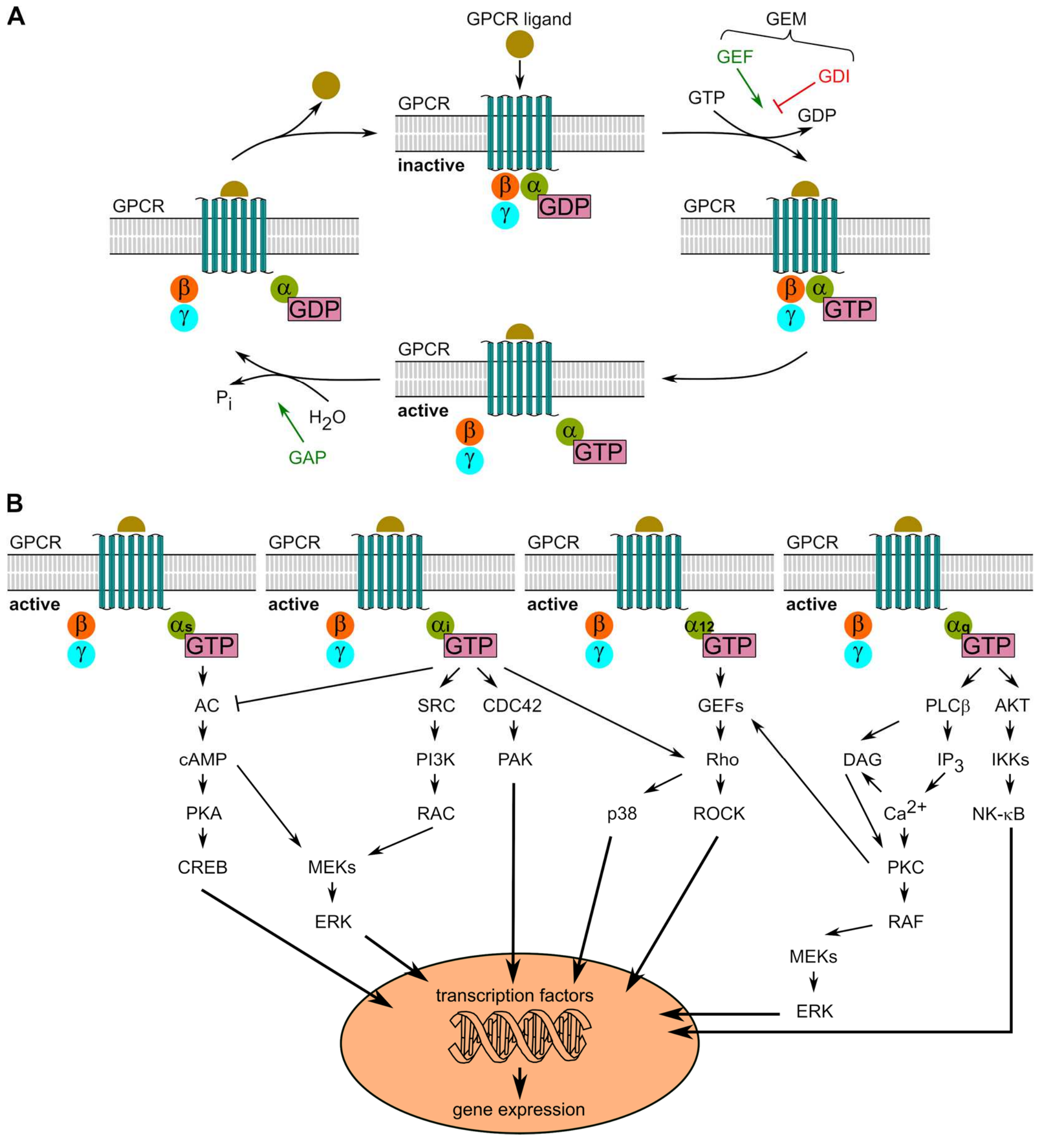


**Figure S1. Signal transduction of heterotrimeric G proteins. A**: Activation cycle of a heterotrimeric G protein: In the inactive state, the GDP-bound G protein is arranged as a heterotrimer. Ligand binding to the GPCR promotes GDP to GTP exchange, which can be accelerated by GEFs (guanine nucleotide exchange factors) or inhibited by GDIs (guanine nucleotide dissociation inhibitors). The GTP-bound heterotrimer dissociates into Gα·GTP and Gβγ (active state), which can influence downstream effectors. After GTP hydrolysis, mediated by the intrinsic GTPase function and accelerated by GAPs (GTPase accelerating proteins), Gα·GDP reassociates with Gβγ. **B**: Selected downstream effector signaling pathways derived from Lappano et al.[36]. Gαs stimulates adenylyl cyclase (AC) and increases cAMP levels, thereby promoting activation of protein kinase A (PKA) and ERK-dependent (ERK: extracellular signal-regulated kinase) signaling. Gαi inhibits AC and activates SRC, PI3K (phosphoinositide 3-kinase), RAC-MEK-ERK (RAC: RAS-related c3 botulinum toxin substrate 1, MEK: mitogen-activated protein kinase kinase) and CDC42-PAK (cell division cycle 42, p21-activated kinase) and Rho-mediated signals. Gα12/13 activates RhoGEFs and thus ROCK (Rho-associated coil-coil containing protein kinase) and p38. Gαq activates phospholipase Cβ (PLCβ), which cleaves phosphatidylinositol-4,5-bisphosphate ($PIP_2$) into diacylglycerol (DAG) and inositol-1,3-5-triphosphate ($IP_3$), leading to $Ca^{2+}$ mobilization, protein kinase C (PKC) activation and thus RhoGEFs and MAPK (mitogen-activated protein kinase) signaling. Gαq also activates the AKT (protein kinase B) pathway, which modulates the NF-κB signaling.

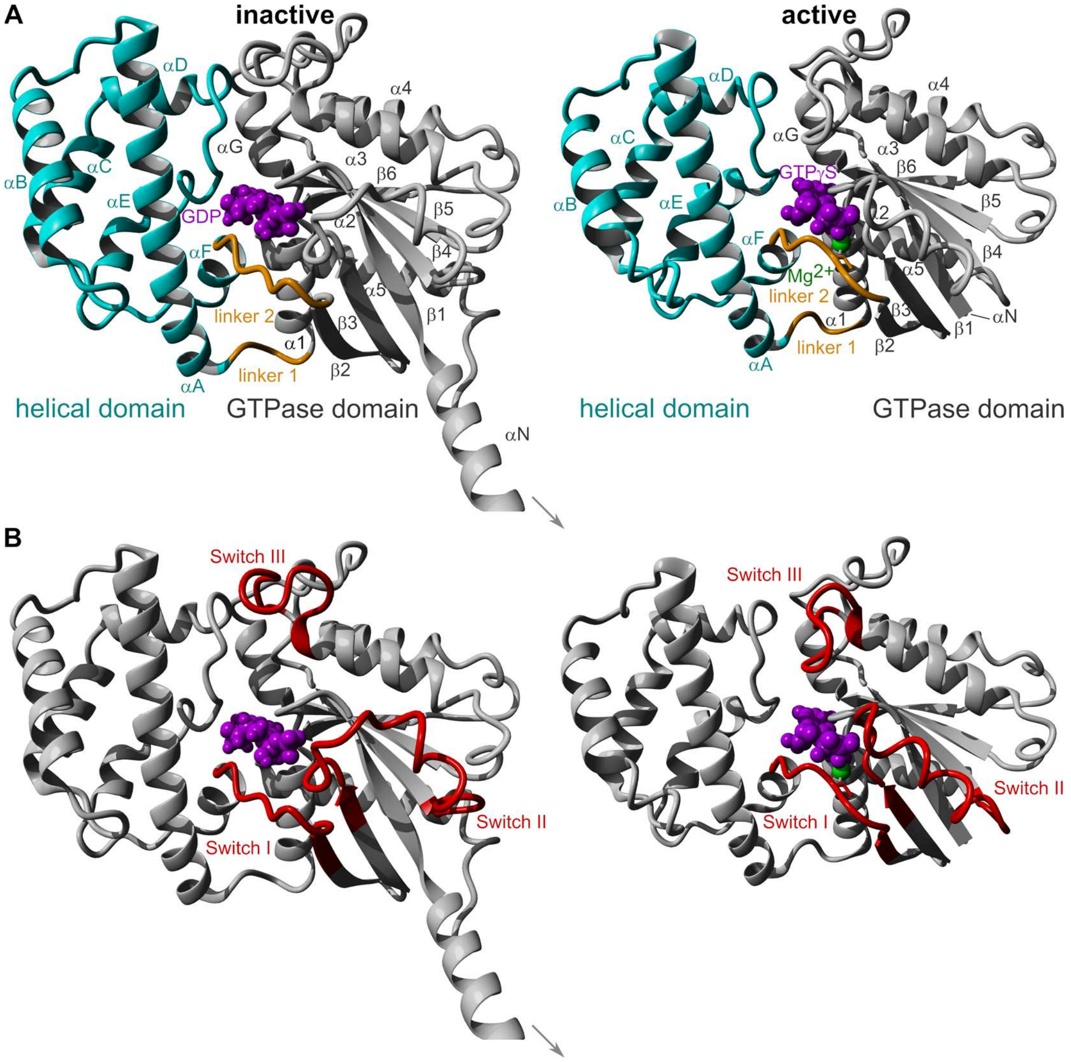


**Figure S2. Structural features of Gα proteins: A** shows the GTPase domain (grey), the helical domain (cyan), both linkers (orange) and the contained secondary structural elements for the inactive, GDP-bound (violet) Gαi1 homology model (from 3UMS[37] and 5JS8[38], used in entire left column) and the active, GTPγS-bound (violet) Gαi1 structure (1GIA[39], used in entire right column); in **B** the three switch regions (Switch I-III) are marked in red.

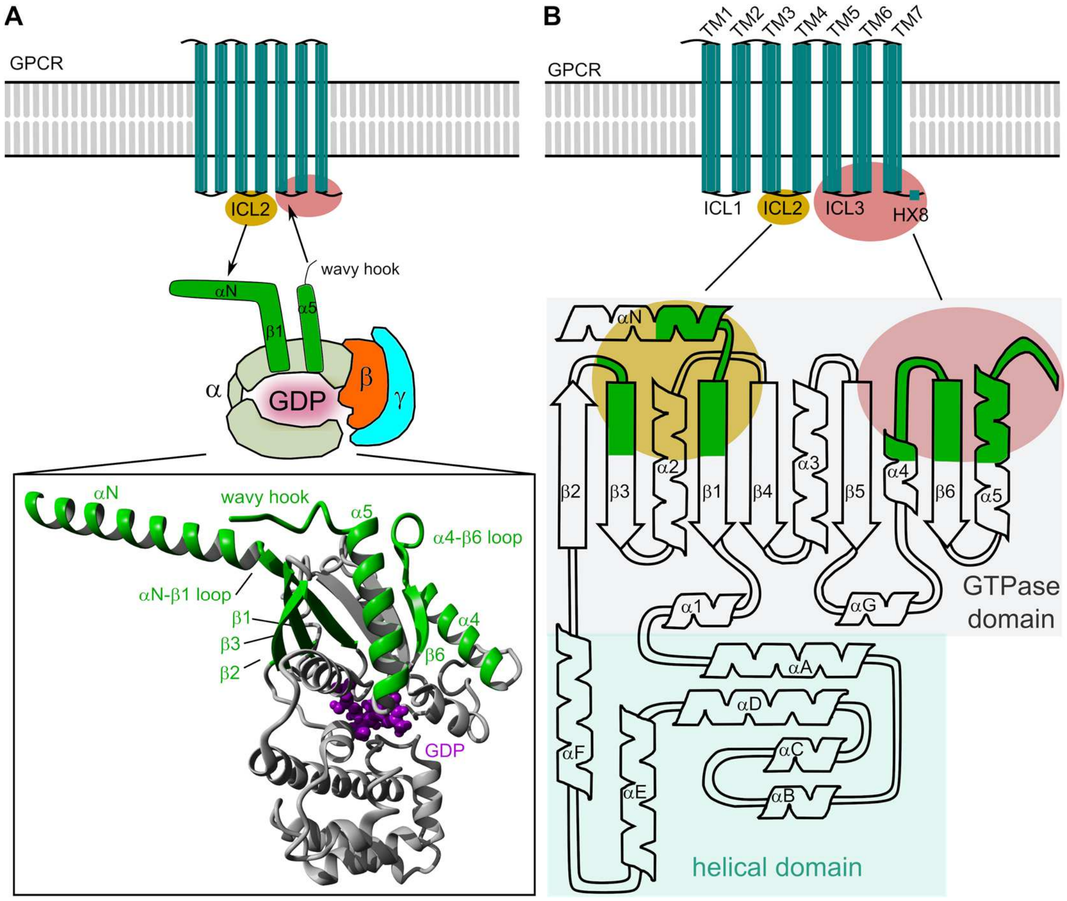


**Figure S3. Gα-GPCR interface. A**: Interactions of the Gα subunit with the GPCR. The primary interaction takes place between α5 and the wavy hook of the Gα subunit and the intracellular core of the GPCR. The second interface is established between the αN/β1 region of the Gα-subunit and the ICL2 of the GPCR. Homology model of Gαi1 (from 3UMS[37] and 5JS8[38]) in the orientation of the G protein. GPCR contact regions colored in green. **B**: Two-dimensional representation[40] of the domain arrangement of Gα proteins: Important Gα-GPCR interaction regions are marked in green (Gα), red (GPCR, area 1) and beige (GPCR area 2).

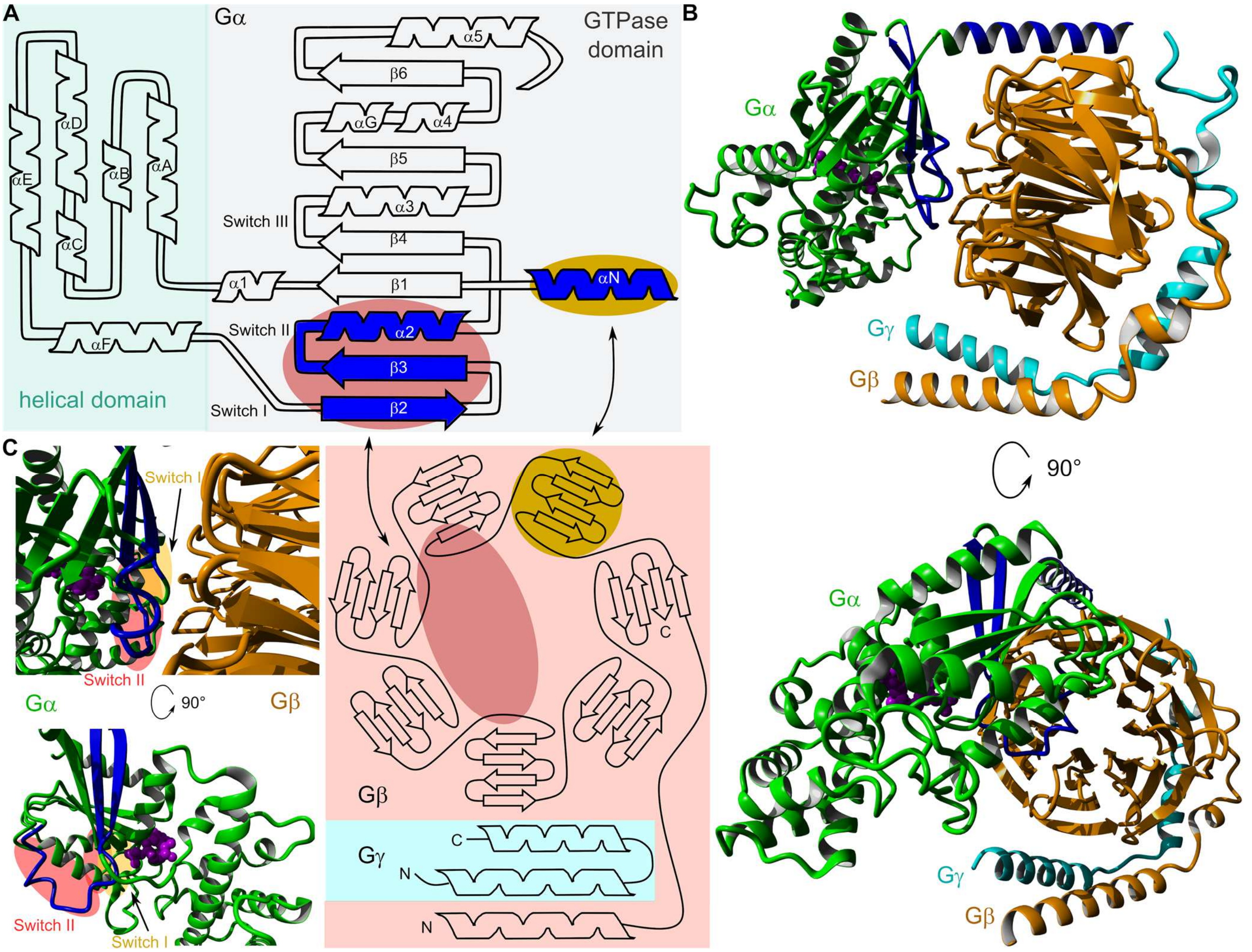


**Figure S4. Gα-Gβγ interface.** Two-dimensional representation[41] of the domain arrangement of Gα proteins and Gβγ dimers (**A**) and crystal structure of Gαt/iβγ (from 1GOT[41]) in different orientations with Gα (green), Gβ (orange) and Gγ (cyan) (**B**, **C**). Important Gα-Gβγ interaction regions are marked in blue (Gα, e.g., αN, β2, β3, β3α2, α2), red (interface 1) and beige (interface 2). In **C** the Gα-Gβγ interface 1 is presented with Switch I (yellow) and Switch II (red) labeled.

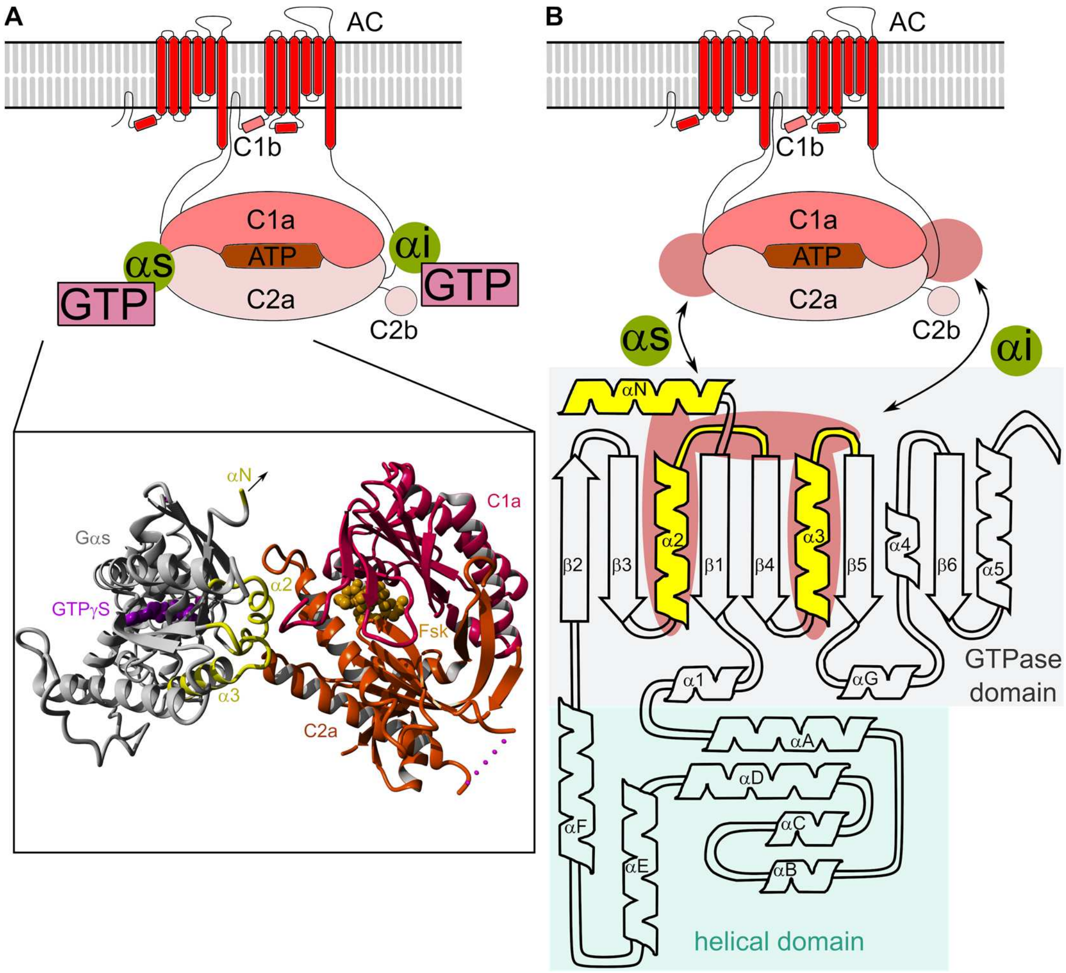


**Figure S5. Gα-AC interface. A**: Interactions of Gαs/i (green) with the adenylyl cyclase (AC, catalytic domains (C1a-b, C2a-b), red colors) derived from Tang et al. and Qi et al.[42,43]. Crystal structure (1AZS[44]) of Gαs (gray, GTPγS (violet)) in complex with the AC catalytic domains (C1a: red, C2a: orange, methylpiperazino-forskolin (Fsk): beige). The Gαs effector interface is colored in yellow (α2, α2-β4, α3, α3-β5). **B**: Two-dimensional representation[40] of the domain arrangement of Gα proteins: Important Gα-effector interaction regions are marked in yellow (Gα), the interface in red.

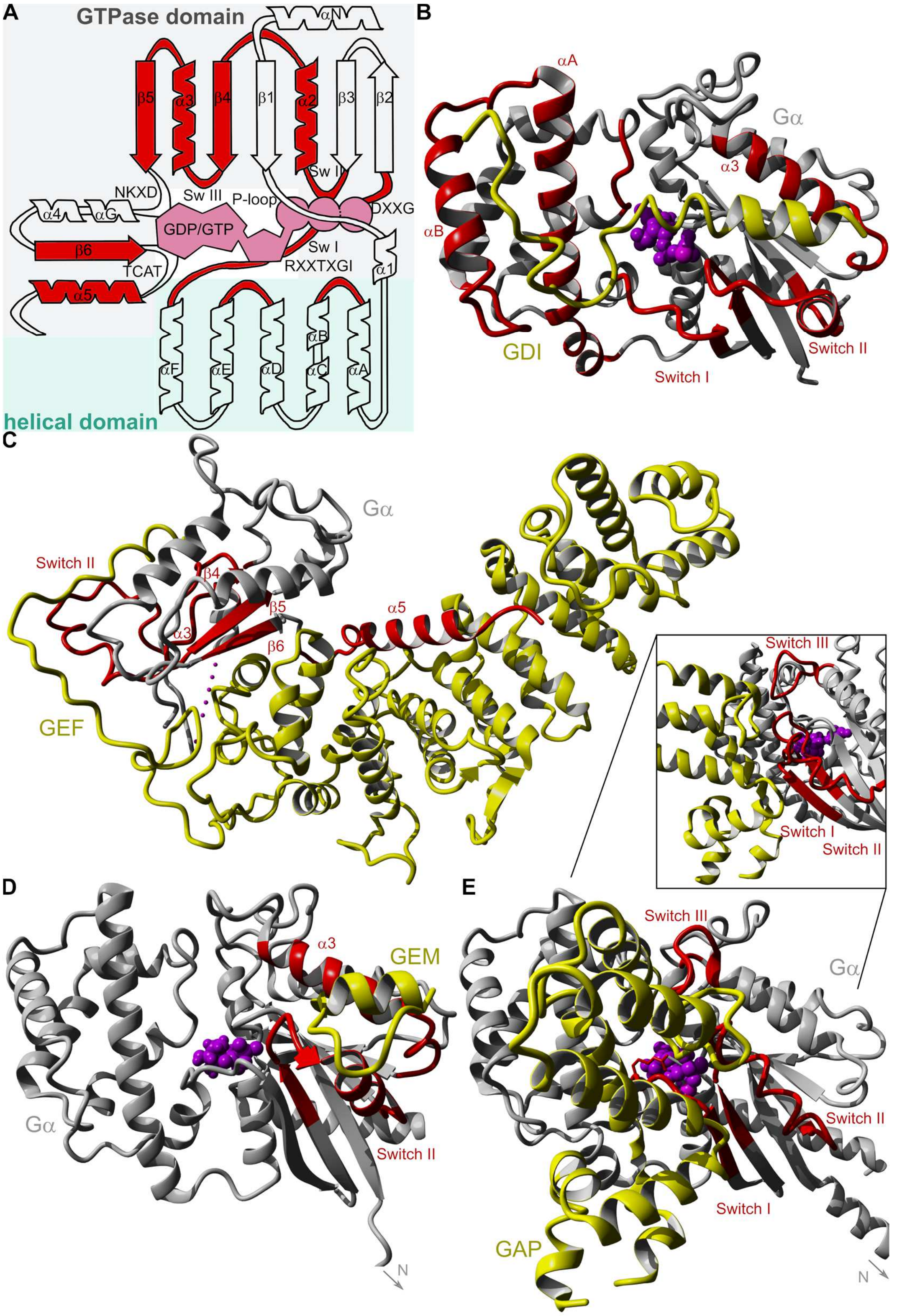


**Figure S6. Gα-accessory protein interface.** Two-dimensional representation[45] of the domain arrangement of Gα proteins with the five motifs crucial for nucleotide binding (**A**) and Gα crystal structures (gray) with different accessory proteins (yellow) bound (GDI: 1KJY[46] (**B**), GEF: 6VU8[47] (**C**), GEM: 6MHF[40] (**D**), GAP: 1AGR[48] (**E**)). Common contacts to the accessory proteins depicted in red.

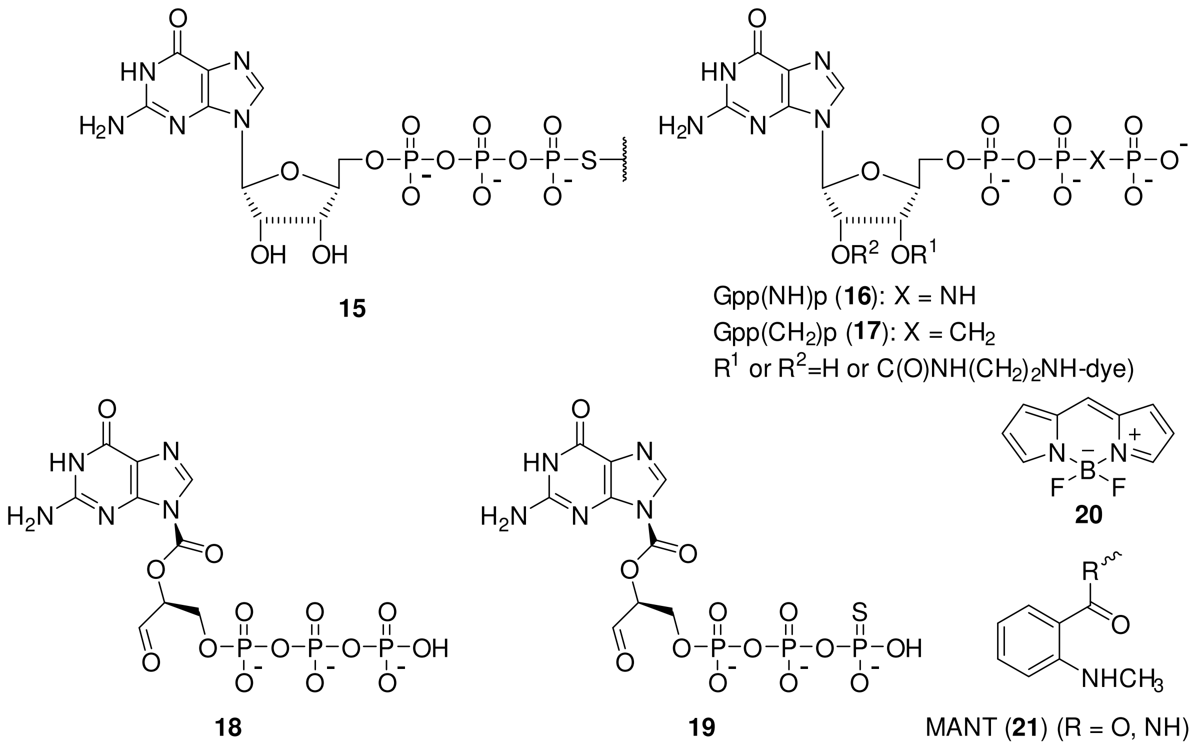


**Figure S7. Chemical structures of important guanine nucleotide analogs.** Guanosine-5'-O-(γ-thio)triphosphate (GTPγS, **15**), guanyl-5'-(β,γ-imino) triphosphate (Gpp(NH)p, **16**), guanyl-5'(β,γ-methylene) triphosphate (Gpp($CH_2$)p, **17**), (Gpp($CH_2$)p)2',3'-dialdehyde analog of GTP or GTPγS (oGTP, (**18**) and oGTPγS (**19**), respectively), and 4,4-difluoro-4-bora-3a,4a-diaza-s-indance (BODIPY, **20**), N-methyl-3'-*O*-antranoyl (MANT, **21**).[10–14]

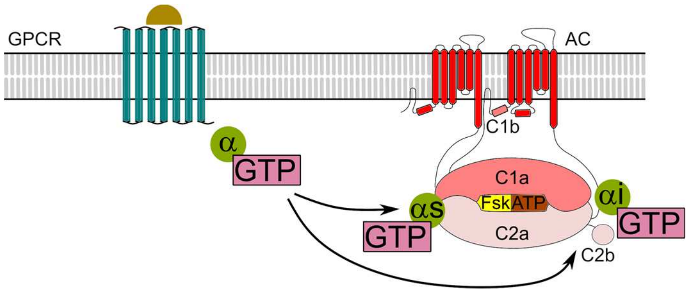


**Figure S8. Schematic description of adenylyl cyclase's catalytic domains (C1 and C2) derived from Tang et al. and Qi et al**.[42,43] Forskolin (Fsk) activates the AC and increases the concentration of cAMP in a similar mode of that of Gαs. The inhibitory protein Gαi binds to the C1 domain of the enzyme and attenuates the ATP conversion into cAMP.

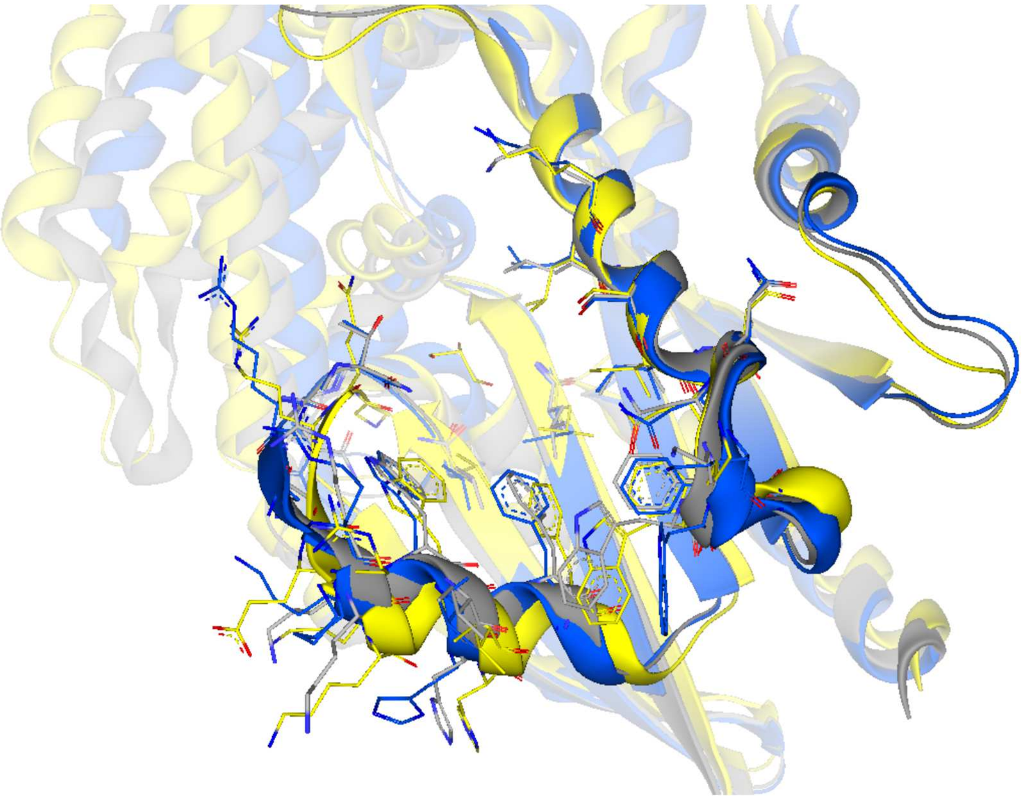

**Figure S9. Similarity of peptide binding sites between different Gα subunit structures.** The figure shows structural alignments of the peptide binding sites from 1Y3A (blue)[49], 6MHF (yellow)[40] and the homology model (from 3UMS[37] and 5JS8[38]) (grey) generated via the SIENA method that is specialized in finding structures with similar binding sites[50] implemented in the SeeSAR 10.2.1 (BioSolveIT GmbH, Sankt Augustin, Germany, 2020, www.biosolveit.de/SeeSAR) molecular modeling suite. The figure shows that the binding sites have near-identical overlap in of their backbones and only minor deviations in the orientations of their side chain residues.

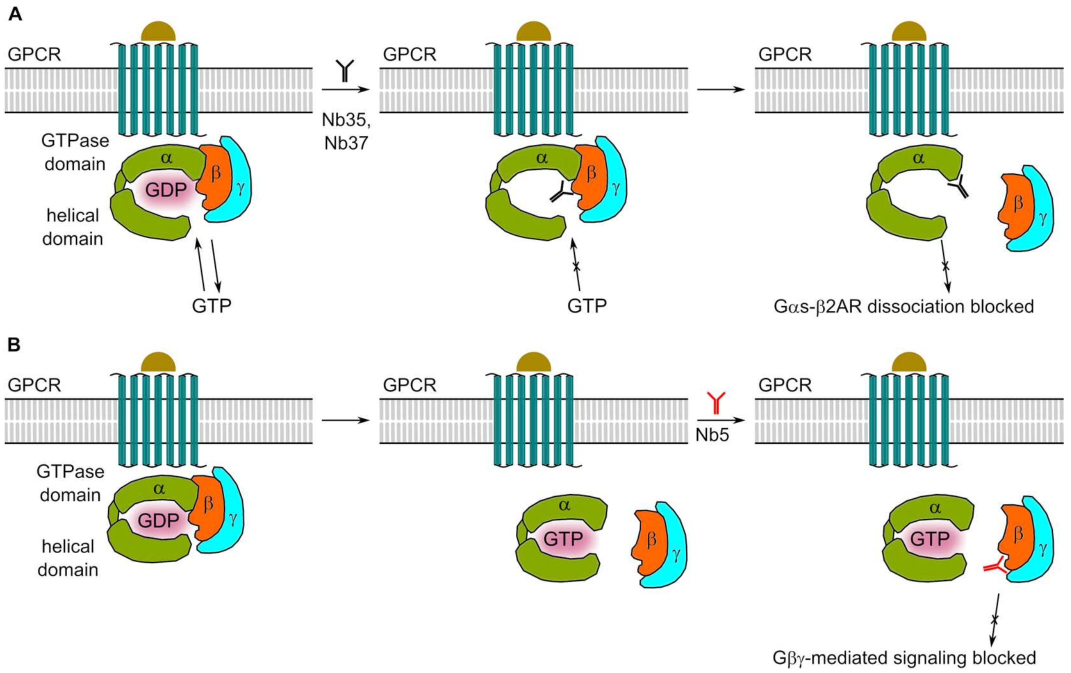


**Figure S10. Schematic description of nanobodies contribution in GPCR-G protein signaling. A**: The development of nanobodies (Nb) 35 and 37 led to the blockade of G protein dissociation from the $\beta_2$-adrenoreceptor (AR). More specifically, both Nbs bind in between the interface of Gαs-GTPase domain and Gβ and inhibit the GTP (or GTPγS) action.[31,32] **B**: In contradiction to model **A**, the Nb5 is the first reported Nb[34] that does not affect directly the Gαs activity. It binds the $G\beta_1\gamma_1$ complex restricting its physiologically mediated signaling.

## Supporting Tables

**Table S1.** High-throughput-derived peptides targeting the Gα-accessory protein interface. Core sequences are marked in bold.

| Name | Peptide sequence |
|---|---|
| R6A[51] | MSQTKRLDDQL**YWWEYL** |
| R6A-1[51] | DQL**YWWEYL** |
| AR6-05[52] | DESDPEELM**YWWEFL**SEDPSS |
| GSP[53] | MAMSDRNKRL**TVWEFL**ALPSST |
| mGSP-1[53] | MAMSDQNKRM**TVREFL**ALPSSL |
| mGSP-2[53] | MYTSDHNKLL**TVREFL**ALPSST |
| linGiBP[54] | MI**TWYEFV**AGTK |
| cycGiBP[54] | cyc[MI**TWYEFV**AGTK] |
| cycPRP-1[55] | cyc[MI**TWIDFI**SPSK] |
| cycPRP-3[55] | cyc[M**TWFEFL**SSTSK] |
| Gα SUPR peptide[56] | cyc[MF**Y(NMe-A)YEY(NMe-A)**QWSK] |
| GsIN-1[57] | cyc[YFESVYAIWGTLC] |
| Phage display peptide 1[58] | YPSMFHP |
| Phage display peptide 3[58] | LPALHGH |
| KB-752[49] | SRV**TWYDFL**MEDTKSR |
| KB-1753[59] | SSRGYYH**GIWVG**EEGRLSR |
| KB-1746[60] | SSSYSEH**CQRWGCY**ARLSR |
| KB-1755[60] | SSRLCPEW**ICPWE**WPASSR |
| GPM-1* | R**WLRYL**RYP |

* unpublished result.

## References


[1] P. C. Sternweis, J. K. Northup, E. Hanski, L. S. Schleifer, M. D. Smigel, A. G. Gilman, *Adv. Cyclic Nucleotide Res.* **1981**, *14*, 23.
[2] G. M. Bokoch, T. Katada, J. K. Northup, E. L. Hewlett, A. G. Gilman, *J. Biol. Chem.* **1983**, *258*, 2072.
[3] G. M. Bokoch, T. Katada, J. K. Northup, M. Ui, A. G. Gilman, *J. Biol. Chem.* **1984**, *259*, 3560.
[4] S. Paris, J. Pouysségur, *J. Biol. Chem.* **1990**, *265*, 11567.
[5] T. Higashijima, K. M. Ferguson, P. C. Sternweis, E. M. Ross, M. D. Smigel, A. G. Gilman, *J. Biol. Chem.* **1987**, *262*, 752.
[6] T. Higashijima, K. M. Ferguson, M. D. Smigel, A. G. Gilman, *J. Biol. Chem.* **1987**, *262*, 757.
[7] T. Higashijima, K. M. Ferguson, P. C. Sternweis, M. D. Smigel, A. G. Gilman, *J. Biol. Chem.* **1987**, *262*, 762.
[8] a) M. R. Ahmadian, U. Hoffmann, R. S. Goody, A. Wittinghofer, *Biochemistry* **1997**, *36*, 4535; b) L. Kallal, R. Fishel, *Yeast* **2000**, *16*, 387.
[9] N. Minadeo, B. Layden, L. V. Amari, V. Thomas, K. Radloff, C. Srinivasan, H. E. Hamm, D. M. de Freitas, *Arch. Biochem. Biophys.* **2001**, *388*, 7.
[10] C. Nanoff, S. Boehm, M. Hohenegger, W. Schütz, M. Freissmuth, *J. Biol. Chem.* **1994**, *269*, 31999.
[11] M. Hohenegger, C. Nanoff, H. Ahorn, M. Freissmuth, *J. Biol. Chem.* **1994**, *269*, 32008.
[12] A. E. Remmers, R. Posner, R. R. Neubig, *J. Biol. Chem.* **1994**, *269*, 13771.
[13] A. E. Remmers, R. R. Neubig, *J. Biol. Chem.* **1996**, *271*, 4791.
[14] D. P. McEwen, K. R. Gee, H. C. Kang, R. R. Neubig, *Anal. Biochem.* **2001**, *291*, 109.
[15] A. Treibs, F.-H. Kreuzer, *Justus Liebigs Annalen der Chemie* **1968**, *718*, 208.
[16] I. Jarmoskaite, I. AlSadhan, P. P. Vaidyanathan, D. Herschlag, *eLIFE* **2020**, *9*.
[17] a) D. G. Lambright, J. P. Noel, H. E. Hamm, P. B. Sigler, *Nature* **1994**, *369*, 621; b) J. Sondek, D. G. Lambright, J. P. Noel, H. E. Hamm, P. B. Sigler, *Nature* **1994**, *372*, 276; c) J. P. Noel, H. E. Hamm, P. B. Sigler, *Nature* **1993**, *366*, 654.
[18] H. E. Hamm, D. Deretic, K. P. Hofmann, A. Schleicher, B. Kohl, *J. Biol. Chem.* **1987**, *262*, 10831.
[19] D. Deretic, H. E. Hamm, *J. Biol. Chem.* **1987**, *262*, 10839.
[20] F. R. McKenzie, E. C. Kelly, C. G. Unson, A. M. Spiegel, G. Milligan, *Biochem. J.* **1988**, *249*, 653.
[21] M. Pines, P. Gierschik, G. Milligan, W. Klee, A. Spiegel, *Proc. Natl. Acad. Sci. U. S. A.* **1985**, *82*, 4095.
[22] J. A. Hsia, J. Moss, E. L. Hewlett, M. Vaughan, *J. Biol. Chem.* **1984**, *259*, 1086.
[23] G. Milligan, *Biochem. J.* **1988**, *255*, 1.
[24] W. F. Simonds, P. K. Goldsmith, J. Codina, C. G. Unson, A. M. Spiegel, *Proc. Natl. Acad. Sci. U. S. A.* **1989**, *86*, 7809.
[25] B. Rouot, J. Carrette, M. Lafontan, P. Lan Tran, J. A. Fehrentz, J. Bockaert, M. Toutant, *Biochem. J.* **1989**, *260*, 307.
[26] R. Murray-Whelan, W. Schlegel, *J. Biol. Chem.* **1992**, *267*, 2960.
[27] J. R. Lane, D. Henderson, B. Powney, A. Wise, S. Rees, D. Daniels, C. Plumpton, I. Kinghorn, G. Milligan, *FASEB J.* **2008**, *22*, 1924.
[28] P. Sánchez-Blázquez, J. Garzón, *Life Sciences* **1994**, *55*, PL445-PL450.
[29] R. Weingarten, L. Ransnäs, H. Mueller, L. A. Sklar, G. M. Bokoch, *J. Biol. Chem.* **1990**, *265*, 11044.
[30] T. Higashijima, S. Uzu, T. Nakajima, E. M. Ross, *J. Biol. Chem.* **1988**, *263*, 6491.
[31] S. G. F. Rasmussen, B. T. DeVree, Y. Zou, A. C. Kruse, K. Y. Chung, T. S. Kobilka, F. S. Thian, P. S. Chae, E. Pardon, D. Calinski et al., *Nature* **2011**, *477*, 549.
[32] S. G. F. Rasmussen, H.-J. Choi, J. J. Fung, E. Pardon, P. Casarosa, P. S. Chae, B. T. DeVree, D. M. Rosenbaum, F. S. Thian, T. S. Kobilka et al., *Nature* **2011**, *469*, 175.
[33] S. Maeda, A. Koehl, H. Matile, H. Hu, D. Hilger, G. F. X. Schertler, A. Manglik, G. Skiniotis, R. J. P. Dawson, B. K. Kobilka, *Nat. Commun.* **2018**, *9*.
[34] S. Gulati, H. Jin, I. Masuho, T. Orban, Y. Cai, E. Pardon, K. A. Martemyanov, P. D. Kiser, P. L. Stewart, C. P. Ford et al., *Nat. Commun.* **2018**, *9*, 1996.
[35] A. Manglik, B. K. Kobilka, J. Steyaert, *Annu. Rev. Pharmacol. Toxicol.* **2017**, *57*, 19.
[36] R. Lappano, M. Maggiolini, *Nat. Rev. Drug Discov.* **2011**, *10*, 47.
[37] N. A. Lambert, C. A. Johnston, S. D. Cappell, S. Kuravi, A. J. Kimple, F. S. Willard, D. P. Siderovski, *Proc. Natl. Acad. Sci. U. S. A.* **2010**, *107*, 7066.
[38] D. Goricanec, R. Stehle, P. Egloff, S. Grigoriu, A. Plückthun, G. Wagner, F. Hagn, *Proc. Natl. Acad. Sci. U. S. A.* **2016**, *113*, E3629-38.
[39] D. E. Coleman, A. M. Berghuis, E. Lee, M. E. Linder, A. G. Gilman, S. R. Sprang, *Science* **1994**, *265*, 1405.
[40] N. A. Kalogriopoulos, S. D. Rees, T. Ngo, N. J. Kopcho, A. V. Ilatovskiy, N. Sun, E. A. Komives, G. Chang, P. Ghosh, I. Kufareva, *Proc. Natl. Acad. Sci. U. S. A.* **2019**, *116*, 16394.
[41] D. G. Lambright, J. Sondek, A. Bohm, N. P. Skiba, H. E. Hamm, P. B. Sigler, *Nature* **1996**, *379*, 311.
[42] W. J. Tang, J. H. Hurley, *Mol. Pharmacol.* **1998**, *54*, 231.
[43] C. Qi, S. Sorrentino, O. Medalia, V. M. Korkhov, *Science* **2019**, *364*, 389.
[44] J. J. Tesmer, R. K. Sunahara, A. G. Gilman, S. R. Sprang, *Science* **1997**, *278*, 1907.
[45] A. I. de Opakua, K. Parag-Sharma, V. DiGiacomo, N. Merino, A. Leyme, A. Marivin, M. Villate, L. T. Nguyen, M. A. de La Cruz-Morcillo, J. B. Blanco-Canosa et al., *Nat. Commun.* **2017**, *8*, 15163.
[46] R. J. Kimple, M. E. Kimple, L. Betts, J. Sondek, D. P. Siderovski, *Nature* **2002**, *416*, 878.
[47] A. B. Seven, D. Hilger, M. M. Papasergi-Scott, L. Zhang, Q. Qu, B. K. Kobilka, G. G. Tall, G. Skiniotis, *Cell Rep.* **2020**, *30*, 3699-3709.e6.
[48] J. J. Tesmer, D. M. Berman, A. G. Gilman, S. R. Sprang, *Cell* **1997**, *89*, 251.
[49] C. A. Johnston, F. S. Willard, M. R. Jezyk, Z. Fredericks, E. T. Bodor, M. B. Jones, R. Blaesius, V. J. Watts, T. K. Harden, J. Sondek et al., *Structure* **2005**, *13*, 1069.
[50] a) S. Bietz, M. Rarey, *J. Chem. Inf. Model.* **2015**, *55*, 1747; b) S. Bietz, M. Rarey, *J. Chem. Inf. Model.* **2016**, *56*, 248.
[51] W. W. Ja, R. W. Roberts, *Biochemistry* **2004**, *43*, 9265.
[52] W. W. Ja, O. Wiser, R. J. Austin, L. Y. Jan, R. W. Roberts, *ACS Chem. Biol.* **2006**, *1*, 570.
[53] R. J. Austin, W. W. Ja, R. W. Roberts, *J. Mol. Biol.* **2008**, *377*, 1406.
[54] S. W. Millward, S. Fiacco, R. J. Austin, R. W. Roberts, *ACS Chem. Biol.* **2007**, *2*, 625.
[55] S. M. Howell, S. V. Fiacco, T. T. Takahashi, F. Jalali-Yazdi, S. W. Millward, B. Hu, P. Wang, R. W. Roberts, *Sci. Rep.* **2014**, *4*, 6008.
[56] S. V. Fiacco, L. E. Kelderhouse, A. Hardy, Y. Peleg, B. Hu, A. Ornelas, P. Yang, S. T. Gammon, S. M. Howell, P. Wang et al., *Chembiochem* **2016**, *17*, 1643.
[57] S. A. Dai, Q. Hu, R. Gao, A. Lazar, Z. Zhang, M. von Zastrow, H. Suga, K. M. Shokat, **2020**, *bioRixv preprint,* DOI 10.1101/2020.04.25.054080.
[58] J. Hessling, M. J. Lohse, K.-N. Klotz, *Biochem. Pharmacol.* **2003**, *65*, 961.
[59] C. A. Johnston, E. S. Lobanova, A. S. Shavkunov, J. Low, J. K. Ramer, R. Blaesius, Z. Fredericks, F. S. Willard, B. Kuhlman, V. Y. Arshavsky et al., *Biochemistry* **2006**, *45*, 11390.
[60] C. A. Johnston, F. S. Willard, J. K. Ramer, R. Blaesius, C. N. Roques, D. P. Siderovski, *Comb. Chem. High Throughput Screen.* **2008**, *11*, 370.


## Author Contributions

All authors contributed to the final manuscript and have given approval to the final version.
